# Systematics of stalked jellyfishes (Cnidaria: Staurozoa)

**DOI:** 10.7717/peerj.1951

**Published:** 2016-05-05

**Authors:** Lucília S. Miranda, Yayoi M. Hirano, Claudia E. Mills, Audrey Falconer, David Fenwick, Antonio C. Marques, Allen G. Collins

**Affiliations:** 1 Departamento de Zoologia, Instituto de Biociências, Universidade de São Paulo, São Paulo, Brazil; 2 Coastal Branch of Natural History Museum and Institute, Chiba, Katsuura, Chiba, Japan; 3 Friday Harbor Laboratories and the Department of Biology, University of Washington, Friday Harbor, Washington, United States of America; 4 Marine Research Group of the Field Naturalists Club of Victoria, Melbourne, Victoria, Australia; 5 Sciences Department, Museum Victoria, Melbourne, Victoria, Australia; 6 Penzance, Cornwall, England, United Kingdom; 7 Centro de Biologia Marinha, Universidade de São Paulo, São Sebastião, São Paulo, Brazil; 8 National Systematics Laboratory, National Marine Fisheries Service (NMFS), National Museum of Natural History, Smithsonian Institution, Washington, D.C., United States of America

**Keywords:** Evolution, Taxonomy, Phylogeny, Medusozoa, Stauromedusae

## Abstract

Staurozoan classification is highly subjective, based on phylogeny-free inferences, and suborders, families, and genera are commonly defined by homoplasies. Additionally, many characters used in the taxonomy of the group have ontogenetic and intraspecific variation, and demand new and consistent assessments to establish their correct homologies. Consequently, Staurozoa is in need of a thorough systematic revision. The aim of this study is to propose a comprehensive phylogenetic hypothesis for Staurozoa, providing the first phylogenetic classification for the group. According to our working hypothesis based on a combined set of molecular data (mitochondrial markers COI and 16S, and nuclear markers ITS, 18S, and 28S), the traditional suborders Cleistocarpida (animals with claustrum) and Eleutherocarpida (animals without claustrum) are not monophyletic. Instead, our results show that staurozoans are divided into two groups, herein named Amyostaurida and Myostaurida, which can be distinguished by the absence/presence of interradial longitudinal muscles in the peduncle, respectively. We propose a taxonomic revision at the family and genus levels that preserves the monophyly of taxa. We provide a key for staurozoan genera and discuss the evolution of the main characters used in staurozoan taxonomy.

## Introduction

**Figure 1:**
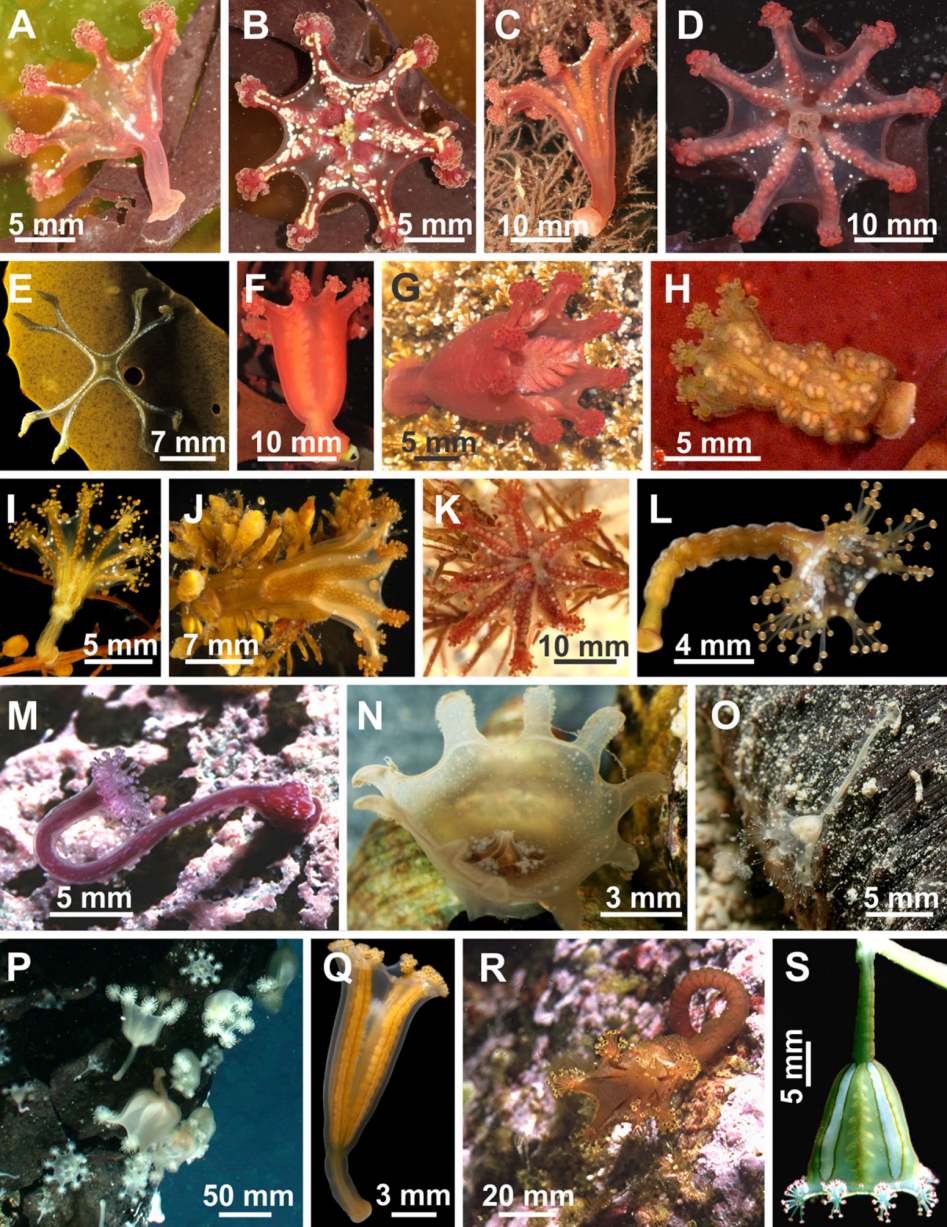
Diversity of stalked jellyfishes. *Calvadosia cruxmelitensis*: (A) lateral view, (B) oral view (photo credit: David Fenwick); *Calvadosia campanulata*: (C) lateral view, (D) oral view (photo credit: David Fenwick); *Calvadosia nagatensis*: (E) oral view (photo credit: Yayoi Hirano); *Craterolophus convolvulus*: (F, G) lateral view (photo credit: David Fenwick); *Depastromorpha africana*: (H) lateral view (photo credit: Yayoi Hirano); *Haliclystus tenuis*: (I) lateral view (photo credit: Yayoi Hirano); *Haliclystus borealis*: (J) lateral view (photo credit: Yayoi Hirano); *Haliclystus octoradiatus*: (K) oral view (photo credit: David Fenwick); *Haliclystus inabai*: (L) lateral view (photo credit: Yayoi Hirano); *Kyopoda lamberti*: (M) lateral view (photo credit: courtesy of Ronald Shimek); *Lipkea* sp. Japan: (N) oral view (photo credit: Yayoi Hirano); *Stylocoronella riedli*: (O) lateral view (proto credit: courtesy of Mat Vestjens and Anne Frijsinger); *Lucernaria janetae*: (P) lateral and oral views (photo credit: courtesy of Richard Lutz); *Manania uchidai*: (Q) lateral view (photo credit: Yayoi Hirano); *Manania gwilliami*: (R) oral view (photo credit: courtesy of Ronald Shimek); *Manania handi*: (S) lateral view (photo credit: Claudia Mills).

**Figure 2:**
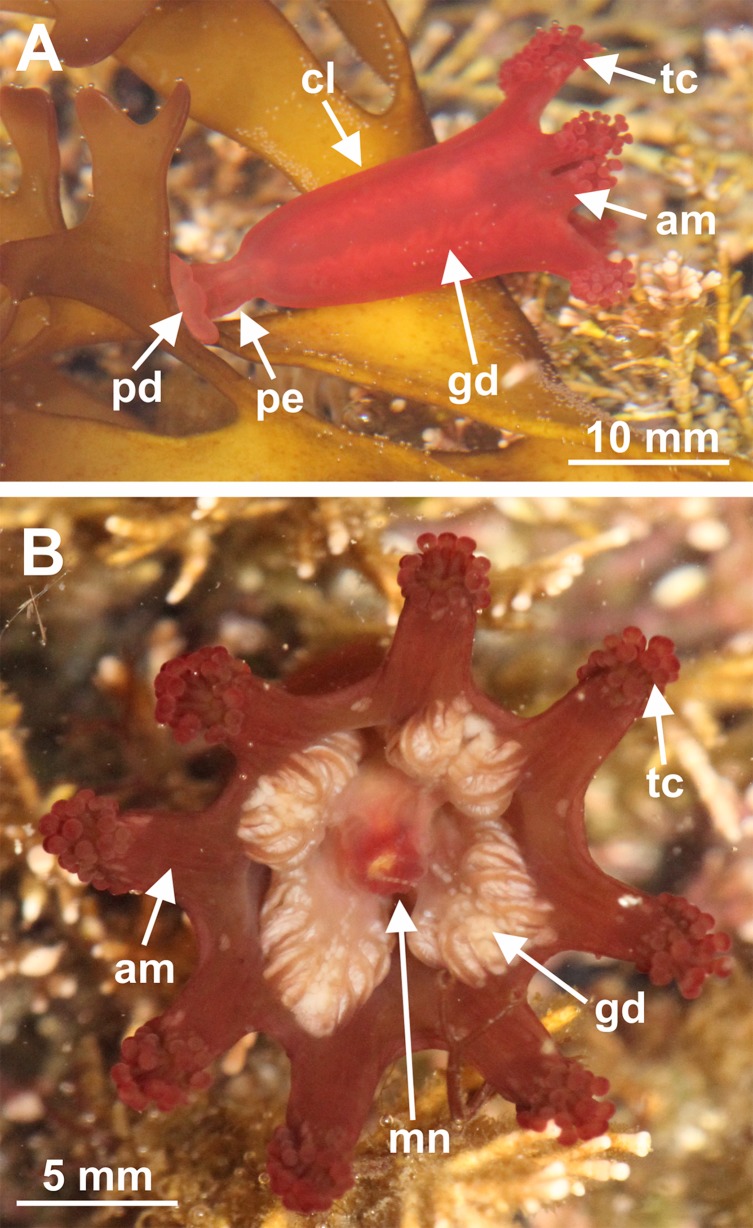
General external anatomy of stalked jellyfishes. *Craterolophus convolvulus*: (A) lateral view, (B) oral view. Abbreviations: am, arm; cl, calyx; gd, gonad; mn, manubrium; pd, pedal disk; pe, peduncle; tc, tentacle cluster. Photo credit: David Fenwick.

Staurozoa is a class of benthic cnidarians, the so-called stalked jellyfishes (Figs. 1 and 2), represented by approximately 50 species (Clark, 1863; Kramp, 1961; Daly et al., 2007). However, from the first stauromedusan species described (*Lucernaria quadricornis* Müller, 1776) until their proposition as the fifth class of Cnidaria (Marques & Collins, 2004), the group has had a long history of classifications, being labeled as a “puzzling group” (Gwilliam, 1956). While one species was erroneously first placed among sea cucumbers (*Manania auricula* as *Holothuria lagenam referens* Müller, 1776), most assessments prior to the 1850’s assumed that they were closely related to sea anemones (Cuvier, 1817; Cuvier, 1830) until Sars (1846) noted that the presence of gastric cirri suggested that they were allied with the jellyfishes. Reflecting this thinking, Goette (1887) included Stauromedusae as a suborder within Scyphozoa, a position that was only recently challenged. Marques & Collins (2004) proposed the class based on a phylogenetic analysis of morphological and life cycle traits, as the clade uniting the fossil group Conulatae and the Stauromedusae. In light of further evidence from the fossil record, a subsequent analysis of a similar dataset contradicted the hypothesis that Conulatae and Stauromedusae form a clade, and proposed the composition of Staurozoa to consist exclusively of the extant Stauromedusae (Van Iten et al., 2006). The same analysis suggested that Staurozoa is the sister group to all other medusozoans (Cubozoa, Hydrozoa, and Scyphozoa), a result corroborated by analyses of nuclear ribosomal data (Collins et al., 2006; see also Van Iten et al., 2014). In contrast, however, analyses of complete mitochondrial genome data (Kayal et al., 2013) suggest that Staurozoa may be the sister group of Cubozoa, and more recent phylogenomic analyses support a clade formed by Staurozoa, Cubozoa, and Scyphozoa (Zapata et al., 2015), demonstrating that more studies are necessary to reach a stable topology for Cnidaria.

Although evolutionary studies have supported monophyly of the class (Collins & Daly, 2005; Collins et al., 2006; Kayal et al., 2013), comparatively little effort has been applied toward determining the systematic relationships among species of Staurozoa, with rare exceptions (Collins & Daly, 2005; Lutz et al., 2006). The current classification of Staurozoa is mainly based on the proposals of Clark (1863), Haeckel (1879), Uchida (1929) and Carlgren (1935), and is completely focused on anatomical features. Uchida (1973) proposed a hypothesis of relationship among families of stalked jellyfishes based on the characters that he regarded as important, but this analysis was not derived from specific evolutionary methods. A recent molecular inference, with limited taxon sampling, demonstrated the need for reassessing suprageneric clades, because several were found not to be monophyletic (Collins & Daly, 2005). Additionally, many characters used in the taxonomy of the group have ontogenetic and intraspecific variation, and demand consistent assessments and clarifications to establish their correct homologies (Miranda, Morandini & Marques, 2009). Consequently, staurozoan classification and taxonomy is subjective, based on phylogeny-free inferences, and families and genera may be commonly defined by homoplasies (Collins & Daly, 2005). Therefore, Staurozoa is in need of a thorough systematic revision.

Inferences about the relationships among staurozoan species are especially important because of the phylogenetic status and position of Staurozoa, as a distinct clade separate from the other major cnidarian groups (Anthozoa, Cubozoa, Hydrozoa, and Scyphozoa) (Collins et al., 2006; Van Iten et al., 2006; Kayal et al., 2013; Zapata et al., 2015). The peculiar life cycle of staurozoans (Wietrzykowski, 1912; Kikinger & Salvini-Plawen, 1995; Miranda, Collins & Marques, 2010) is tightly connected to their unique anatomy, in which characters of polypoid and medusoid stages are present in the same stauromedusa (Miranda, Collins & Marques, 2013). Our expectation is that a better understanding and interpretation of the character evolution within the group will provide crucial information for inferences in cnidarian evolution.

Therefore, it is of the utmost importance to carry out an evolutionary analysis encompassing a large number of species of Staurozoa. This study presents the most comprehensive phylogenetic hypothesis for Staurozoa yet proposed and provides the first phylogenetic classification for the group. Further, we provide a key for staurozoan genera and discuss evolution of the main characters used in staurozoan taxonomy.

## Material and Methods

### Molecular

Twenty-four species from ten genera, plus eight non-identified species (identified to genus level), from different regions of the world, were used in the molecular analyses (Table 1). Tissue samples from the tentacle clusters (or marginal lobes for *Lipkea* spp.) were removed and preserved in 90–100% ethanol, and stored at −20 °C. DNA extractions were carried out with InstaGene (Bio-Rad) at the Universidade de São Paulo, Instituto de Biociências (IB-USP, Brazil), or using an organic phenol-chloroform method on the automated DNA isolation system, AutoGenPrep 965 (AutoGen Inc., Holliston, MA, USA) at the Smithsonian’s Laboratories of Analytical Biology (LAB, USA), following the manufacturers’ protocols. Genes were amplified using PCR, then purified with AMPure® (Agencourt®) or ExoSAP. Different molecular markers (mitochondrial COI and 16S; nuclear ITS–ITS1+5.8S+ITS2, 18S, and 28S) were targeted for analyses (Tables 2 and 3). These markers were previously adopted and have been shown to be efficient for evolutionary studies in medusozoans (Dawson, 2004; Collins et al., 2006; Collins et al., 2008; Miranda, Collins & Marques, 2010; Nawrocki et al., 2013; Cunha, Genzano & Marques, 2015). DNA sequencing was done using the BigDye® Terminator v3.1 kit (Applied Biosystems) and the same primers used for PCR (Table 2). The procedure was carried out on an ABI PRISM® 3100 Genetic Analyzer (Hitachi). Samples were extracted, amplified and sequenced at LAB (USA) and IB-USP (Brazil). Out-group sequences (Anthozoa, Cubozoa, Hydrozoa, and Scyphozoa) were obtained in GenBank (Table 4).

**Table 1:** Species used in the phylogenetic analyses (parsimony, maximum likelihood, and Bayesian inference). Based on mitochondrial molecular markers (COI and 16S) and nuclear molecular markers (ITS, 18S, and 28S).

Species	Specimens	GenBank accession numbers					Locality	Voucher
		COI	16S	ITS	18S	28S		
*Craterolophus convolvulus*	*Craterolophus convolvulus* GER	KU257472	AY845343	KU308618	AY845344	AY920781	Helgoland, North Sea, Germany	USNM 1073330, 1073339
	*Craterolophus convolvulus* UK	KU257473	KU257497	KU308619	–	–	Sennen Cove, Cornwall, England	USNM 1286315
	*Craterolophus convolvulus* USA	–	KU257498	KU308620	KU308557	KU308586	Rye Beach, Rye, New Hampshire, USA	MZUSP 002730
*Depastromorpha africana*	*Depastromorpha africana* 1*	–	–	–	KU308558	–	Kalk Bay, Cape Town, South Africa	MZUSP 002733
	*Depastromorpha africana* 2*	KU257474	KU257499	KU308621	–	KU308587	Kalk Bay, Cape Town, South Africa	MZUSP 002734
*Haliclystus antarcticus*	*Haliclystus antarcticus* ANT	KU257475	EU294003	FJ874779	EU247811	KU308588	Argentine antarctic station Jubany, King George Island, Antarctica	None
	*Haliclystus antarcticus* Chile	–	AY845340	KU308622	AY845348	KU308589	Valdivia, Chile	None
*Haliclystus borealis*	*Haliclystus borealis*	–	KU257500	KU308623	KU308559	KU308590	Muroran, Hokkaido, Japan	USNM 1106650
*Haliclystus californiensis*	*Haliclystus californiensis*	GU201831	GU201829	KU308624	KU308560	KU308591	Otter Point, Pacific Grove, California, USA	USNM 1106657
*Haliclystus octoradiatus*	*Haliclystus octoradiatus*	KU257476	KU257501	KU308625	KU308561	KU308592	Cornwall, England	USNM 1286385
*Haliclystus “sanjuanensis”*	*Haliclystus “sanjuanensis”*	KU257477	HM022151	HM022145	KU308562	KU308593	San Juan Island, Washington, USA	USNM 1106935
*Haliclystus stejnegeri*	*Haliclystus stejnegeri*	KU257478	HM022153	HM022147	KU308563	KU308594	Daikokujima, Akkeshi, Hokkaido, Japan	KUNHM 002673-B
*Haliclystus tenuis*	*Haliclystus tenuis*	KU257479	HM022154	HM022148	KU308564	KU308595	Muroran, Hokkaido, Japan	USNM 1106651
*Kishinouyea corbini*	*Kishinouyea corbini*	–	KU257502	–	KU308565	KU308596	Aracruz, Espirito Santo, Brazil	MZUSP 1563
*Kishinouyea nagatensis*	*Kishinouyea nagatensis*	–	–	–	–	KU308597	Shimoda, Shizuoka, Japan	USNM 1106985
*Lipkea ruspoliana*	*Lipkea ruspoliana* 1*	–	KU257503	KU308626	KU308566	KU308598	Aquarium, Oceanographic Museum of Monaco	USNM 1315313
	*Lipkea ruspoliana* 2*	KU257480	–	–	–	–	Aquarium, Oceanographic Museum of Monaco	USNM 1315317
*Lucernaria bathyphila*	*Lucernaria bathyphila* Arctic	–	KU257504	–	KU308567	–	Arctic, Mid-Ocean Ridge	USNM 1301002
	*Lucernaria bathyphila* Deep Sea	KU257481	KU257505	KU308627	–	KU308599	Faroe-Shetland Channel between Faroe and Shetland Islands	USNM 1300113
*Lucernaria janetae*	*Lucernaria janetae* 1*	–	AY845342	FJ874778	AY845345	KU308600	East Pacific Rise	FMNH 10329
	*Lucernaria janetae* 2*	JN700946	–	–	–	–	East Pacific Rise	None
*Lucernaria quadricornis*	*Lucernaria quadricornis* 1*	–	–	–	–	KU308601	Keret Inlet, off Sredny Island, Black Rock, White Sea, Russia	USNM 1106636
	*Lucernaria quadricornis* 2*	–	KU257506	KU308628	KU308568	–	Near White Sea Biological Station of Moscow State University, White Sea, Russia	USNM 1102441
*Lucernaria sainthilairei*	*Lucernaria sainthilairei* 1*	–	–	–	KU308569	–	Near White Sea Biological Station of Moscow State University, White Sea, Russia	USNM 1106245
	*Lucernaria sainthilairei* 2*	KU257482	KU257507	KU308629	–	KU308602	Nicolskaya Inlet, off Bolshoy Medvedok Island, Kandalaksha Bay, White Sea, Russia	USNM 1106643
*Lucernariopsis campanulata*	*Lucernariopsis campanulata*	KU257483	KU257508	KU308630	KU308570	KU308603	Battery Rocks, Penzance, Cornwall, England	USNM 1286317
*Lucernariopsis cruxmelitensis*	*Lucernariopsis cruxmelitensis*	KU257484	KU257509	KU308631	KU308571	KU308604	Penzance, Cornwall, England	USNM 1286321
*Lucernariopsis tasmaniensis*	*Lucernariopsis tasmaniensis*	–	KU257510	KU308632	KU308572	–	Kitty Miller Bay, Phillip Island, Victoria, Australia	MV F158628
*Lucernariopsis vanhoeffeni*	*Lucernariopsis vanhoeffeni*	KU257485	KU257511	KU308633	KU308573	KU308605	Trinity Island, Palmer Archipelago, Antarctica	USNM 1106658
*Manania gwilliami*	*Manania gwilliami* 1*	–	KU257512	KU308634	KU308574	KU308606	Monterey Harbor, California, USA	USNM 1106649
	*Manania gwilliami* 2*	KU257486	–	–	–	–	Pacific Grove Marine Gardens Park, California, USA	USNM 1106662
*Manania uchidai*	*Manania uchidai*	–	KU257513	KU308635	KU308575	KU308607	Daikokujima, Akkeshi, Hokkaido, Japan	KUNHM 002673-A
*Sasakiella cruciformis*	*Sasakiella cruciformis*	KU257487	KU257514	–	KU308576	KU308608	Oshoro, Hokkaido, Japan	USNM 1106984
*Stenoscyphus inabai*	*Stenoscyphus inabai*	KU257488	KU257515	KU308636	KU308577	KU308609	Oshoro, Hokkaido, Japan	Photo voucher only
**Non identified species**								
*Depastromorpha* sp. AUS	*Depastromorpha* sp. AUS	KU257489	KU257516	KU308637	KU308578	KU308610	Outside Port Fairy, Abalone Farm, Victoria, Australia	MV F197278
*Kishinouyea* sp. Moorea	*Kishinouyea* sp. Moorea	KU257490	KU257517	KU308638	KU308579	KU308611	Moorea, French Polynesia	UF:Cnidaria:7226
*Kishinouyea* sp. NZ	*Kishinouyea* sp. NZ	KU257491	KU257518	KU308639	KU308580	KU308612	Taputeranga Marine Reserve, Wellington, New Zealand	NIWA 86808
*Kishinouyea* sp. SAF	*Kishinouyea* sp. SAF 1*	–	–	–	–	KU308613	Cape Town, South Africa	MZUSP 002731
	*Kishinouyea* sp. SAF 2*	KU257492	KU257519	KU308640	KU308581	–	Cape Town, South Africa	MZUSP 002732
*Lipkea* sp. JAP	*Lipkea* sp. JAP 1*			**			Aquarium, Katsuura, Chiba, Japan	USNM 1315325
	*Lipkea* sp. JAP 2*	KU257493	KU257520	–	KU308582	KU308614	Aquarium, Katsuura, Chiba, Japan	USNM 1315326
*Lucernaria* sp. EPR	*Lucernaria* sp. EPR	KU257494	DQ465037	KU308641	KU308583	KU308615	East Pacific Rise, 20 degrees south	USNM 1086349
*Lucernariopsis* sp. NZ	*Lucernariopsis* sp. NZ	KU257495	KU257521	KU308642	KU308584	KU308616	Taputeranga Marine Reserve, Wellington, New Zealand	NIWA 86809
*Stenoscyphus* sp. AUS	*Stenoscyphus* sp. AUS	KU257496	KU257522	KU308643	KU308585	KU308617	Williamstown, The Jawbone, Victoria, Australia	MV F190063

**Notes:**

* More than one specimen for each species was used to generate the combined alignment. ANT, Antarctica; AUS, Australia; EPR, East Pacific Rise; GER, Germany; JAP, Japan; NZ, New Zealand; SAF, South Africa; UK, the United Kingdom; USA, the United States of America; FMNH, Field Museum of Natural History, USA; KUNHM, University of Kansas Natural History Museum; MV, Museum Victoria, Australia; MZUSP, Museum of Zoology of the University of São Paulo; NIWA, National Institute of Water and Atmospheric Research; UF, University of Florida Museum of Natural History; USNM, National Museum of Natural History, Smithsonian, USA.

** Sequence with less than 200 nucleotides: ACGCCGTGCCAGGCCAAAATGTATTTTGTTACCTGCTGCACGGTGATGCTGAGCGCATTTTCTTTCTTCGTGGGCAAGAAAGAGGTGGTCGATAGATACGAGAGAGAGTGAGAGAGACGCGCGTCGTCCATCTCTCACTGACAATGACCTCAG.

**Table 2:** Primer sequences for polymerase chain reaction (PCR) and sequencing reaction.

Genes	Primers	Sequences	References
COI	jgHCO2198	TITCIACIAAYCAYAARGAYATTGG	Geller et al. (2013)
	jgLCO1490	TAIACYTCIGGRTGICCRAARAAYCA	Geller et al. (2013)
16S	F1mod	TCGACTGTTTACCAAAAACATA	Cunningham & Buss (1993) and Cartwright et al. (2008)
	R2	ACGGAATGAACTCAAATCATGTAAG	Cunningham & Buss (1993) and Cartwright et al. (2008)
	rnl_f_jl	GACTGTTTACCAAAGACATAGC	Designed by J. Lawley
	rnl_r_jl	AAGATAGAAACCTTCCTGTC	Designed by J. Lawley
ITS	jfITS1–5f	GGTTTCCGTAGGTGAACCTGCGGAAGGATC	Dawson & Jacobs (2001)
	CAS28SB1d	TTCTTTTCCTCCSCTTAYTRATATGCTTAA	Ji, Zhang & He (2003)
	C2	GAAAAGAACTTTGRARAGAGAGT	Chombard et al. (1997)
	D2	TCCGTGTTTCAAGACGGG	Chombard et al. (1997)
18S (SSU)	AF_cnidarian	GTGGYAATTCTAGAGCTAATACATGCG	Designed by R. Wilson
	BR_cnidarian	GCGACGGGCGGTGTGTAC	Designed by R. Wilson
	IF_cnidarian	GGGGGCATYCGTATTTCGTTG	Designed by R. Wilson
	IR_cnidarian	CAACGAAATACGRATGCCCCC	Designed by R. Wilson
	C_new cnidarian	CAGCCGCGGTAATTCCAGC	Designed by R. Wilson
	L_new cnidarian	CCTRTTCCATTATTCCATGCTC	Designed by R. Wilson
	O_new cnidarian	GGTCCAGACATAGTAAGGATTG	Designed by R. Wilson
	1800R18S	GTTCACCTACYGAAACCTTGTT	Redmond et al. (2007)
28S (LSU)	F63 mod	ACCCGCTGAAYTTAAGCATATHANTMAG	Medina et al. (2001)
	F63sq	AATAAGCGGAGGAAAAGAAAC	Medina et al. (2001)
	F97	CCYYAGTAACGGCGAGT	Evans et al. (2008)
	F635	CCGTCTTGAAACACGGACC	Medina et al. (2001)
	F1379sq	GACAGCAGGACGGTGGYCATGG	Medina et al. (2001)
	F1383	GGACGGTGGCCATGGAAGT	Collins et al. (2008) and Evans et al. (2008)
	F1586	GTGCAGATCTTGGTDGNAGTAGCAAATATTC	Medina et al. (2001)
	F1689	CTAAGMSRYAGGGAAAYTC	Collins et al. (2008)
	F2076sq	TAACYTCGGGAWAAGGATTGGCTC	Medina et al. (2001)
	F2766sq	AGTTTGGCTGGGGCGGYACA	Medina et al. (2001)
	F2800	GCAGGTGTCCTAAGGYRAGCTC	Voigt et al. (2004)
	R635sq	GGTCCGTGTTTCAAGACGG	Medina et al. (2001)
	R1411sq	GTTGTTACACACTCCTTAGCGG	Medina et al. (2001)
	R1630	CCYTTCYCCWCTCRGYCTTC	Medina et al. (2001)
	R2077sq	GAGCCAATCCTTWTCCCGARGTT	Medina et al. (2001)
	R2084	AGAGCCAATCCTTTTCC	Evans et al. (2008) and Collins et al. (2008)
	R2766sq	CAGRTGTRCCGCCCCAGCCAAACT	Medina et al. (2001)
	R2800	GAGCTYRCCTTAGGACACCTGC	Voigt et al. (2004)
	R3238	SWACAGATGGTAGCTTCG	Evans et al. (2008) and Collins et al. (2008)
	R3264	TTCYGACTTAGAGGCGTTCAG	Medina et al. (2001)

**Table 3:** Polymerase chain reaction (PCR) conditions for the different molecular markers used in the phylogenetic analyses.

Molecular marker	PCR condition
COI	94 °C: 5 min
	35 × −94 °C: 30 s; 50 °C: 40 s; 72 °C: 1 min
	72 °C: 7 min
	4 °C: forever
16S	95 °C: 5 min
	35 × −95 °C: 30 s; 45 °C: 50 s; 72 °C: 1 min
	72 °C: 5 min
	4 °C: forever
ITS	94 °C: 5 min
	35 × −94 °C: 30 s; 60 °C: 40 s; 72 °C: 1 min
	72 °C: 10 min
	4 °C: forever
18S (SSU)	94 °C: 5 min
	35 × −94 °C: 30 s; 57 °C: 30 s; 72 °C: 1 min
	72 °C: 7 min
	4 °C: forever
28S (LSU)	94 °C: 3 min
	35 × −95 °C: 30 s; 55 °C: 45 s; 72 °C: 1 min
	72 °C: 7 min
	4 °C: forever

**Table 4:** Sequences of the cnidarian outgroups used in the phylogenetic analyses of Staurozoa, including their GenBank accession numbers.

Class	Species	Molecular markers	
		18S (SSU)	28S (LSU)
Anthozoa	*Stichodactyla gigantea*	EU190873	EU190835
Cubozoa	*Carybdea rastonii*	AF358108	AY920787
	*Chironex fleckeri*	GQ849073	GQ849051
	*Tripedalia cystophora*	GQ849088	GQ849065
Hydrozoa	*Aglauropsis aeora*	AY920754	AY920793
	*Scrippsia pacifica*	AF358091	AY920804
Scyphozoa	*Atolla vanhoeffeni*	JX393273	AY026368
	*Chrysaora melanaster*	JX393281	AY920780
	*Phacellophora camtschatica*	JX393290	AY920778

Sequences were edited in SEQUENCHER™ 4.6 (Gene Codes Corporation) or GENEIOUS (Biomatters: available at http://www.geneious.com/), aligned using MAFFT (maxiterate 2.000, FFT-NS-i; Katoh & Standley, 2013), resulting in six alignments: individual COI, 16S, ITS, 18S, 28S, and a combined dataset (Table 5). Gblocks v.0.91b (Castresana, 2000; Talavera & Castresana, 2007) was run with standard parameters except that half the taxa were allowed to be gaps for any position. Gaps were treated as missing data. Parsimony analyses (PA) were performed with individual and combined dataset, using heuristic search (1,000 random addition replicates, with characters reweighted by maximum value of rescaled consistency indices) in PAUP* 4.1 (Swofford, 2002). The most appropriate model of nucleotide substitution for each dataset was chosen using jModelTest (Darriba et al., 2012), between 88 models, using default settings, and employing the Akaike information criterion (using AICc correction). The following models were used in the Maximum Likelihood and Bayesian analyses: COI–HKY+I+G; 16S–TIM2+I+G; ITS–K80+I+G; 18S–TIM2+I+G; 28S–TIM3+I+G; combined–GTR+I+G (no partitioned analyses were conducted). Maximum Likelihood analyses (ML) were performed with individual and combined dataset, using PhyML 3.0 (Guindon et al., 2010). Branch support was estimated by bootstrapping (Felsenstein, 1985) with 1,000 replicates for the PA (PAUP* 4.1) and ML (PhyML) analyses. The Bayesian inference (BA) was also performed with individual and combined dataset, in MrBayes v3.2 (Ronquist & Huelsenbeck, 2003), with 5,000,000 generations sampled every 1,000 generations, four chains, and four independent runs. One fourth of the topologies were discarded as burnin, and the remaining used to calculate the posterior probability. Following MrBayes v3.2 manual, convergence was assessed by ensuring that the average standard deviation of split frequencies was less than 0.01 after 5,000,000 generations, and that the convergence statistic (PSRF = Potential Scale Reduction Factor) was close to 1.0 for all parameters. FigTree (http://tree.bio.ed.ac.uk/software/figtree/) was used to visualize and edit the resulting trees. The alignments and trees are available in the repository of phylogenetic information TreeBASE at: http://purl.org/phylo/treebase/phylows/study/TB2:S18971.

**Table 5:** Molecular alignments information.

Alignments	NT	SA	C	V	Pi	S
COI	27	587	344	243	232	11
16S	35	561	301	260	239	21
ITS	32	314	206	108	60	48
18S (SSU)	42	1,562	1,275	287	198	89
28S (LSU)	42	3,004	2,285	719	558	161
Combined	45	6,028	4,411	1,617	1,287	330

**Note:**

NT, number of taxa; SA, size of alignment; C, conserved sites; V, variable sites; Pi, parsimony informative sites; S, singleton sites.

Selected morphological characters generally used in the taxonomy of Staurozoa were optimized by using ACCTRAN (accelerated transformation) in the combined molecular phylogenetic tree at the generic level, using TNT 1.1 (Goloboff, Farris & Nixon, 2008).

### Morphology

Detailed images of morphological structures from specimens (Table 6) fixed in 4% formaldehyde solution with seawater were photographed under the stereomicroscope SteREO Discovery.V8, Zeiss (Germany). Histological procedures were carried out according to the methods developed for Staurozoa (Miranda, Collins & Marques, 2013; modified from Humanson (1962) and Mahoney (1966)). Specimens were cleaned in distilled water; dehydrated in a graded ethanol series (70–100%); cleared in xylene (three steps); infiltrated and embedded in paraffin; serially sectioned transversely (7.0–10.0 μm thick) with a microtome Leica RM2025; cleared in xylene (twice); rehydrated in a graded ethanol series (100-70%); cleaned in distilled water; and stained, using acid fuchsin (15′) (Mallory; Humanson, 1962: 147) and acetic aniline blue (3′) (Mallory; modified from Humanson (1962: 231)), intercalated with distilled water to improve the contrast between structures. Prepared slides were observed and photographed under a microscope Axio Imager M2, Carl Zeiss (Germany).

**Table 6:** Species of Staurozoa used in the detailed morphological descriptions, with respective localities, voucher catalog numbers, and slides catalog numbers.

Species	Locality	Voucher catalog number	Slides catalog number
*Craterolophus convolvulus*	Woods Hole, Massachusetts, USA	USNM 54321	LEM 17
*Depastromorpha africana*	Kalk Bay, Cape Town, South Africa	MZUSP 002733	–
*Haliclystus tenuis*	Muroran, Hokkaido, Japan	USNM 1106652	LEM 09
*Kishinouyea corbini*	Aracruz, Espírito Santo, Brazil	MZUSP 1563	LEM 14
*Kishinouyea* sp. NZ	Taputeranga Marine Reserve, Wellington, New Zealand	NIWA 86808	LEM 18
*Lipkea* sp. Japan	Aquarium, Katsuura, Chiba, Japan	USNM 1315325	–
*Lucernariopsis campanulata*	Île Verte, Roscoff, France	USNM 1233741	–
*Lucernariopsis cruxmelitensis*	Wembury, Plymouth, England	USNM 1233742	–
*Lucernariopsis tasmaniensis*	Gerloff Bay, South Australia, Australia	USNM 1233740	–
*Lucernariopsis vanhoeffeni*	Janus Island, Palmer Archipelago, Antarctica	USNM 79939	–
*Manania uchidai*	Muroran, Hokkaido, Japan	USNM 1106645	LEM 10
*Sasakiella cruciformis*	Muroran, Hokkaido, Japan	USNM 1106656	LEM 15

**Note:**

LEM, Laboratory of Marine Evolution of the Institute of Biosciences, University of São Paulo; MZUSP, Museum of Zoology of the University of São Paulo; NZ, New Zealand; NIWA, National Institute of Water and Atmospheric Research; USNM, National Museum of Natural History, Smithsonian, USA.

## Results and Discussion

### Phylogeny

**Figure 3:**
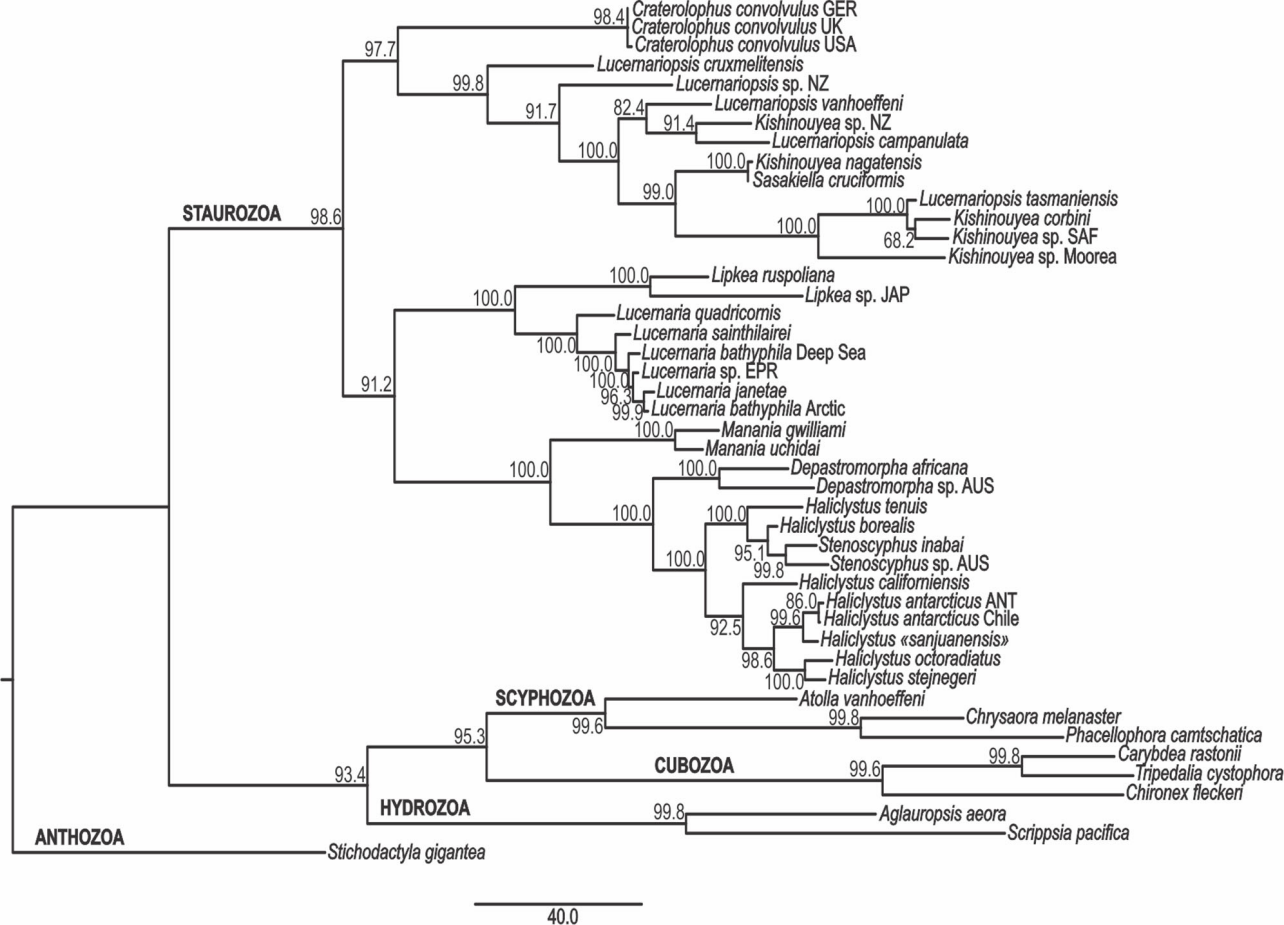
Parsimony phylogenetic hypothesis. Analysis based on combined data of mitochondrial markers COI and 16S, and nuclear markers ITS, 18S (SSU), and 28S (LSU). Single most parsimonious tree, length: 1682.18 steps. Bootstrap indices under parsimony at each node. ANT, Antarctica; AUS, Australia; EPR, East Pacific Rise; GER, Germany; JAP, Japan; NZ, New Zealand; SAF, South Africa; UK, the United Kingdom; USA, the United States of America.

**Figure 4:**
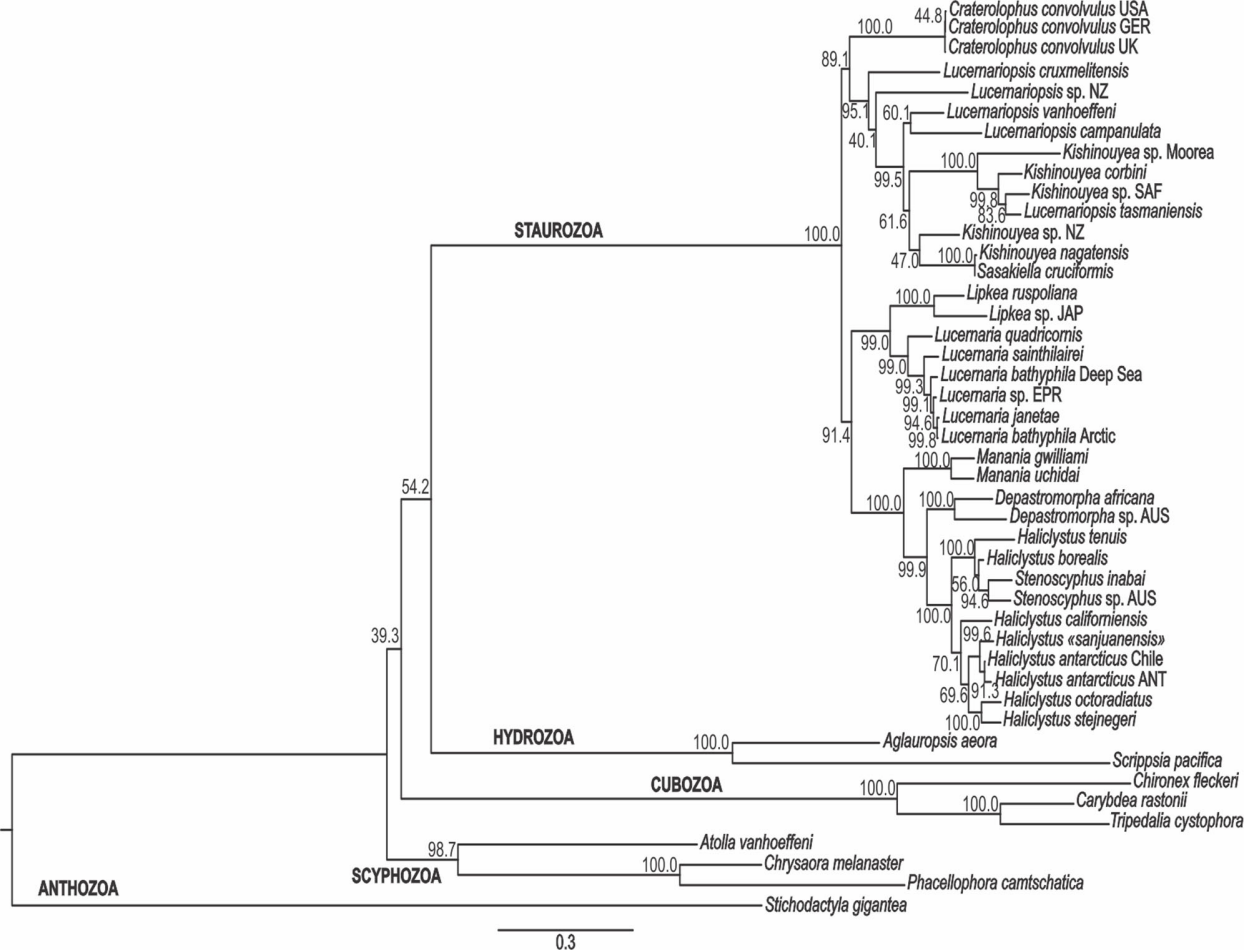
Maximum likelihood phylogenetic hypothesis. Analysis based on combined data of mitochondrial markers COI and 16S, and nuclear markers ITS, 18S (SSU), and 28S (LSU). Bootstrap indices under maximum likelihood at each node. ANT, Antarctica; AUS, Australia; EPR, East Pacific Rise; GER, Germany; JAP, Japan; NZ, New Zealand; SAF, South Africa; UK, the United Kingdom; USA, the United States of America.

**Figure 5:**
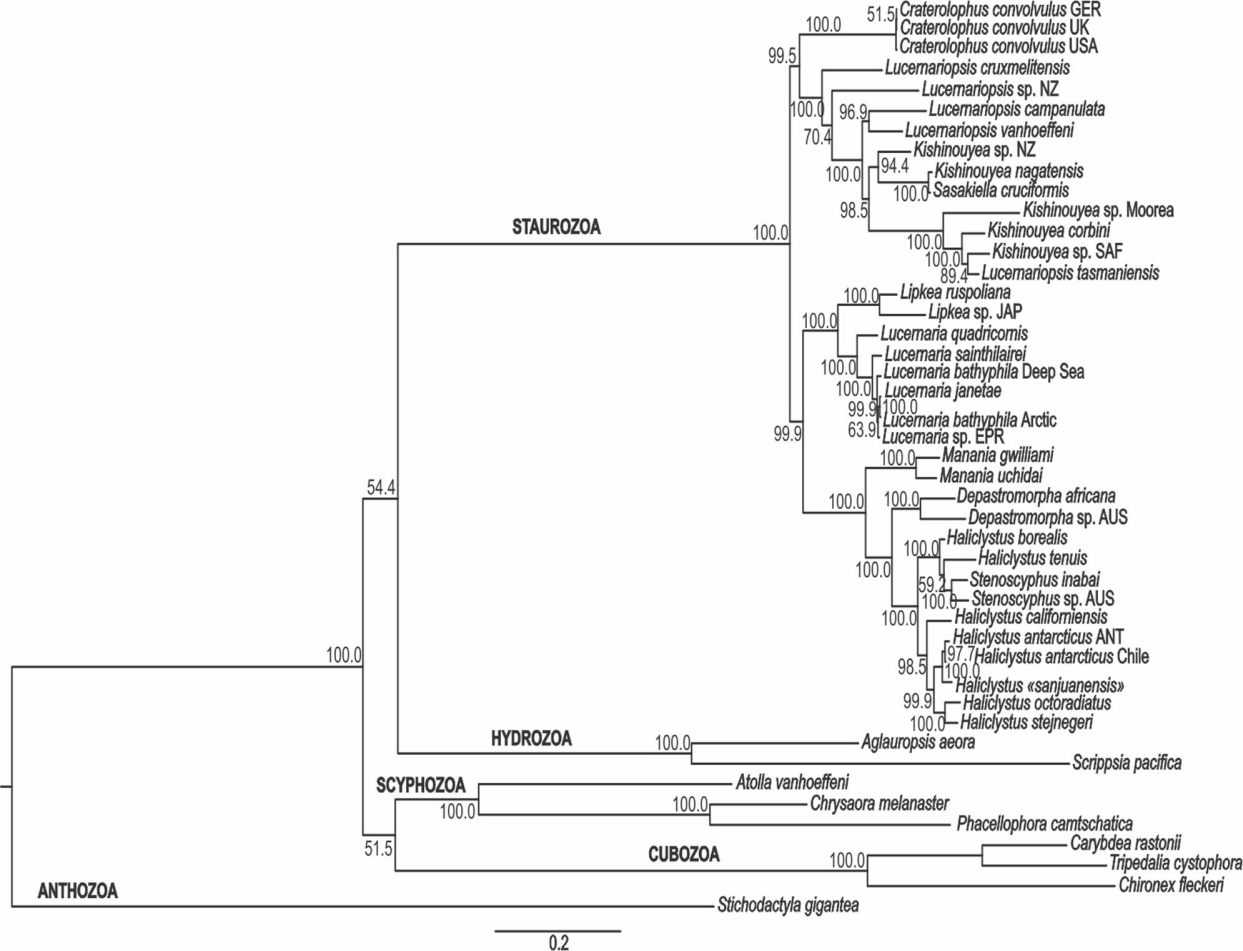
Bayesian phylogenetic hypothesis. Analysis based on combined data of mitochondrial markers COI and 16S, and nuclear markers ITS, 18S (SSU), and 28S (LSU). Posterior probability at each node. ANT, Antarctica; AUS, Australia; EPR, East Pacific Rise; GER, Germany; JAP, Japan; NZ, New Zealand; SAF, South Africa; UK, the United Kingdom; USA, the United States of America.

**Figure 6:**
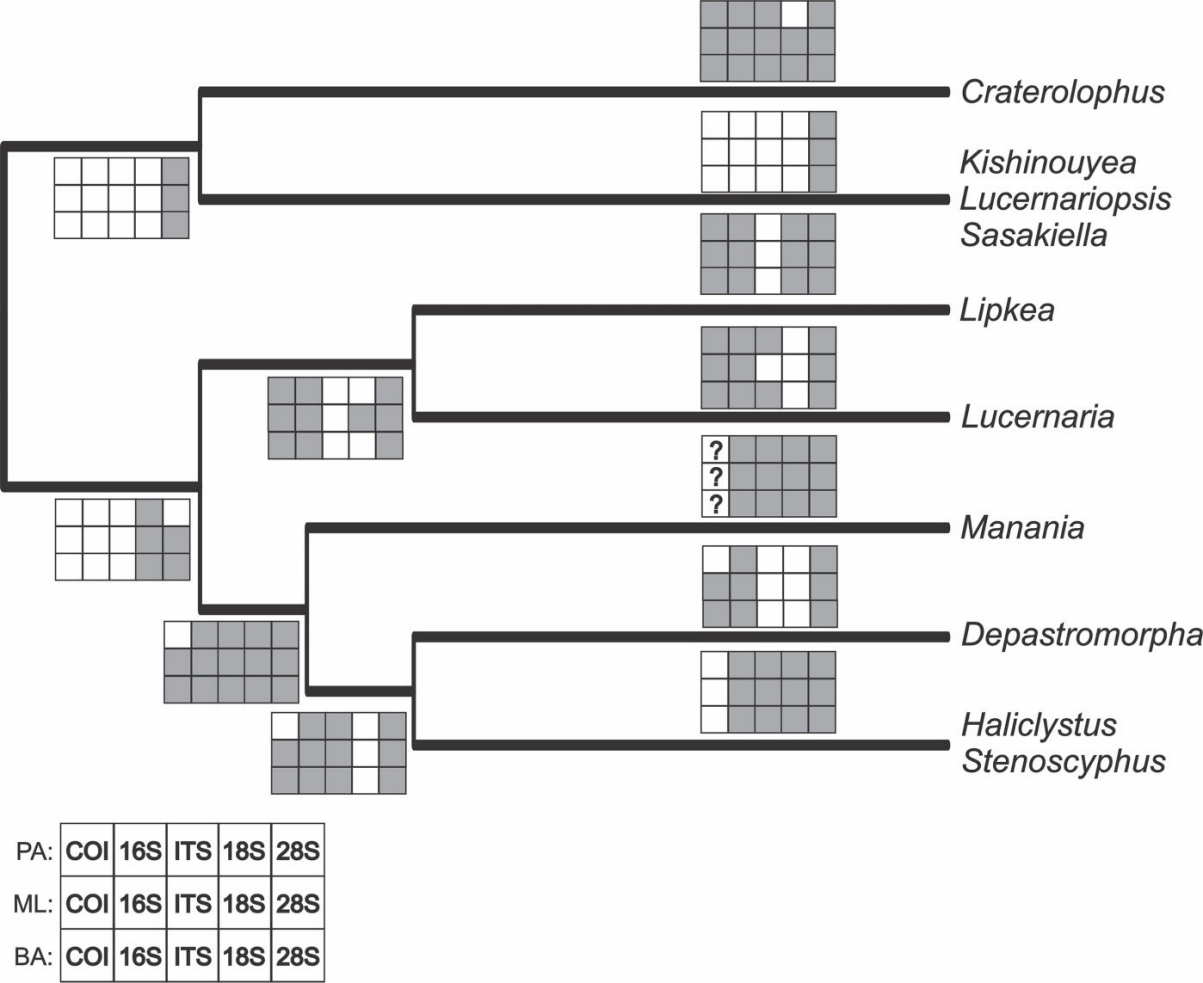
Support of each individual molecular marker for the main groups observed in the combined analyses. White squares represent non monophyletic groups, and gray squares represent monophyletic groups. First row: individual molecular markers under parsimony analyses; second row: individual molecular markers under maximum likelihood analyses; third row: individual molecular markers under Bayesian analyses. PA, parsimony; ML, maximum likelihood; BA, Bayesian. “?” indicates groups whose monophyly could not be corroborate for a particular molecular marker (only one species).

**Figure 7:**
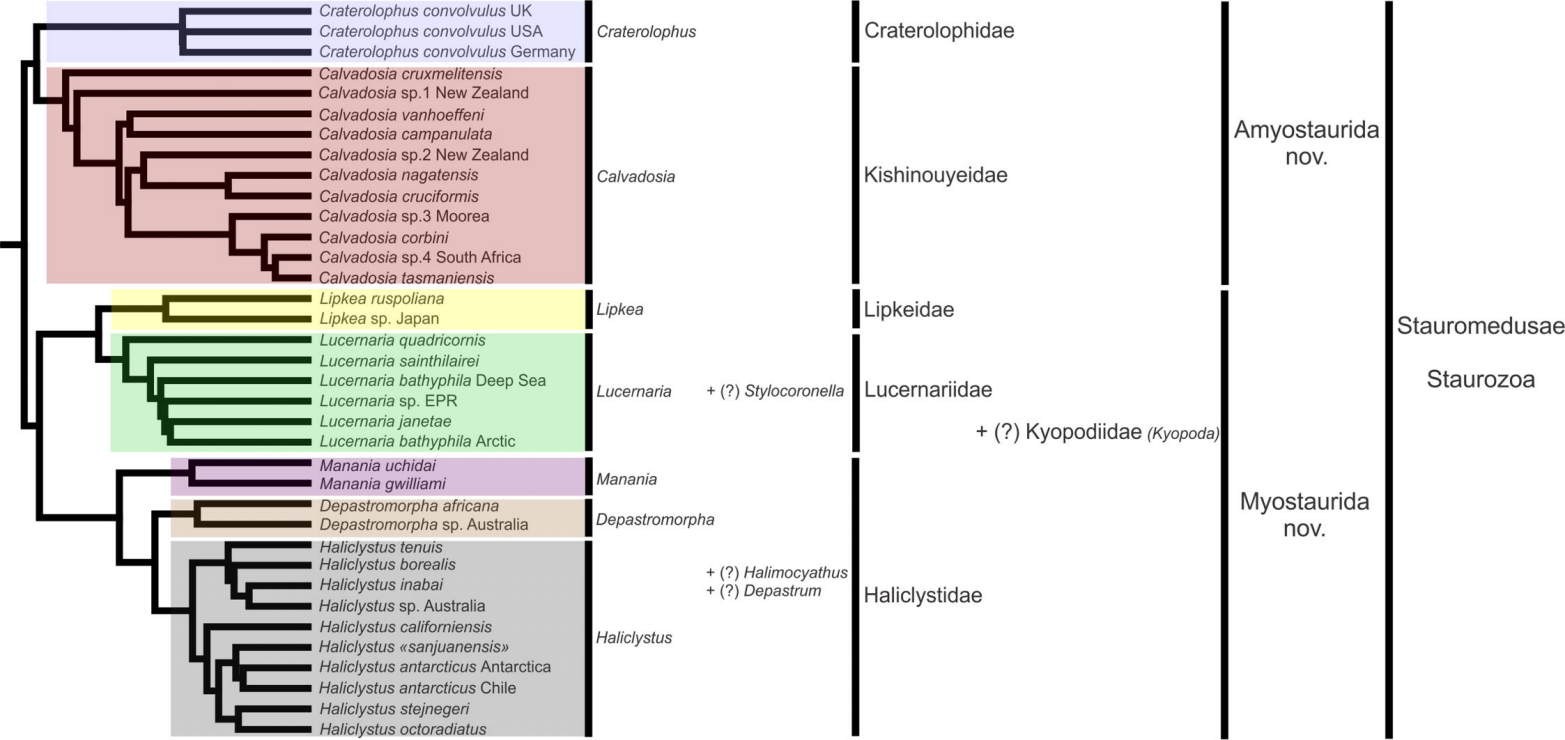
New proposal of classification based on molecular phylogenetic analyses. “?” indicates groups not included in the analysis, classified according to morphological evidence. EPR, East Pacific Rise; UK, the United Kingdom; USA, the United States of America.

**Figure 8:**
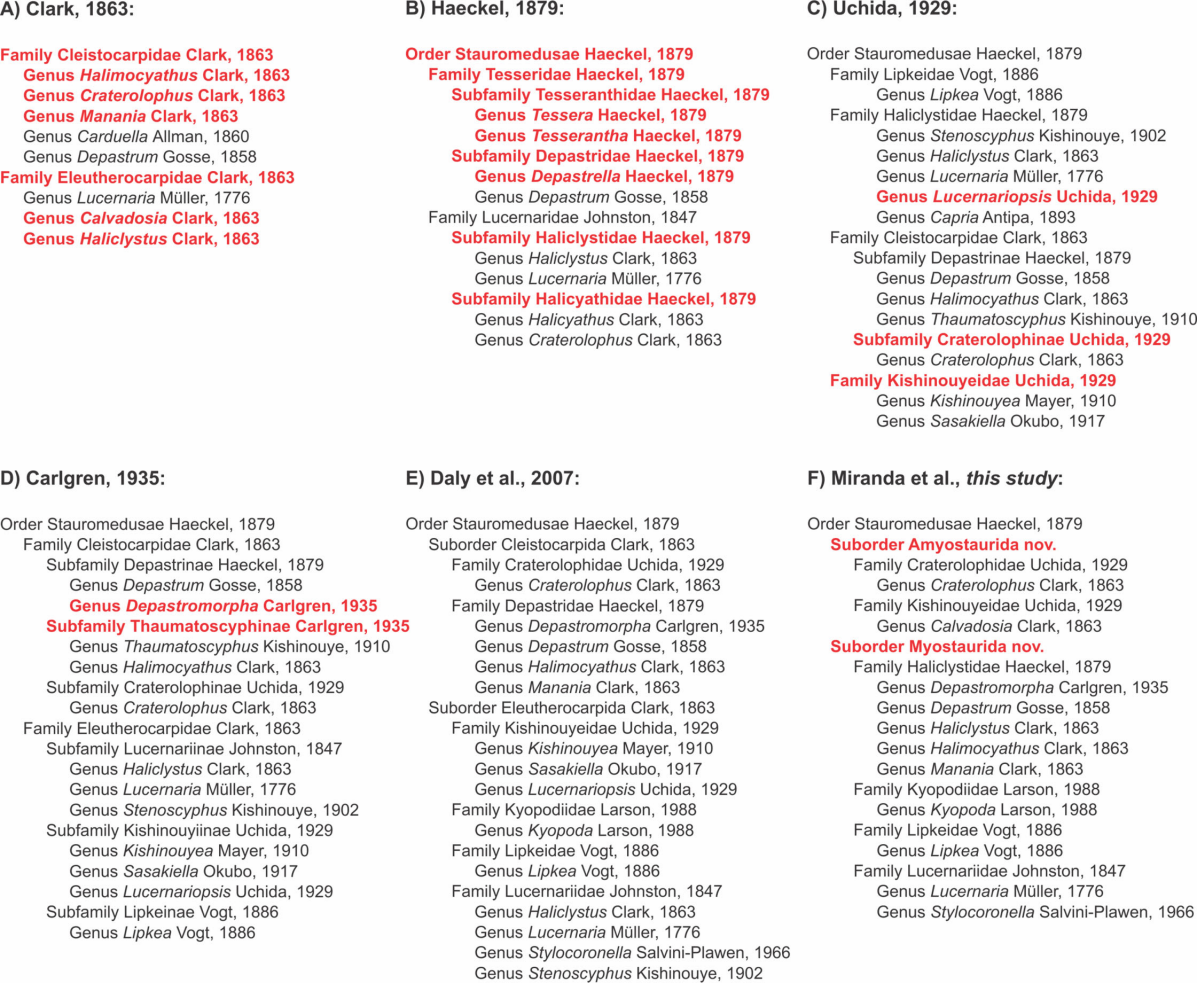
Historical proposals of classifications for Staurozoa. Classification proposed in this study (F), based on molecular phylogenetic analysis and on additional morphological evidence. In red, new names proposed by the author of respective classification.

The PA, ML, and BA topologies based on combined markers are similar (Figs. 3–5). The main difference is the relationships among *Lucernariopsis vanhoeffeni*, *Lucernariopsis campanulata*, and *Kishinouyea* sp. NZ (Figs. 3–5) and the relationships among *Kishinouyea corbini*, *Lucernariopsis tasmaniensis*, and *Kishinouyea* sp. SAF. Single-gene topologies under PA, ML, and BA show varying levels of correspondence to the combined topology (Figs. S1–S15 and 6). At least one molecular marker individually supports each main group observed in the PA, ML, and BA results (Fig. 6). This is the most comprehensive molecular phylogenetic hypothesis that has been presented for Staurozoa, which consequently allows us to carry out a comparative analysis of trait distribution across clades, as well as to provide a major revision for the classification of the class (Figs. 7 and 8; Table 7).

**Table 7:** New proposal for classification of Staurozoa based on the phylogenetic hypotheses (Figs. 3–5 and 7), also considering non-sampled genera (see text for further explanation).

Upper Rank			Family	Genus	Species
CLASS STAUROZOA Marques & Collins, 2004	Order Stauromedusae Haeckel, 1879	Suborder Amyostaurida nov.	Craterolophidae Uchida, 1929	*Craterolophus* Clark, 1863	*C. convolvulus* (Johnston, 1835)*
					*C. macrocystis* von Lendenfeld, 1884
			Kishinouyeidae Uchida, 1929	*Calvadosia* Clark, 1863	*C. campanulata* (Lamouroux, 1815)*
					*C. capensis* (Carlgren, 1938)
					*C. corbini* (Larson, 1980)
					*C. cruciformis* (Okubo, 1917)
					*C. cruxmelitensis* (Corbin, 1978)
					*C. hawaiiensis* (Edmondson, 1930)
					*C. nagatensis* (Oka, 1897)
					*C. tsingtaoensis* (Ling, 1937)
					*C. tasmaniensis* (Zagal et al., 2011)
					*C. vanhoeffeni* (Browne, 1910)
		Suborder Myostaurida nov.	Haliclystidae Haeckel, 1879	*Depastromorpha* Carlgren, 1935	*D. africana* Carlgren, 1935*
				*Depastrum* Gosse, 1858	*D. cyathiforme* (Sars, 1846)*
				*Haliclystus* Clark, 1863	*H. antarcticus* Pfeffer, 1889
					*H. auricula* Clark, 1863*
					*H. borealis* Uchida, 1933
					*H. californiensis* Kahn et al., 2010
					*H. inabai* (Kishinouye, 1893)
					*H. kerguelensis* Vanhöffen, 1908
					*H. monstrosus* (Naumov, 1961)
					*H. octoradiatus* Clark, 1863
					*H. salpinx* Clark, 1863
					*H. “sanjuanensis” nomen nudum*
					*H. sinensis* Ling, 1937
					*H. stejnegeri* Kishinouye, 1899
					*H. tenuis* Kishinouye, 1910
				*Halimocyathus* Clark, 1863	*H. platypus* Clark, 1863*
				*Manania* Clark, 1863	*M. atlantica* (Berrill, 1962)
					*M. auricula* (Fabricius, 1780)*
					*M. distincta* (Kishinouye, 1910)
					*M. gwilliami* Larson & Fautin, 1989
					*M. handi* Larson & Fautin, 1989
					*M. hexaradiata* (Broch, 1907)
					*M. uchidai* (Naumov, 1961)
			Kyopodiidae Larson, 1988	*Kyopoda* Larson, 1988	*K. lamberti* Larson, 1988*
			Lipkeidae Vogt, 1886	*Lipkea* Vogt, 1886	*L. ruspoliana* Vogt, 1886*
					*L. stephensoni* Carlgren, 1933
					*L. sturdzii* (Antipa, 1893)
			Lucernariidae Johnston, 1847	*Lucernaria* Müller, 1776	*L. australis* Vanhöffen, 1908
					*L. bathyphila* Haeckel, 1879
					*L. haeckeli* (Antipa, 1892)
					*L. infundibulum* Haeckel, 1879
					*L. janetae* Collins & Daly, 2005
					*L. quadricornis* Müller, 1776*
					*L. sainthilairei* (Redikorzev, 1925)
					*L. walteri* (Antipa, 1892)
				*Stylocoronella* Salvini-Plawen, 1966	*S. riedli* Salvini-Plawen, 1966*
					*S. variabilis* Salvini-Plawen, 1987

**Note:**

* Type species for each genus.

### Higher level systematics

#### Suborders Amyostaurida nov. and Myostaurida nov.

**Figure 9:**
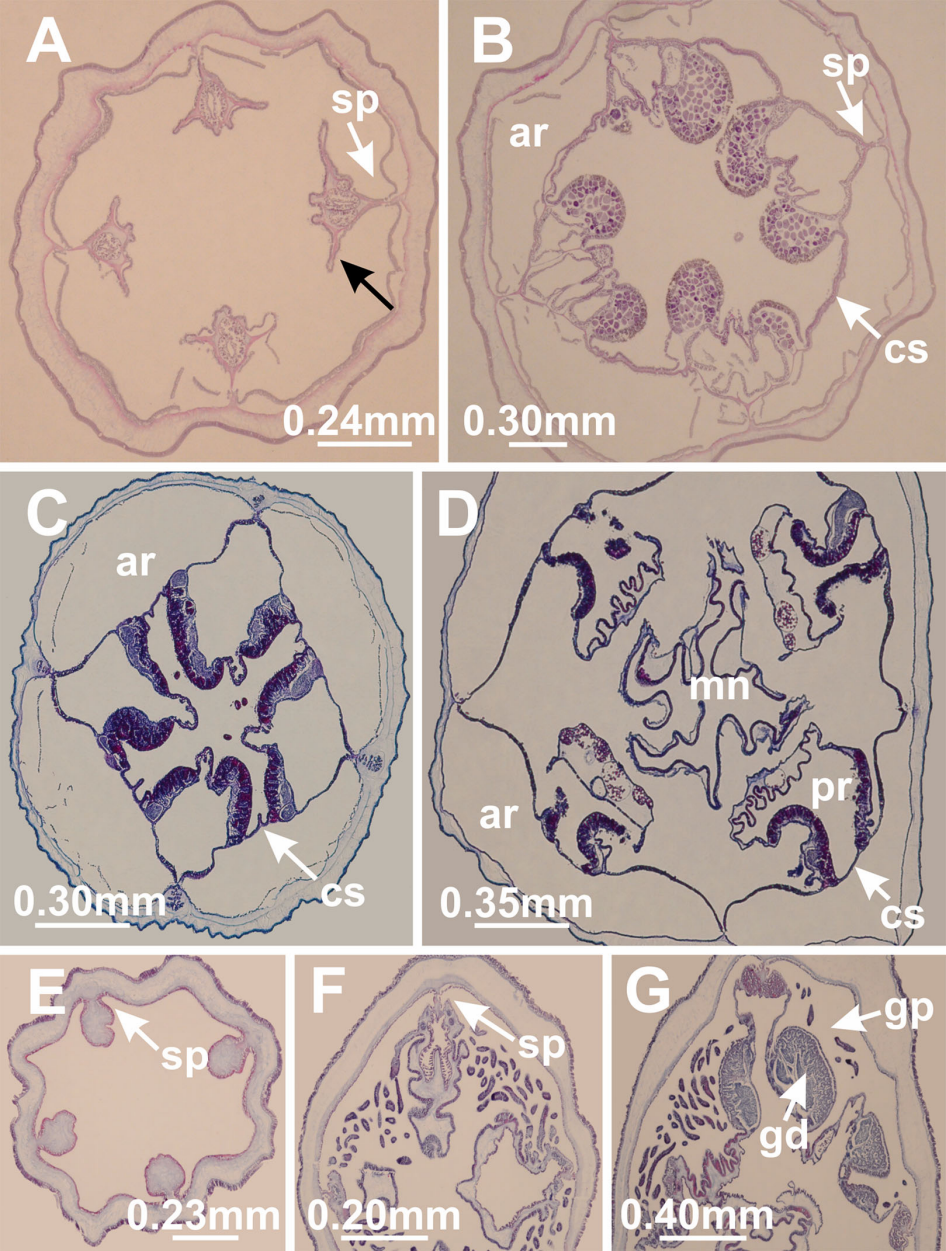
Claustrum connecting adjacent septa. *Craterolophus convolvulus*: (A) beginning of claustrum delimitation (indicated by black arrow) between adjacent septa (sp) in peduncle; (B) claustrum (cs) completely delimited at base of calyx, enclosing accessory radial pockets (ar); *Manania uchidai*: (C) claustrum (cs) completely delimited at base of calyx, enclosing accessory radial pockets (ar); (D) claustrum (cs) between accessory radial pockets (ar) and principal radial pockets (pr) (associated with gonads) in calyx, and a central manubrium (mn); *Calvadosia* sp. 2 NZ: (E) absence of claustrum connecting adjacent septa (sp) in peduncle; (F) absence of claustrum at the base of calyx between adjacent septa (sp); (G) gastric radial pocket (gp) associated with gonads (gd). Cross-sections. Photo credit: Lucília Miranda.

**Figure 10:**
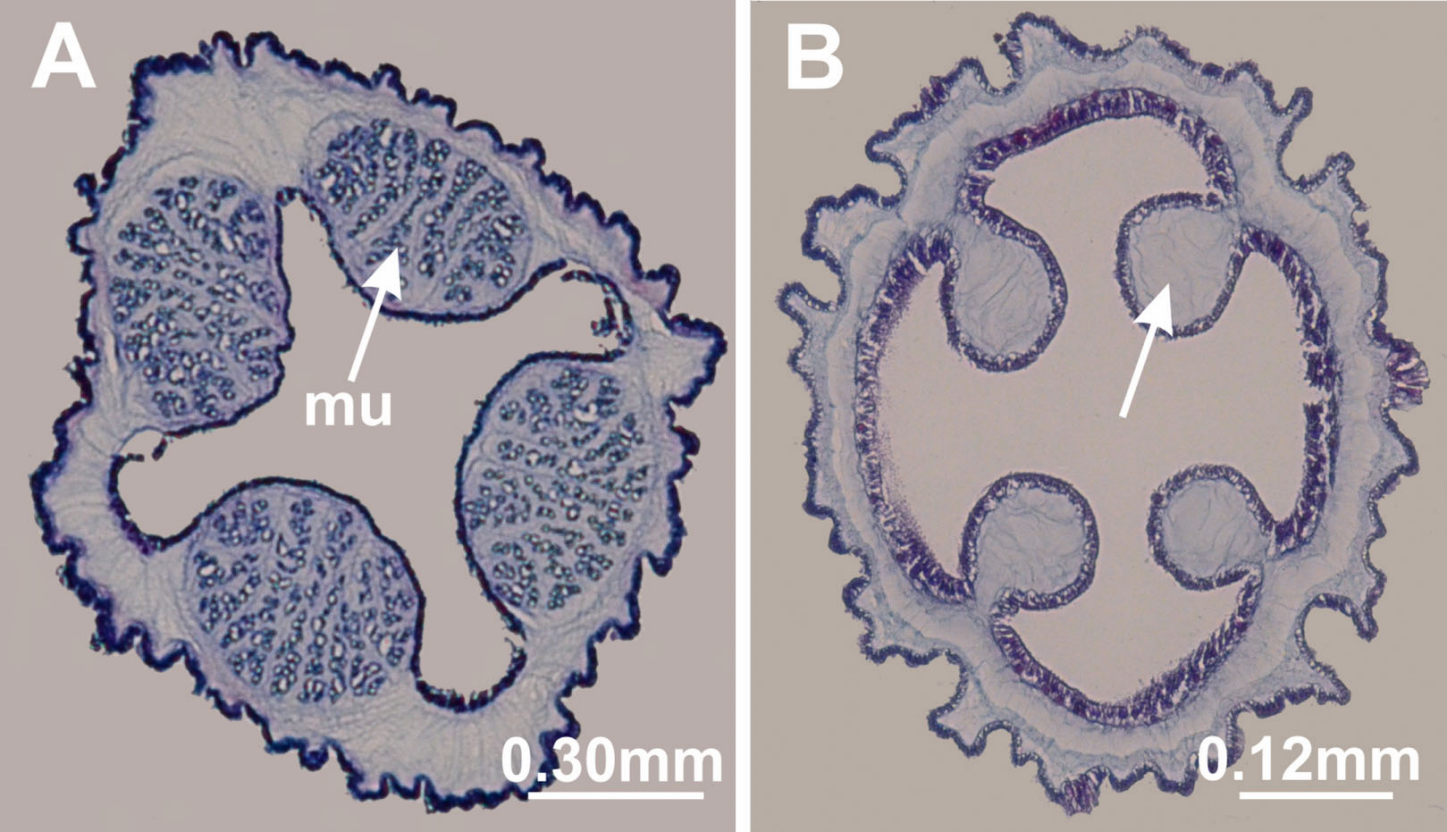
Interradial longitudinal muscles in peduncle. *Manania uchidai*: (A) presence of interradial longitudinal muscles (mu); *Calvadosia cruciformis*: (B) absence of interradial longitudinal muscles (indicated by arrow). Cross-sections. Photo credit: Lucília Miranda.

**Figure 11:**
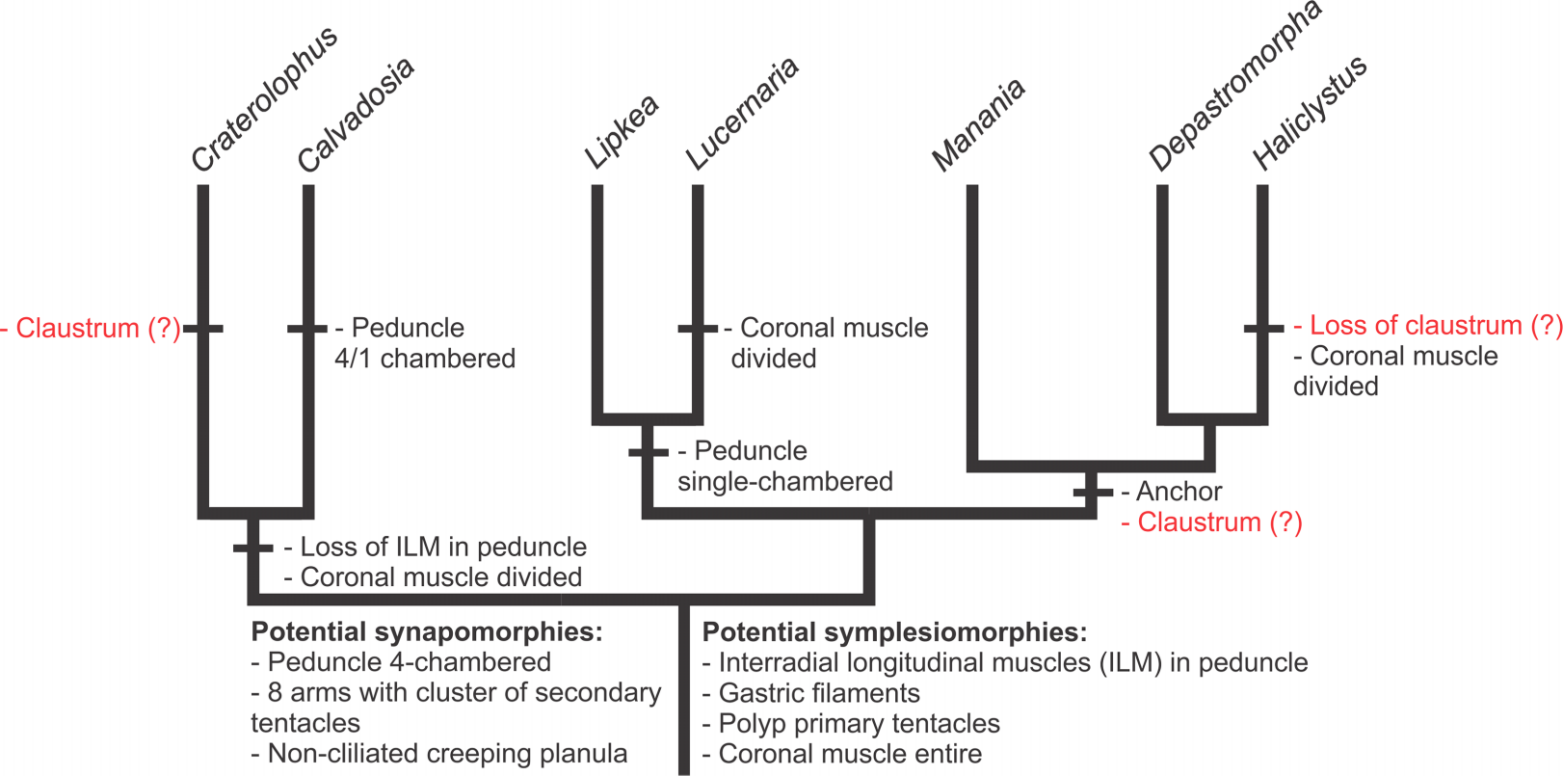
Hypothesis of character evolution for staurozoan genera. ACCTRAN optimization of selected morphological and life-history features according to our molecular phylogenetic analyses. Synapomorphies and symplesiomorphies are based on Collins & Daly (2005). The presence of claustrum as a potential symplesiomorphy of Staurozoa (Collins & Daly, 2005) is equivocal, and the state in outgroups needs careful reconsideration based on detailed histological studies. If considered a symplesiomorphy of Staurozoa, claustrum was lost in *Calvadosia*, *Haliclystus*, and in the clade *Lucernaria* + *Lipkea* (most parsimonious reconstruction). Anchors are adhesive structures resulting from metamorphosis of eight primary tentacles (perradial and interradial). Coronal muscle divided into eight sections by the adradial arms or entire. The species with 4/1-chambered peduncle have four chambers basally and one chamber in the middle of the peduncle.

The class Staurozoa has traditionally been divided into the subgroups Cleistocarpida and Eleutherocarpida (Fig. 8), based on the presence and absence, respectively, of an internal structure called the claustrum (Fig. 9; Table 8). However, a preliminary phylogenetic analysis for the class (Collins & Daly, 2005) suggested that these groups, proposed by Clark (1863) (Fig. 8), were not monophyletic. Our study, with better taxon sampling, corroborates this preliminary result, and refutes the suborders Eleutherocarpida and Cleistocarpida (Fig. 8). Instead, our working hypothesis based on our combined set of molecular data (Fig. 7) shows that staurozoans are divided into two well-supported groups, which can be distinguished one from the other by the absence/presence of interradial longitudinal muscles in the peduncle (or stalk) (Figs. 10 and 11; Table 8). We propose two new suborders for the Staurozoa: Myostaurida (from the Greek *myos*: muscle; *stauro*: cross) and Amyostaurida composed of species with and without interradial muscles in the peduncle, respectively (Figs. 7, 8F, 10 and 11; Table 8). Presence of longitudinal muscles in the peduncle (Figs. 10A and 11) is a character easily recognizable with a cross-section of the middle region of the peduncle (Uchida, 1929; Ling, 1937; Ling, 1939; Berrill, 1963; Miranda, Collins & Marques, 2013), and consequently a useful feature for distinguishing the two major subgroups of stalked jellyfishes (see discussion about character evolution below).

**Table 8:** Main morphological characters used in the taxonomy of Staurozoa and their occurrence in each genus.

		Staurozoan genera										
Characters	States	*Craterolophus*	*Calvadosia*	*Depastromorpha*	*Depastrum*	*Haliclystus*	*Halimocyathus*	*Manania*	*Kyopoda*	*Lipkea*	*Lucernaria*	*Stylocoronella*
Claustrum	Present	X		X	X		X	X				
	Absent		X			X			X	X	X	X
Muscles in peduncle	Present			X	X	X	X	X	X	X	X	X
	Absent	X	X									
Number of chambers in peduncle	4	X		X	X	X	X	X	?			
	1		X					X		X	X	X
	4 basal, 1 medial		X									
	1 basal, 4 medial							X				
Anchors and primary tentacles	Absent	X	X							?	X	
	Primary tentacles		X		X							Migration (see text)
	Anchors			X		X	X	X	X			
Pad-like adhesive structures	Absent				X	X				X	X	X
	Tentacles	X	X	X		X	X	X	X			
	Arms		X									
Coronal muscle	Divided	X	X			X					X	
	Entire			X	X	X	?	X	X	X		
	Vestigial											X

**Note:**

Based on Gosse (1860), Clark (1863), Mayer (1910), Uchida (1929), Uchida & Hanaoka (1933), Uchida & Hanaoka (1934), Carlgren (1935), Ling (1937), Kramp (1961), Larson (1980), Larson (1988), Hirano (1986), Hirano (1997), Larson & Fautin (1989), Kikinger & Salvini-Plawen (1995) and Pisani et al. (2007).

#### Family Craterolophidae Uchida, 1929

*Type genus*: *Craterolophus* Johnston, 1835

Craterolophinae was proposed by Uchida (1929) (Fig. 8C) as a subfamily of Cleistocarpidae, defined as stauromedusae with claustrum and without longitudinal interradial muscles in the peduncle (Figs. 9 and 10; Table 8). This classification was followed by Carlgren (1935) (Fig. 8D). The subfamily is monogeneric and contains only two valid species: *Craterolophus convolvulus* (Johnston, 1835) and *Craterolophus macrocystis* von Lendenfeld, 1884.

We followed Daly et al. (2007) and elevated Craterolophinae to the family level, as Craterolophidae (Figs. 7, 8E and 8F), including only the genus *Craterolophus* (Figs. 7 and 8; Table 7). We included specimens of *C. convolvulus* from Europe (Germany and the United Kingdom) and from the U.S.A. (Table 1) in our analysis. However, there was no specimen available of *C. macrocystis*; the species is very rare, having been recorded only twice (Hutton, 1880; von Lendenfeld, 1884). Therefore, the monophyly of the genus and, consequently, the family, remains to be tested.

#### Family Kishinouyeidae Uchida, 1929

*Type genus*: *Calvadosia* Clark, 1863

The family Kishinouyeidae was proposed by Uchida (1929) to include the genera *Kishinouyea* and *Sasakiella* (Fig. 8C). Carlgren (1935) proposed an amendment to also include the genus *Lucernariopsis* (Fig. 8D).

The monophyly of the family was tested and corroborated in our analysis (Figs. 3–5). However, the two traditional genera *Lucernariopsis* and *Kishinouyea* did not resolve as monophyletic (Figs. 3–5). According to current taxonomy, the distinction between the three genera of this family is subtle. *Kishinouyea* and *Sasakiella* differ by the absence and presence, respectively, of primary tentacles (Ling, 1937). Both *Kishinouyea* and *Lucernariopsis* do not have primary tentacles in adults, but they are thought to differ in the internal anatomy of the peduncle. Whereas species of *Kishinouyea* (and *Sasakiella*) have four chambers basally and one chamber in the middle of the peduncle, species of *Lucernariopsis* have just one chamber throughout the peduncle (Uchida, 1929; Kramp, 1961). However, these characters change during development (Uchida, 1929; Hirano, 1986). Additionally, a cross-section at the very base of the peduncle is rarely reported in the description of species; most only include information concerning the middle region of the peduncle (e.g., *Kishinouyea hawaiiensis* in Edmondson, 1930; *Lucernariopsis capensis* in Carlgren, 1938; Miranda et al., 2012), or do not mention where the peduncle was sectioned (e.g., Corbin, 1978), causing some doubt about whether this distinction is reliable in defining these genera. Recently, *Lucernariopsis tasmaniensis* was described with “a single cruciform chamber that becomes four-chambered basally within pedal disc” (Zagal et al., 2011), a character that corresponds to the genera *Kishinouyea* and *Sasakiella* (Kramp, 1961). Not surprisingly, our phylogenetic hypothesis (Figs. 3–5) indicates that the traditional distinctions between these genera are not robust.

We suggest that the three genera of Kishinouyeidae be synonymized due to the lack of characters to differentiate them. *Kishinouyea* Mayer, 1910 would have priority over *Lucernariopsis* Uchida, 1929 and *Sasakiella* Okubo, 1917. However, there is a further nomenclatural problem in Uchida’s (1929) proposal of the genus *Lucernariopsis* based on *Lucernaria campanulata* (Lamouroux, 1815; Gwilliam, 1956: 10). Previously, Clark (1863) had recognized Lamouroux’ species as not assignable to *Lucernaria*, since the species does not have interradial muscles in the peduncle, and proposed the new genus name *Calvadosia* (*non Calvadosia* Cossmann 1921; junior synonym of *Calvadosiella* Wenz 1939; Mollusca, Gastropoda) to accommodate it. Thus, following the rule of priority, the proper generic name of *Lucernariopsis* Uchida, 1929 would be *Calvadosia* Clark, 1863. Consequently, *Calvadosia* has priority over *Kishinouyea* Mayer, 1910, and we therefore synonymize *Kishinouyea*, *Sasakiella*, and *Lucernariopsis* within *Calvadosia*. The name of the family remains the same, according to ICZN, article 40.1.

#### Family Haliclystidae Haeckel, 1879

*Type genus*: *Haliclystus* Clark, 1863

Haliclystidae was proposed by Haeckel (1879) as a subfamily of Lucernaridae, including the genera *Haliclystus* and *Lucernaria* (Fig. 8B). Uchida (1929) raised Haliclystidae to the family level, adding the genera *Stenoscyphus*, *Capria* (currently regarded as congeneric with *Lipkea*), and *Lucernariopsis* (Fig. 8C). The family was dismissed by Carlgren (1935), who divided the genera of “Haliclystidae” into three subfamilies of Eleutherocarpidae: Lucernariinae (*Haliclystus*, *Stenoscyphus*, and *Lucernaria*), Lipkeinae (*Lipkea*), and Kishinouyiinae (*Lucernariopsis*) (Fig. 8D).

Our phylogenetic analyses show a close relationship between *Haliclystus*, *Stenoscyphus*, *Depastromorpha*, and *Manania* (Figs. 3–5). Based on this evidence and on morphological similarities (see below, Table 8), we propose that these genera should be assigned to the family Haliclystidae (Figs. 7 and 8F; Table 7). We also include in this family the not yet sampled genera *Depastrum* and *Halimocyathus*, but this needs to be tested in future studies.

According to the phylogeny, *Stenoscyphus inabai* is closely related to *Haliclystus borealis* and *Haliclystus tenuis* (Figs. 3–5), and deeply nested within *Haliclystus* spp. In order to keep *Haliclystus* monophyletic, and since the name *Haliclystus* Clark, 1863 has priority over the name *Stenoscyphus* (a monospecific genus) Kishinouye, 1902, we synonymize *Stenoscyphus* with *Haliclystus* (Figs. 7 and 8F; Table 7). Some limited developmental data has already suggested a close relationship between these two genera (Hirano, 1986). The main difference between the former genus *Stenoscyphus* and *Haliclystus* is an entire and divided coronal muscle, respectively (Kramp, 1961; Hirano, 1986). Therefore, *Haliclystus inabai* is the only described *Haliclystus* with an entire coronal muscle (Table 8).

**Figure 12:**
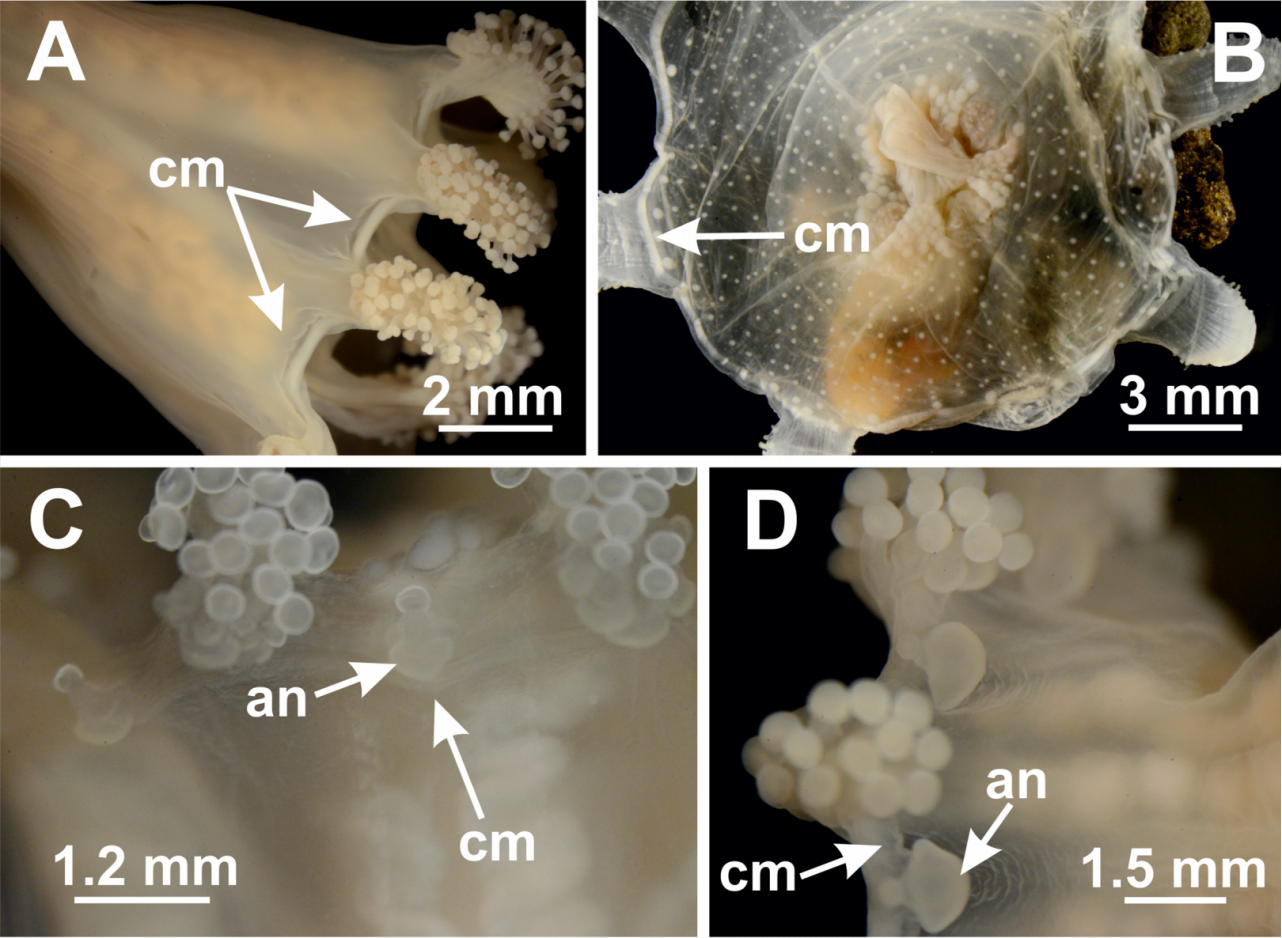
Coronal muscle. *Craterolophus convolvulus*: (A) divided coronal muscle (cm); *Lipkea* sp. Japan: (B) entire coronal muscle (cm); *Manania uchidai*: (C) external (exumbrellar) coronal muscle (cm) in relation to anchor (an); *Depastromorpha africana*: (D) internal (subumbrellar) coronal muscle in relation to anchor (an). Photo credit: Lucília Miranda.

Genetic data suggest that *Depastromorpha* is more closely related to *Haliclystus* than to *Manania* (Figs. 3–5). Both *Depastromorpha* and *Manania* possess the claustrum (Figs. 9 and 11), a structure also present in *Depastrum* and *Halimocyathus* in the family Haliclystidae (Table 8) (Clark, 1863; Carlgren, 1935; Kramp, 1961), suggesting that this structure may have been lost in the lineage leading to *Haliclystus* (Figs. 7 and 11; ACCTRAN optimization). In general, *Depastrum*, *Depastromorpha*, *Manania*, and *Halimocyathus* have similar morphologies, in addition to the presence of claustrum. For example, *Depastrum*, *Depastromorpha*, and *Manania* (and probably *Halimocyathus*, see Clark, 1863) have an entire coronal muscle (Table 8; Carlgren, 1935; Kramp, 1961); *Manania*, *Depastromorpha*, and *Halimocyathus* have pad-like adhesive structures in the outermost secondary tentacles (Table 7; Clark, 1863; Carlgren, 1935; Kramp, 1961; Larson & Fautin, 1989; Zagal et al., 2011); and *Depastrum* and *Manania* have coronal muscle on the exumbrellar (external) side of the primary tentacles/anchors (Fig. 12), unlike all the other genera of stalked jellyfishes (although the condition in *Halimocyathus* is uncertain) (Carlgren, 1935).

**Figure 13:**
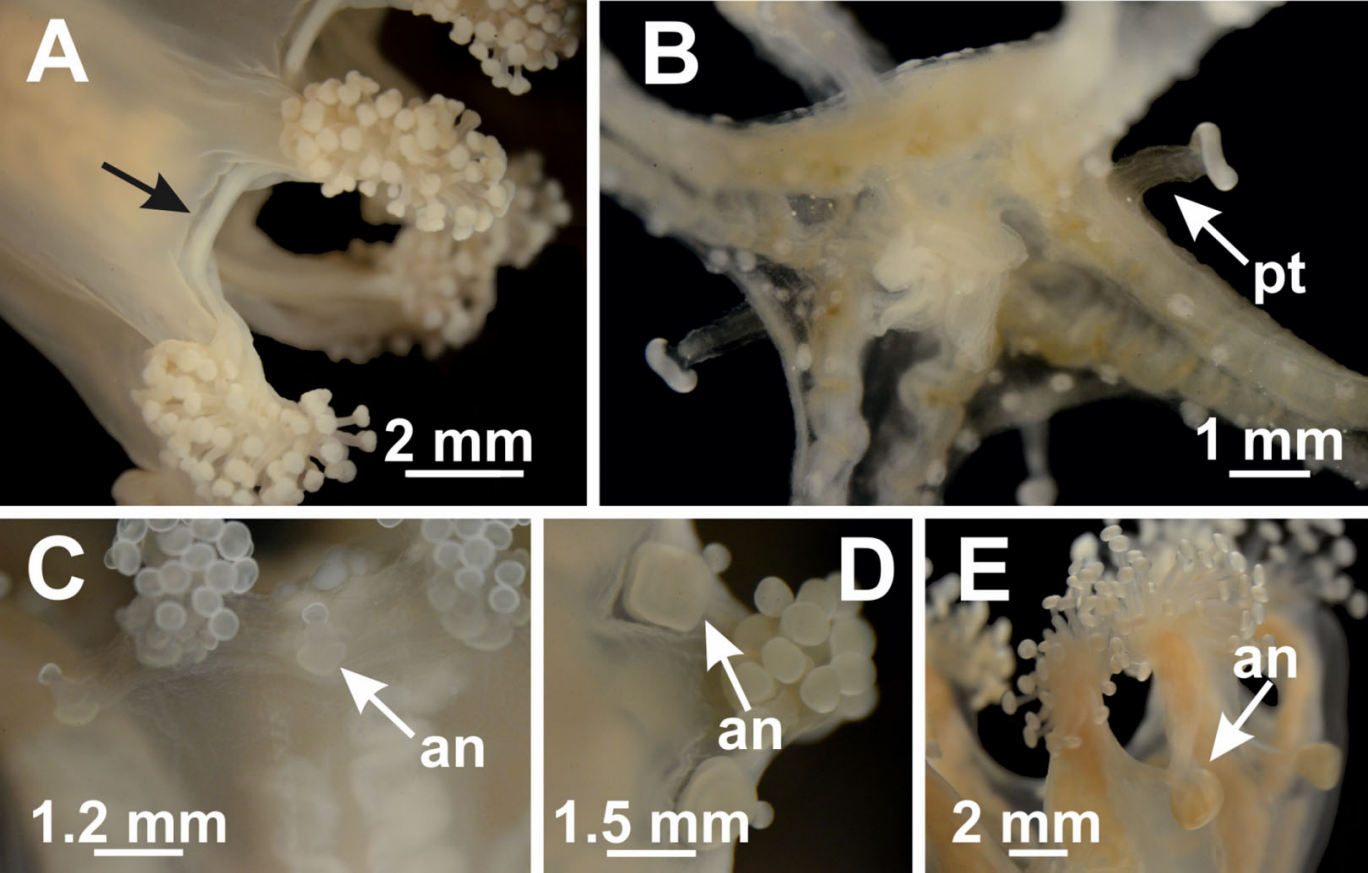
Primary tentacles and anchors. *Craterolophus convolvulus*: (A) absence of primary tentacles and anchors (indicated by black arrow) between arms; *Calvadosia cruciformis*: (B) presence of primary tentacles (pt); *Manania uchidai*: (C) anchors (an) with a knobbed remnant of primary tentacles; *Depastromorpha africana*: (D) anchors (an) with a knobbed remnant of primary tentacles; *Haliclystus tenuis*: (E) anchors (an). Photo credit: Lucília Miranda.

Most species of the family Haliclystidae have primary tentacles that metamorphose into anchors (Figs. 11 and 13). In the genera *Manania* and *Depastromorpha*, there is a knobbed remnant of each primary tentacle, with a glandular pad-like adhesive structure at the base (Figs. 13C and 13D) (Carlgren, 1935; Larson & Fautin, 1989; Zagal et al., 2011). However, the anchors in *Manania* are small and sometimes referred to as primary tentacles (Naumov, 1961; Larson & Fautin, 1989). In *Halimocyathus*, the anchors were described as “pistilliform,” “very small,” with “uniform thickness from the knob to the base” (Clark, 1863: 536, 538), but broader than the secondary tentacles (Mayer, 1910), so they are probably similar to the anchors in *Manania*, but possibly even more diminutive. In *Haliclystus*, the transformation of the primary tentacles into anchors is more obvious (Fig. 13E) (Clark, 1863; Hirano, 1986; Miranda, Morandini & Marques, 2009), although a knobbed remnant of the primary tentacles can be observed in some species (Clark, 1878; Miranda, Morandini & Marques, 2009; Kahn et al., 2010). However, the genus *Depastrum* seems to be an exception, with unmetamorphosed perradial and interradial primary tentacles (Clark, 1863; Mayer, 1910) (Table 8).

Based on morphological evidence, we include *Depastrum* Gosse, 1858 and *Haliclystus* Clark, 1863 in the same family (Figs. 7 and 8F; Table 7). However, there is a nomenclatural issue related to these genera. Haeckel (1879) proposed both the subfamilies Depastridae and Haliclystidae in the same book (Fig. 8B). Both names were used by Uchida (1929), but Carlgren (1935), Kramp (1961) and Daly et al. (2007) used only Depastrinae/Depastridae, and replaced Haliclystidae by Lucernariidae (Fig. 8). Consequently, the prevailing name would be Depastridae. However, there are two caveats: (1) *Depastrum cyathiforme*, the single species of the genus (Table 7), is not sampled in this study and consequently its position in the phylogeny (i.e., its relationship with other genera) is more tentative (Fig. 7; based only on morphological similarities); and (2) the last report of *D. cyathiforme* in the literature was about 40 years ago (den Hartog, 1976). Therefore, we believe it is better for nomenclatural stability to use the name Haliclystidae over Depastridae, and as first revisers refer to the International Code on Zoological Nomenclature (ICZN), article 24.2.2.

#### Family Kyopodiidae Larson, 1988

*Type genus*: *Kyopoda* Larson, 1988

The Kyopodiidae is a monospecific family proposed by Larson (1988) as part of Eleutherocarpida. *Kyopoda lamberti* Larson, 1988 has an unusual morphology: its calyx is reduced and the gonads and gastric cavity reside at the base of the peduncle (Larson, 1988).

There was no specimen available of *K. lamberti* to be included in our phylogenetic analyses. In addition, its particular morphology hampers attempts to identify a relationship with other genera of Staurozoa, which makes future study focusing on the homologies of *K. lamberti* with other Staurozoa especially interesting. Therefore, we presently retain the monogeneric family Kyopodiidae and assign it to the suborder Myostaurida (Figs. 7 and 8F; Table 7) because *K. lamberti* has interradial longitudinal muscles associated with the infundibula (Larson, 1988).

#### Family Lipkeidae Vogt, 1886

*Type genus*: *Lipkea* Vogt, 1886

The monogeneric family Lipkeidae was proposed by Vogt (1886) and presently encompasses three species: *Lipkea ruspoliana* Vogt, 1886, *Lipkea sturdzii* (Antipa, 1893), and *Lipkea stephensoni* Carlgren, 1933 (cf. Carlgren, 1935; Kramp, 1961; Daly et al., 2007) (Table 7). *Lipkea* is closely related to *Lucernaria* (Figs. 3–5), but there are enough characters to easily distinguish these two genera (Table 8) and we retain *Lipkea* as the exclusive genus of Lipkeidae (Fig. 8F; Table 7).

#### Family Lucernariidae Johnston, 1847

*Type genus*: *Lucernaria* Müller, 1776

The family Lucernariidae was proposed by Johnston (1847), including only the genus *Lucernaria*. Whereas Clark (1863) used the name Lucernariae for all of Stauromedusae, Haeckel (1879) was actually the originator of the name Stauromedusae, in which he placed the family Lucernariidae, divided into two subfamilies: 1) Haliclystidae, including the genera *Haliclystus* and *Lucernaria*; and 2) Halicyathidae, including *Halicyathus* (=*Halimocyathus*) and *Craterolophus* (Fig. 8B). Carlgren (1935) proposed Lucernariinae as a subfamily of Clark’s (1863) family Eleutherocarpidae, including *Lucernaria*, *Haliclystus*, and *Stenoscyphus* (Fig. 8D), and a similar classification was used by Kramp (1961). Kikinger & Salvini-Plawen (1995), and then Daly et al. (2007), used Lucernariidae as a family of suborder Eleutherocarpina and suborder Eleutherocarpida, respectively, including the genera *Haliclystus*, *Stenoscyphus*, *Lucernaria*, and *Stylocoronella* (Fig. 8E). However, the topologies presented by Collins & Daly (2005) contradicted monophyly of this grouping (cf. Haeckel, 1879, i.e., when including at least *Lucernaria* and *Haliclystus*), a pattern corroborated in our results (Figs. 3–5). Accordingly, we propose that Lucernariidae be limited to the genera *Lucernaria* and *Stylocoronella* (Figs. 7 and 8F; Table 7). This hypothesis has to be tested further because *Stylocoronella* has not yet been available for inclusion in our molecular-based phylogenetic analysis (Fig. 7), but it is consistent with the morphological similarities of *Lucernaria* and *Stylocoronella* (Table 8). Kikinger & Salvini-Plawen (1995) superficially remarked that *Stylocoronella* spp. appear to be congeneric with *Lucernaria*, although they presented a fundamental difference concerning the fate of the primary tentacles. In *Lucernaria*, the primary tentacles reduce to absent through development (Berrill, 1962), whereas in *Stylocoronella* the primary tentacles are retained (Table 8) and become integrated among the adradial clusters of the secondary tentacles (Kikinger & Salvini-Plawen, 1995). However, this developmental difference cannot be distinguished in adults, making its application difficult. Additionally, the coronal muscle seems to be vestigial in *Stylocoronella* (Table 8) (Kikinger & Salvini-Plawen, 1995), but this information needs further observations.

### Character state evolution

Stalked jellyfishes have relatively few external characters useful for taxonomy (Hirano, 1997). Consequently, some internal features are also employed to differentiate these animals (Uchida, 1929; Ling, 1937; Ling, 1939; Miranda, Collins & Marques, 2013). However, most of these characters vary intraspecifically and ontogenetically and they have to be assessed and cautiously employed to differentiate species (Miranda, Morandini & Marques, 2009). We review the main characters used in the traditional taxonomy of Staurozoa (Table 8) and interpret their significance based on the new phylogenetic hypothesis for the class (Figs. 3–5 and 7; Table 7).

#### Claustrum

The claustrum (Fig. 9) is a membrane that divides the gastrovascular cavity (Clark, 1863; Gross, 1900) of some stauromedusae (Table 8) and represents an additional level of complexity of their gastrovascular system (Berrill, 1963; Collins & Daly, 2005). Stauromedusae with claustrum have eight gastric radial pockets in the calyx (Fig. 9; Gross, 1900; Berrill, 1963). The four external pockets, known as accessory radial pockets (or exogon pockets; Thiel, 1966), extend into the marginal tentacles and anchors, continuing into the peduncle as the gastric chambers (Berrill, 1963). The four internal pockets, known as principal radial pockets (or mesogon pockets; Thiel, 1966), are the true radial pockets of these stauromedusae because they contain the gonads, as do the four gastric radial pockets of species without claustrum (Clark, 1863; Gross, 1900; Berrill, 1963). Clark (1863) proposed that the stalked jellyfishes should be divided into two main groups based on the presence and absence of the claustrum, respectively: Cleistocarpidae and Eleutherocarpidae (Fig. 8A). Since then, the claustrum has played an important role in the systematics of stauromedusae (Collins & Daly, 2005) and the main classifications have been based on this character, although with different levels of importance (Clark, 1863; Haeckel, 1879; Gross, 1900; Uchida, 1929; Carlgren, 1935; Gwilliam, 1956; Kramp, 1961; Uchida, 1973) (Fig. 8).

A preliminary phylogeny based on nuclear and mitochondrial molecular markers suggested that neither Cleistocarpida nor Eleutherocarpida are monophyletic and that the claustrum “is a more labile feature than suspected and that it may have been lost on more than one occasion,” and should not be used to diagnose subgroups within the class Staurozoa (Collins & Daly, 2005: 229). These conclusions are corroborated by our analysis (Figs. 3–5 and 11; Table 8). Most of the genera in the family Haliclystidae (suborder Myostaurida) have claustrum (*Depastromorpha*, *Depastrum*, *Halimocyathus*, and *Manania*), except the type genus *Haliclystus* (Fig. 8F; Tables 7 and 8). In addition, species of *Craterolophus*, family Craterolophidae (suborder Amyostaurida), also have claustrum (Tables 7 and 8), indicating a homoplastic character (Fig. 11).

Claustrum has also been described in the medusa stage of Cubozoa (Thiel, 1966). However, the internal organization of this structure is different between Staurozoa and Cubozoa (gonads associated with the exogon in Cubozoa; Thiel, 1966), and the existence of a typical staurozoan claustrum in Cubozoa is doubtful (Thiel, 1966). Therefore, if the claustrum in Staurozoa is not homologous to the structure in Cubozoa, claustrum appeared at least twice in the evolution of stalked jellyfishes, and it was lost in *Haliclystus* (Fig. 11, ACCTRAN). Alternatively, if considered a symplesiomorphy of Staurozoa (Collins & Daly, 2005), claustrum was lost in *Calvadosia*, *Haliclystus*, and in the clade *Lucernaria* + *Lipkea* (most parsimonious reconstruction).

#### Interradial longitudinal muscles in the peduncle

The stalked jellyfishes can have four interradial longitudinal muscle bundles, formed by epitheliomuscular cells, in the peduncle (Fig. 10A) (Miranda, Collins & Marques, 2013). These muscles have been generally used to distinguish genera and families of Stauromedusae (Table 8). Clark (1863), for example, distinguished the genus *Calvadosia* from *Lucernaria* based on the absence and presence of these muscles, respectively. Uchida (1929) separated stauromedusae without claustrum into three families, one of them (Kishinouyeidae) without muscles in the peduncle. At the same time, Uchida (1929) divided stauromedusae with claustrum into two subfamilies, Depastrinae with muscles in the peduncle, and Craterolophinae without these muscles (Fig. 8C).

Additionally, Uchida (1929) proposed using the shape of the muscle in the peduncle as seen in cross-section as a specific character of *Haliclystus stejnegeri* in relation to its congeners. Gwilliam (1956: 7) accepted the use of the muscular system to differentiate higher hierarchical levels (e.g., genera and families), but considered it virtually impossible to apply at the specific level due to considerable intraspecific variation, and because the shape depends on both the size (age) and degree of contraction of a given specimen.

Accordingly, the muscles in the peduncle have been treated inconsistently in classification schemes for Staurozoa. For instance, Uchida (1929) assigned *Kishinouyea* and *Sasakiella* to the family Kishinouyeidae, but incongruously assigned *Lucernariopsis* to the Haliclystidae, where it stands out by being the only other genus in the family without muscles in the peduncle (Fig. 8C). Finally, Uchida (1973) clearly considered the presence of claustrum as more important than the muscles in the peduncle in classification.

Our phylogenetic hypothesis reveals that Staurozoa can be divided into two main clades (Figs. 3–5): one only with species possessing the four interradial longitudinal muscles in the peduncle, and the other exclusively formed by species without interradial longitudinal muscles in the peduncle (Table 8). Accordingly, we propose two new suborders for class Staurozoa, order Stauromedusae based on the presence and absence of interradial longitudinal muscles in the peduncle, suborder Myostaurida and Amyostaurida, respectively (Figs. 7 and 8F; Table 7).

Collins et al. (2006) inferred that four interradial, intramesogleal longitudinal muscles associated with peristomial pits (infundibula) were symplesiomorphic in Staurozoa, and shared by the ancestral staurozoan with some (but not all) other medusozoans, a hypothesis we have used in our reconstruction (Fig. 11). Four intramesogleal muscles are characteristic of polyps of scyphozoans (Thiel, 1966; Marques & Collins, 2004; Collins & Daly, 2005). Cubopolyps also possess intramesogleal muscles, though the number is not fixed (Chapman, 1978; Marques & Collins, 2004). In hydropolyps, the musculature consists of a layer of longitudinal epidermal muscular fibers and circular gastrodermal fibers (Marques & Collins, 2004). According to this hypothesis, the longitudinal interradial muscles in the peduncle were lost in the clade Amyostaurida (Fig. 11). Additional clues to understand the likely evolutionary polarity of this character could come from detailed examination of its ontogenetic origins across Staurozoa. However, few stauropolyps have ever been studied (Wietrzykowski, 1912; Kikinger & Salvini-Plawen, 1995), and there is no information concerning the presence/absence of interradial longitudinal muscles in developing stauropolyps of Amyostaurida.

#### Chambers in the peduncle

**Figure 14:**
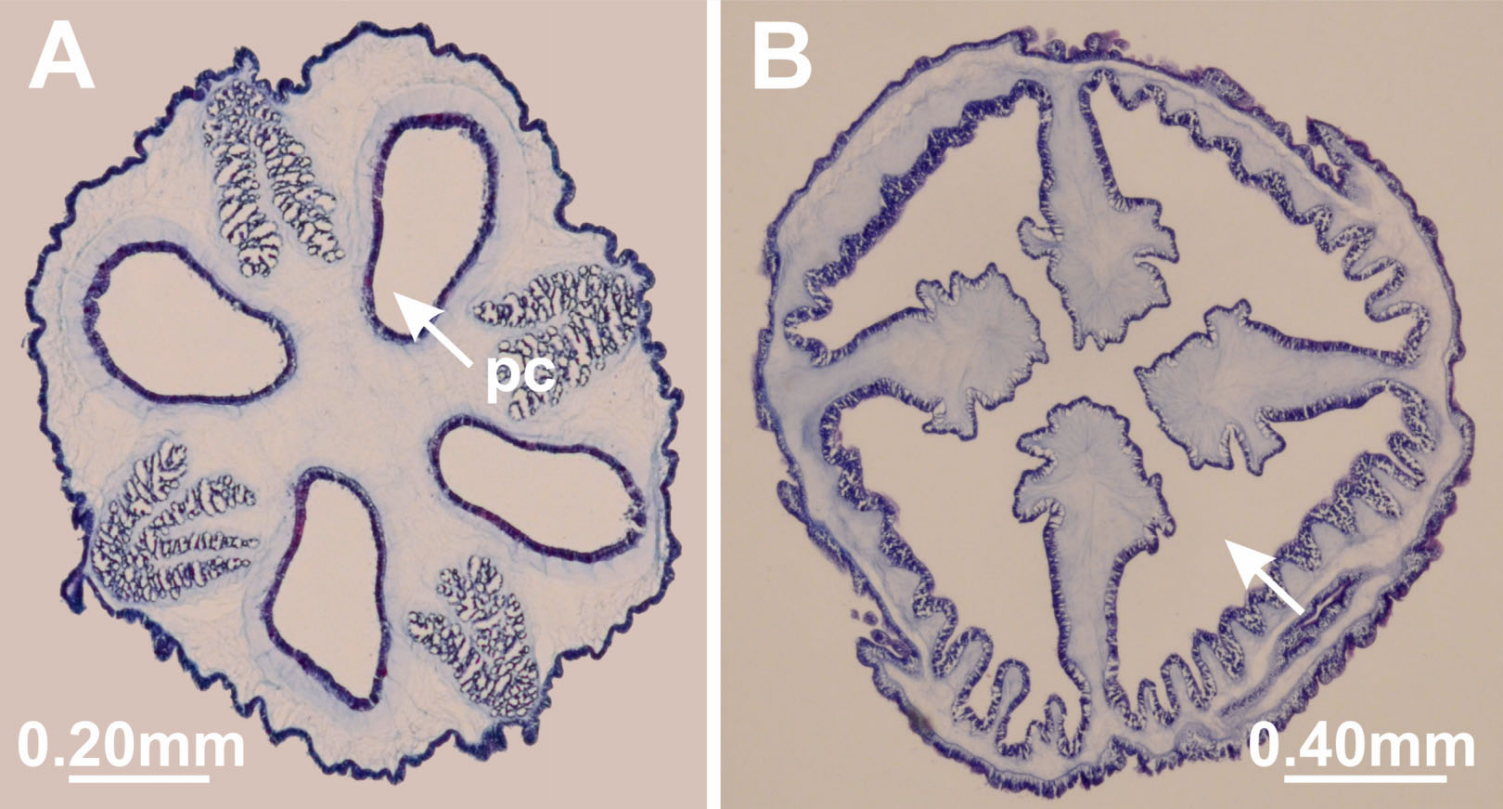
Chambers in the peduncle. *Haliclystus tenuis*: (A) four perradial chambers (pc) in peduncle; *Calvadosia corbini*: (B) one central gastric chamber (indicated by arrow) in the middle region of peduncle. Cross-sections. Photo credit: Lucília Miranda.

The peduncle of stauromedusae can have four perradial chambers delimited by gastrodermis (Fig. 14A) (Miranda, Collins & Marques, 2013), which are connected apically to the gastrovascular system of the calyx (Berrill, 1963). The number of chambers in the peduncle has been one of the characters most used in the literature to distinguish staurozoan genera (Clark, 1863; Mayer, 1910; Uchida, 1929; Kramp, 1961). The animals can either have one chamber in the peduncle (e.g., *Lucernaria*; Kramp, 1961); four chambers (e.g., *Haliclystus*; Kramp, 1961); four chambers in lower section of the peduncle, which fuse to form one chamber medially (e.g., *Kishinouyea*; Mayer, 1910); or one chamber in lower position with four chambers medially (e.g., some *Manania*, Larson & Fautin, 1989) (Table 8). When animals have four chambers in the medial position of the peduncle, these chambers fuse apically at the transition between peduncle and calyx (Uchida & Hanaoka, 1933; Miranda, Collins & Marques, 2013). Also, the number of chambers in the peduncle appears to vary during development of different species (Mayer, 1910; Uchida, 1929; Hirano, 1986), which makes its interpretation more complex. For instance, Wietrzykowski (1911) and Wietrzykowski (1912) observed *Haliclystus octoradiatus* with one chamber until the stage of 32 tentacles, when, progressively, four independent chambers are formed upward. This pattern was later observed in different species of *Haliclystus*, whose juveniles have a single-chambered peduncle, later divided into four chambers from the base to the top of the peduncle (Hirano, 1986).

*Manania* is probably the taxon with the widest variation concerning the number of peduncular chambers (Table 8): four chambers were reported throughout the peduncle in *Manania distincta*, *Manania gwilliami*, and *Manania handi* (Kishinouye, 1910; Larson & Fautin, 1989); four chambers medially and one chamber basally (the lower portion of the peduncle) in *Manania atlantica* and *Manania uchidai* (Naumov, 1961; Berrill, 1962); and one chamber throughout the peduncle in *Manania auricula* (Clark, 1863) and *Manania hexaradiata* (Broch, 1907; Kramp, 1961; Naumov, 1961). However, as the number of chambers in the peduncle in some *Manania* species is known to vary with ontogeny (Uchida, 1929; Hirano, 1986), the number of chambers is not a robust character to differentiate species and even staurozoan genera. For example, Clark (1863) considered *Halimocyathus* sufficiently different from *Manania*, both taxa described by him. One important difference in his descriptions is the four-chambered peduncle in the former, and single-chambered in the latter. However, different species of *Manania* were also later described with a four-chambered peduncle (Larson & Fautin, 1989). Therefore, as a general rule, even though the number of chambers in the peduncle seems to be an important character, it should be cautiously employed in the taxonomy of staurozoans (Uchida, 1929; Hirano, 1986).

There have also been some misinterpretations of the number of chambers in the peduncle, making it more difficult to employ this character in taxonomy. *Calvadosia nagatensis* (Mayer, 1910) and *Calvadosia hawaiiensis* (Edmondson, 1930) were reported with a four-chambered peduncle, but in fact they have one cruciform chamber throughout the peduncle and only at the level of the pedal disk can the four chambers be observed, sometimes separated by an axial canal (Uchida, 1929; Ling, 1939; Larson, 1980). In another example, *Haliclystus* was suggested to be closely related to *Lucernaria* because *Haliclystus antarcticus* and species of *Lucernaria* were reported to have a single chamber in the peduncle (Mayer, 1910: 536). In actuality, *H. antarcticus* has four chambers in the peduncle (Pfeffer, 1889; Carlgren, 1930; Miranda, Collins & Marques, 2013).

Ontogenetic data led Uchida (1929: 153) to hypothesize that “the single-chambered condition of the peduncle is more primitive than the four-chambered one.” However, there is a broad occurrence of four chambers in peduncles of Staurozoa, present at least in *Craterolophus*, *Depastromorpha*, *Depastrum*, *Haliclystus*, *Halimocyathus*, and some *Manania*, and this state would be a potential synapomorphy of Staurozoa (Fig. 11, ACCTRAN), as the four perradial chambers in the peduncle of stalked jellyfishes are not found in any other cnidarian life history stage (Collins & Daly, 2005).

#### Anchors (rhopalioids) and primary tentacles

During the early development of a stauropolyp, eight primary tentacles develop, four perradial and four interradial (Wietrzykowski, 1912; Hirano, 1986; Kikinger & Salvini-Plawen, 1995), which are probably homologous to the primary tentacles present in other medusozoans (Fig. 11; Uchida, 1929; Thiel, 1966). During the metamorphosis of a stauropolyp into an adult stauromedusa, these eight primary tentacles can have four different developmental fates: 1) they disappear by resorption (Berrill, 1963); 2) they metamorphose into adhesive structures called anchors (Hirano, 1986); 3) they remain as primary tentacles but with a modified shape (Ling, 1937); 4) they change their shape (filiform to capitate), migrate and cluster together with the secondary tentacles (Kikinger & Salvini-Plawen, 1995) (Fig. 13; Table 8).

In many species, primary tentacles are present in juvenile stauromedusae, but disappear during development (Uchida, 1929; Berrill, 1962; Larson, 1980). This suggested that an “erratic occurrence of these primary tentacles (…) indicates that they are negligible as diagnostic characters and of small significance” (Elmhirst, 1922: 221, also highlighted by Uchida, 1929: 150). There is fragmented information about this character, at least partly for a widespread lack of observation of young specimens of most species: Lamouroux (1815) reported that primary tentacles are sometimes observed in *C. campanulata*, probably in juveniles and in abnormal individuals; Uchida (1929), Ling (1939) and Larson (1980) reported the presence of rudiments of primary tentacles in very young specimens of *Calvadosia nagatensis* and *Calvadosia corbini*, as was also observed in *Craterolophus convolvulus* (Gross, 1900; Carlgren, 1935) and in species of *Lucernaria* (Berrill, 1963; Collins & Daly, 2005).

In some cases, the eight primary tentacles can also be retained throughout the life of the specimen (Fig. 13) and this condition was distinctive for the former genus *Sasakiella* (Ling, 1937), which comprised two species, presently *Calvadosia tsingtaoensis* and *Calvadosia cruciformis* (Table 7). These two species are differentiated by the number of primary tentacles retained, four in perradial positions in *C. tsingtaoensis*, and eight, in both the perradii and interradii, in *C. cruciformis* (Ling, 1937: 15). There may be, however, intraspecific variation for the character, probably related to development: in “a few extreme cases examined the four perradial primary tentacles [of *C. cruciformis*] are clearly seen but the four interradial ones are reduced to short rudiments. In young specimens all eight of them are well developed” (Ling, 1937: 19).

The development of *Stylocoronella riedli* and *Stylocoronella variabilis* shows that the primary filiform tentacles persist in these species, but are transformed into capitate tentacles and clustered together with the secondary tentacles at the tips of the adradial arms (Kikinger & Salvini-Plawen, 1995), a condition never reported in other genera of stalked jellyfishes (Table 8).

Particular marginal structures are also found in *Lipkea*, a genus morphologically quite distinct from all other stauromedusae (Uchida, 1929: 151) (Fig. 1N). Species of *Lipkea* have a variable number of lobes (or lappets) at the margin of the calyx (Pisani et al., 2007). *Lipkea ruspoliana* was described with perradial and interradial lobes, which were suggested to be homologous to the eight primary tentacles, not to the arms of other stauromedusae that are normally adradial (Uchida, 1929). According to this hypothesis, lobes would be highly metamorphosed primary tentacles (Uchida, 1929). However, *L. sturdzii* and *L. stephensoni* were described with adradial lobes (Antipa, 1893; Carlgren, 1933). The homology between lobes and primary tentacles was then questioned by Carlgren (1933), who referred to the lobes as modified arms, which was subsequently followed by the description of *Lipkea* with adradial marginal lobes and without perradial and interradial anchors (Kramp, 1961). Recently, the lobes of *L. ruspoliana* have been interpreted to be modified tentacles, with an adradial position (Pisani et al., 2007). We consider that the homology of these structures is still under debate, demanding further investigation, particularly of their development.

Primary tentacles can also metamorphose into anchors, adhesive structures that allow momentary adhesion to the substrate through their abundant glandular and supporting cells (Uchida, 1929; Hyman, 1940; Franc, 1994; Miranda, Collins & Marques, 2013). Species of Haliclystidae tend to have the primary tentacles metamorphosed totally or partially (i.e., with a knobbed remnant of the primary tentacles, Figs. 13C–13E) into anchors (Figs. 7 and 11; Tables 7 and 8).

The shape of anchors has frequently been used in the taxonomy of *Haliclystus* (Gwilliam, 1956; Miranda, Morandini & Marques, 2009; Kahn et al., 2010). However, their morphology has intraspecific and ontogenetic variation, and consequently it must be carefully assessed when employed to differentiate species of the genus (Miranda, Morandini & Marques, 2009; Kahn et al., 2010).

#### Pad-like adhesive structures

**Figure 15:**
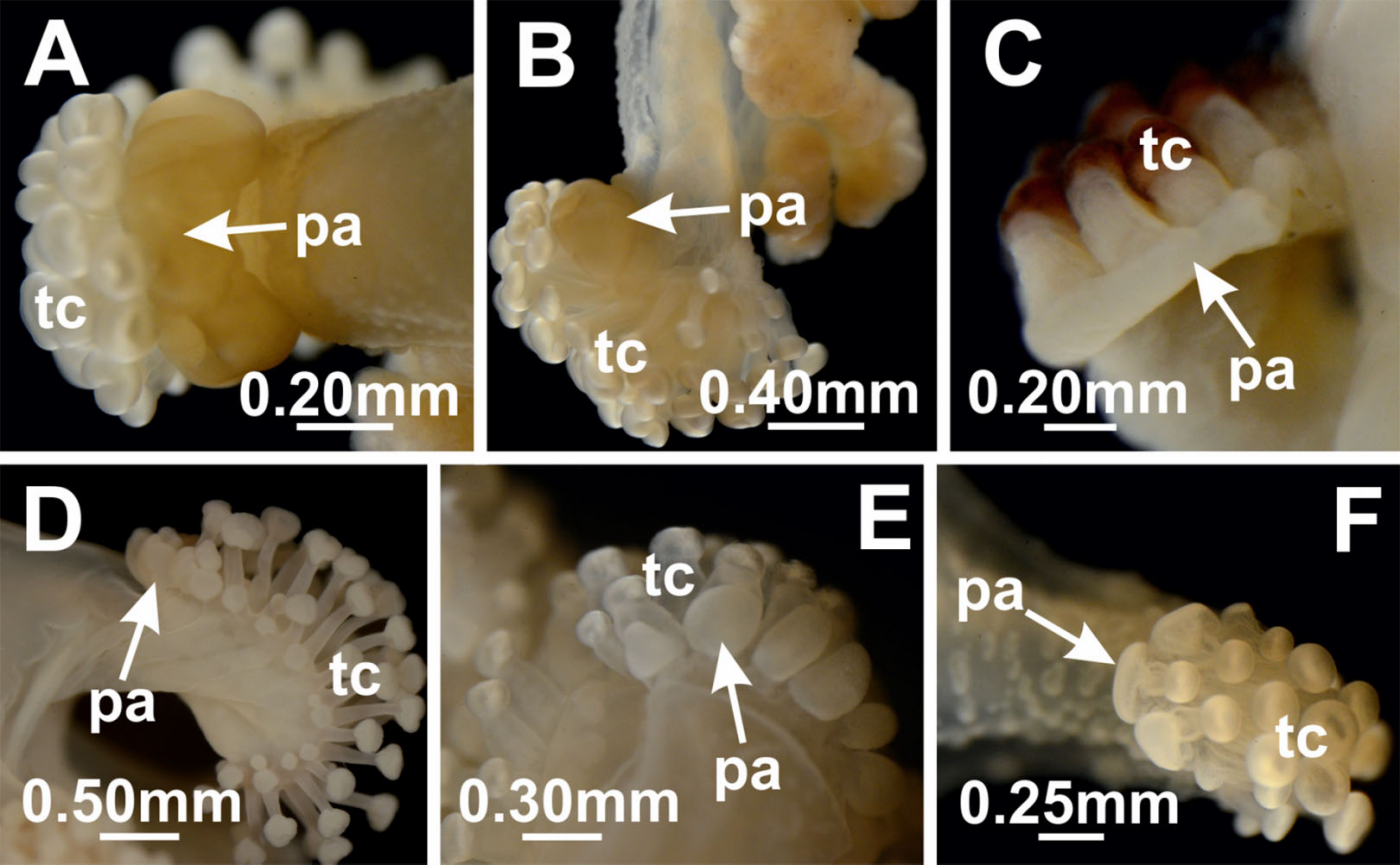
Pad-like adhesive structures. *Calvadosia tasmaniensis*: (A–B) pad (pa) on the tip of an arm separate from the secondary tentacles (tc); *Calvadosia cruxmelitensis*: (C) pad (pa) on the tip of an arm, with secondary tentacles (tc) arising directly from it; *Craterolophus convolvulus*: (D) pads (pa) in the outermost secondary tentacles (tc); *Calvadosia vanhoeffeni*: (E) pads (pa) in the outermost secondary tentacles (tc); *Calvadosia campanulata*: (F) pads (pa) in the outermost secondary tentacles (tc). Photo credit: Lucília Miranda.

Pad-like structures can be present individually in the outermost secondary tentacles of the tentacular cluster (Larson & Fautin, 1989), or as a broad structure on the tip of each arm (Larson, 1980; Miranda et al., 2012) (Fig. 15; Table 8). Apparently, the pads help the animal to adhere to its substrate. *Calvadosia corbini* was observed in situ attached to algae by the pedal disk or by the pad-like adhesive structures on the arms’ tips (Larson, 1980). In aquaria, *C. corbini* mainly use the pads to attach to the substratum, and the relatively large size of the pad compared to the pedal disk makes the importance of this structure for attachment clear (Larson, 1980). The glandular pads located on the anchor and on the abaxial tentacles of *Kyopoda lamberti* were hypothesized to temporarily serve to reattach the stauromedusae if it becomes detached (Larson, 1988).

There is only scattered information on the ontogeny of the pad-like adhesive structures. They apparently appear in the outermost tentacles late in development of *C. cruciformis* (Hirano, 1986: 197). Also, the broad adhesive pad-like structure on the tip of each arm hypothetically results from the fusion of several secondary outermost tentacles in *C. corbini* (Larson, 1980). Pad-like adhesive structures in the outermost tentacles and on the tips of the arms were considered to be homologous by Corbin (1978), but this requires more rigorous study.

This character has already been used to diagnose subfamilies (Carlgren, 1935). However, Carlgren (1935) overlooked the occurrence of pad-like adhesive structures in the outermost tentacles of some species of *Haliclystus*, which emphasizes the variation of this character within genera (Gwilliam, 1956). The pads in *Haliclystus* (especially in *Haliclystus californiensis*; Gwilliam, 1956; Kahn et al., 2010) are never as large as those found in *Manania* and *Calvadosia*, but their presence in *Haliclystus* should be taken into account in considering the relevance of this character for taxonomy.

The presence of these adhesive structures has been used in species descriptions. For instance, Larson (1980) included the pad-like adhesive structures on the tips of the arms as a distinguishing feature of *C. corbini*. However, he probably overlooked the presence of the structure in *C. hawaiiensis* because the character is neither well illustrated nor described in the original description by Edmondson (1930), but nevertheless present (Grohmann, Magalhães & Hirano, 1999).

**Figure 16:**
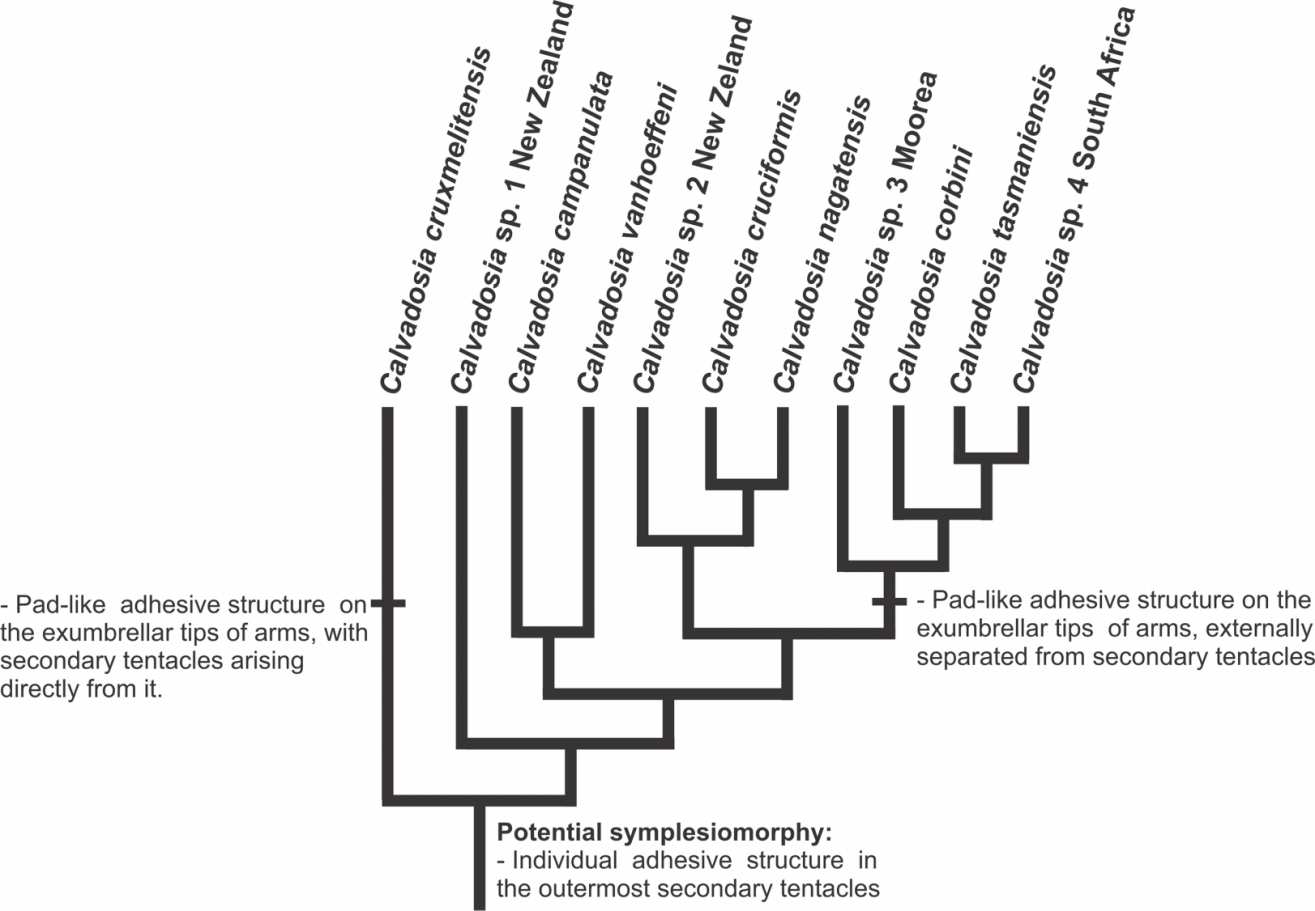
Evolution of pad-like adhesive structures in Kishinouyeidae. Most parsimonious reconstruction of pad-like adhesive structures in Kishinouyeidae according to our molecular phylogenetic hypothesis.

The presence of individual adhesive glandular pads in the outermost secondary tentacles is widespread in Staurozoa, occurring in *Craterolophus* (Carlgren, 1935), *Calvadosia* (Uchida, 1929; Carlgren, 1935), *Haliclystus* (Gwilliam, 1956; Kahn et al., 2010), *Depastromorpha* (Carlgren, 1935), *Halimocyathus* (Clark, 1863), *Manania* (Carlgren, 1935; Larson & Fautin, 1989), and *Kyopoda* (Larson, 1988). It is apparently absent in *Lucernaria* (Carlgren, 1935), *Stylocoronella* (Kikinger & Salvini-Plawen, 1995), and *Depastrum* (Clark, 1863; Carlgren, 1935), and perhaps not even applicable in *Lipkea* (Pisani et al., 2007), where they are not seen in any form. *Calvadosia* is the only genus including species with a broad pad-like adhesive structure on the tip of each arm. This structure is apparently a synapomorphy of the clade “(((*Calvadosia tasmaniensis*, *Calvadosia* sp. 4 South Africa), *Calvadosia corbini*), *Calvadosia* sp. 3 Moorea)” (Figs. 7 and 16). The feature is also present in *C. hawaiiensis* and *Calvadosia capensis*, suggesting that they too may belong to this clade. *Calvadosia cruxmelitensis* has a particular adhesive pad-like structure on the tip of each arm, in which the secondary tentacles arise directly from this structure, differing from other species with pad-like adhesive structures on the tips of the arms, in which the pad is externally separated from the stem of the secondary tentacles (Corbin, 1978) (Figs. 14A–14C and 16).

#### Coronal muscle

The coronal or marginal muscle is a band of epitheliomuscular cells at the calyx margin of stauromedusae (Gwilliam, 1956; Miranda, Collins & Marques, 2013). It is considered a synapomorphy of Medusozoa, probably lost in Hydrozoa (Collins et al., 2006), often associated with the swimming movement of jellyfishes (Arai, 1997). In the benthic medusae of Staurozoa, the contraction of the coronal musculature, along with contraction of the longitudinal muscles, considerably reduces the total volume of the animal, probably making its adherence to substrate more efficient in highly hydrodynamic habitats (Hyman, 1940; Miranda, Collins & Marques, 2013).

Coronal muscle can be either entire (undivided) or discontinuous (divided into perradial and interradial portions by the arms) (Figs. 11 and 12) (Clark, 1863; Carlgren, 1935; Gwilliam, 1956; Kramp, 1961). These two states have been used to differentiate genera hitherto (Table 8; Clark, 1863; Mayer, 1910; Uchida, 1929; Carlgren, 1935; Gwilliam, 1956; Kramp, 1961). In addition, the coronal muscle “appears to be vestigial or becomes ontogenetically depressed in *Stylocoronella*” (Kikinger & Salvini-Plawen, 1995: 908).

The position of coronal muscle in relation to the anchor/primary tentacles has also been used in the taxonomy of staurozoans (Carlgren, 1935; Gwilliam, 1956). In *Manania*, for example, the coronal muscle lies on the exumbrellar (external) side of the anchors (Gwilliam, 1956) (Fig. 12C), whereas in *Depastromorpha* the coronal muscle lies on the subumbrellar side (internal) of the anchors (Fig. 12D) (Carlgren, 1935). According to Carlgren (1935), only *Manania* and *Depastrum* have an external coronal muscle in relation to anchor/primary tentacles, but the phylogenetic signal of this character still has to be tested, specifically when specimens of *D. cyathiforme* become available for molecular study.

## Taxonomic synopsis of staurozoa

Class Staurozoa Marques & Collins, 2004.
Order Stauromedusae Haeckel, 1879.

### Suborder Amyostaurida nov.

*Diagnosis*: Stauromedusae without interradial longitudinal muscle in peduncle.

#### Family Craterolophidae Uchida, 1929

*Diagnosis*: No interradial longitudinal muscles in peduncle. Peduncle with four perradial chambers. Claustrum present. Without perradial and interradial anchors (rhopalioids) between arms. Individual pad-like adhesive structures can be present in outermost secondary tentacles. Coronal muscle divided.

##### Genus Craterolophus Clark, 1863

Type species: *Craterolophus convolvulus* (Johnston, 1835)

Diagnosis: Same as family.

Diversity: There are two valid species: *Craterolophus convolvulus* (Johnston, 1835) and *Craterolophus macrocystis* von Lendenfeld, 1884.

#### Family Kishinouyeidae Uchida, 1929

*Diagnosis*: No interradial longitudinal muscles in peduncle. Peduncle with one central gastric chamber and some species with four chambers at base of peduncle (pedal disk). Claustrum absent. No perradial and interradial anchors (rhopalioids) between arms (*C. cruciformis* with 4 interradial and 4 perradial primary tentacles, and *C. tsingtaoensis* with 4 perradial primary tentacles only). Species can have individual pad-like adhesive structures in outermost secondary tentacles or broad pads along tips of arms. Coronal muscle divided.

##### Genus Calvadosia Clark, 1863

Type species: *Calvadosia campanulata* (Lamouroux, 1815)

Diagnosis: Same as family.

Diversity: According to our phylogenetic and nomenclatural proposal (Figs. 3–5 and 7; Table 7), *Calvadosia* encompasses the species of the formerly-recognized genera *Kishinouyea*, *Sasakiella*, and *Lucernariopsis*. Therefore, *Calvadosia* has 10 species: *Calvadosia campanulata* (Lamouroux, 1815), *Calvadosia nagatensis* (Oka, 1897), *Calvadosia vanhoeffeni* (Browne, 1910), *Calvadosia cruciformis* (Okubo, 1917), *Calvadosia hawaiiensis* (Edmondson, 1930), *Calvadosia tsingtaoensis* (Ling, 1937), *Calvadosia capensis* (Carlgren, 1938), *Calvadosia cruxmelitensis* (Corbin, 1978), *Calvadosia corbini* (Larson, 1980), and *Calvadosia tasmaniensis* (Zagal et al., 2011).

Our molecular results suggest the probable existence of new species of the genus (Fig. 7; *Calvadosia* sp. 1 NZ, *Calvadosia* sp. 2 NZ, *Calvadosia* sp. 3 Moorea, *Calvadosia* sp. 4 SAF), which are being properly collected and/or morphologically analyzed in order to be tested and adequately described.

### Suborder Myostaurida nov.

*Diagnosis*: Stauromedusae with four interradial longitudinal muscular bands in peduncle.

#### Family Haliclystidae Haeckel, 1879

*Diagnosis*: Four interradial longitudinal muscles in peduncle. Perradial and interradial anchors/primary tentacles between arms. Gonads in calyx.

##### Genus Depastromorpha Carlgren, 1935

Type species: *Depastromorpha africana* Carlgren, 1935

Diagnosis: Four interradial longitudinal muscles in peduncle. Peduncle with four perradial chambers. Claustrum present. Perradial and interradial anchors (rhopalioids) between arms. Adhesive (glandular) cushions surrounding base of eight anchors, which have knobbed remnants of primary tentacles. Individual pad-like adhesive structures in outermost secondary tentacles. Rudimentary adradial arms. Entire coronal muscle internal to anchors.

Diversity: Monospecific, *Depastromorpha africana* Carlgren, 1935.

The species was recently recorded for Australia and New Zealand (Grohmann, Magalhães & Hirano, 1999; Cairns et al., 2009; Zagal et al., 2011); however, the molecular results show that the specimen from Australia, *Depastromorpha* sp. AUS (Fig. 7), could be a new species, but more detailed analysis is needed.

##### Genus Depastrum Gosse, 1858

Type species: *Depastrum cyathiforme* (Sars, 1846)

Diagnosis: Four interradial longitudinal muscles in peduncle. Peduncle with four perradial chambers. Claustrum present. No perradial and interradial anchors (rhopalioids) between arms, but one or more primary tentacles on perradius and interradius. No pad-like adhesive structures at secondary tentacles. No discernible arms, but eight (vestigial) sinuosities. Tentacles on each of the eight adradial groups arranged in one or several rows around calyx margin. Coronal muscle entire.

Diversity: Monospecific, *Depastrum cyathiforme* (Sars, 1846).

##### Genus Haliclystus Clark, 1863

Type species: *Haliclystus auricula* Clark, 1863

Diagnosis: Four interradial longitudinal muscles in peduncle. Peduncle with four perradial chambers. Claustrum absent. With perradial and interradial anchors (rhopalioids) between arms. Individual pad-like adhesive structures can be present in outermost secondary tentacles. Coronal muscle divided or entire.

Diversity: According to our phylogenetic hypothesis (Figs. 3–5 and 7), the genus *Stenoscyphus* should be synonymized to *Haliclystus. Haliclystus* is the most diverse genus of Staurozoa, represented by 13 species: *Haliclystus auricula* Clark, 1863; *Haliclystus octoradiatus* Clark, 1863; *Haliclystus salpinx* Clark, 1863; *Haliclystus inabai* (Kishinouye, 1893); *Haliclystus antarcticus* Pfeffer, 1889; *Haliclystus stejnegeri* Kishinouye, 1899; *Haliclystus kerguelensis* Vanhöffen, 1908; *Haliclystus tenuis* Kishinouye, 1910; *Haliclystus borealis* Uchida, 1933; *Haliclystus sinensis* Ling, 1937; *Haliclystus monstrosus* (Naumov, 1961); *Haliclystus californiensis* Kahn et al., 2010; and *Haliclystus “sanjuanensis” nomen nudum*.

The molecular results show a possible new species from Australia, *Haliclystus* sp. AUS (Fig. 7), previously identified as *Stenoscyphus inabai* (McInnes, 1989; Falconer, 2013), which is being collected and morphologically analyzed in order to be properly described.

##### Genus Halimocyathus Clark, 1863

Type species: *Halimocyathus platypus* Clark, 1863

Diagnosis: Four interradial longitudinal muscles in peduncle. Peduncle with four perradial chambers. Claustrum present. Small perradial and interradial anchors between arms. Eight distinct arms, twice as long as broad. Individual pad-like adhesive structures in outermost secondary tentacles. Coronal muscle entire (?).

Diversity: *Halimocyathus platypus* Clark, 1863 is the only species currently valid for the genus. A second species, *Halimocyathus lagena* (cf. Mayer, 1910; Kramp, 1914; Kramp, 1943; Kramp, 1961), is a synonym of *Manania auricula* (Clark, 1863; Larson & Fautin, 1989).

*Halimocyathus platypus* was described based on only one specimen (Clark, 1863), and its validity and relationship with *Manania* spp. still has to be tested in light of molecular and morphological data whenever new material becomes available.

##### Genus Manania Clark, 1863

Type species: *Manania auricula* (Fabricius, 1780)

Diagnosis: Four interradial longitudinal muscles in peduncle. Peduncle with four perradial chambers, or one central gastric chamber, or one chamber in lower position with four chambers medially. Claustrum present. Perradial and interradial anchors between arms. Adhesive (glandular) cushions surrounding bases of eight anchors, which have knobbed remnants of primary tentacles. Eight short arms. Individual pad-like adhesive structures in outermost secondary tentacles. Entire coronal muscle, external to anchors.

Diversity: The genus *Manania* comprises seven valid species: *Manania auricula* (Fabricius, 1780); *Manania hexaradiata* (Broch, 1907); *Manania distincta* (Kishinouye, 1910); *Manania atlantica* (Berrill, 1962); *Manania uchidai* (Naumov, 1961); *Manania gwilliami* Larson & Fautin, 1989; and *Manania handi* Larson & Fautin, 1989.

#### Family Kyopodiidae Larson, 1988

*Diagnosis*: Four interradial longitudinal muscles in peduncle. Peduncle with four chambers. Claustrum absent. Body vermiform. Basal portion of peduncle enlarged, with stomach and gonads, which are absent from calyx. No evident arms. Eight adradial groups of secondary tentacles in several ranks just proximal to calyx margin. Eight primary tentacles (also called anchors, four perradial and four interradial) between groups of secondary tentacles. Individual pad-like adhesive structures present in outermost secondary tentacles. Coronal muscle entire.

##### Genus Kyopoda Larson, 1988

Type species: *Kyopoda lamberti* Larson, 1988

Diagnosis: Same as family.

Diversity: *Kyopoda lamberti* Larson, 1988 is the single species described for the genus.

#### Family Lipkeidae Vogt, 1886

*Diagnosis*: Four interradial longitudinal muscles in peduncle. Peduncle with one central gastric chamber. Claustrum absent. Perradial and interradial anchors absent. Eight (or more) marginal lobes (lappets). Pad-like adhesive structures absent. Coronal muscle entire.

##### Genus Lipkea Vogt, 1886

Type species: *Lipkea ruspoliana* Vogt, 1886

Diagnosis: Same as family.

Diversity: Three valid species: *Lipkea ruspoliana* Vogt, 1886; *Lipkea sturdzii* (Antipa, 1893), and *Lipkea stephensoni* Carlgren, 1933.

The molecular results suggest a possible new species from Japan, *Lipkea* sp. Japan (Fig. 7), which is being morphologically analyzed in order to be properly described. Unidentified specimens of *Lipkea* have also been observed in Australia and in New Zealand (Zagal et al., 2011) and the species affinities of these stauromedusae requires further studies.

#### Family Lucernariidae Johnston, 1847

*Diagnosis*: Four interradial longitudinal muscles in peduncle. Peduncle with one central gastric chamber. Claustrum absent. Perradial and interradial anchors/primary tentacles absent between arms. Pad-like adhesive structures absent.

##### Genus Lucernaria Müller, 1776

Type species: *Lucernaria quadricornis* Müller, 1776

Diagnosis: Four interradial longitudinal muscles in peduncle. Peduncle with one central gastric chamber. Claustrum absent. Perradial and interradial anchors absent between arms. Pad-like adhesive structures absent. Coronal muscle divided.

Diversity: Eight valid species: *Lucernaria quadricornis* Müller, 1776; *Lucernaria bathyphila* Haeckel, 1879; *Lucernaria infundibulum* Haeckel, 1879; *Lucernaria haeckeli* (Antipa, 1892); *Lucernaria walteri* (Antipa, 1892); *Lucernaria australis* Vanhöffen, 1908; *Lucernaria sainthilairei* (Redikorzev, 1925); and *Lucernaria janetae* Collins & Daly, 2005.

The molecular results raised possible taxonomic issues because *L. bathyphila* is not monophyletic (Figs. 3–5), sharing a close relationship with other deep-sea *Lucernaria*, *L. janetae*. Therefore, a detailed study of evolutionary relationships among species of *Lucernaria* is needed.

##### Genus Stylocoronella Salvini-Plawen, 1966

Type species: *Stylocoronella riedli* Salvini-Plawen, 1966

Diagnosis: Four interradial longitudinal muscles in peduncle. Peduncle with one central gastric chamber. Claustrum absent. Perradial and interradial anchors absent between arms. Pad-like adhesive structures absent. Coronal muscle vestigial.

Diversity: Two valid species: *Stylocoronella riedli* Salvini-Plawen, 1966 and *Stylocoronella variabilis* Salvini-Plawen, 1987.

## Identification Key for the Genera of Staurozoa

1. Interradial longitudinal muscles in peduncle presentMyostaurida (2)
  Interradial longitudinal muscles in peduncle absentAmyostaurida (10)
2. Body vermiform, with sac-like swelling at base of peduncle, containing stomach and gonads, which are absent from calyxKyopoda
  Body not vermiform, without sac-like swelling at base of peduncle, gonads on calyx(3)
3. Primary tentacles typically absent/reduced/adradial in stauromedusa, consequently never metamorphosed into anchors(4)
  Primary tentacles present (perradial and interradial) in stauromedusa, which can be metamorphosed into anchors (with or without a knobbed remnant of primary tentacle)(6)
4. Coronal muscle vestigial or absent (poorly developed)Stylocoronella
  Coronal muscle present, either entire or divided(5)
5. Entire coronal muscleLipkea
  Divided coronal muscleLucernaria
6. Claustrum absentHaliclystus
  Claustrum present(7)
7. Long arms clearly recognizable (twice as long as broad)Halimocyathus
  Short or rudimentary arms(8)
8. Pad-like adhesive structure in secondary and primary tentacles absentDepastrum
  Pad-like adhesive structure at the base of anchors (modified primary tentacles) and in outermost secondary tentacles(9)
9. Coronal muscle on exumbrellar (external) side of anchorsManania
  Coronal muscle on subumbrellar (internal) side of anchorsDepastromorpha
10. Claustrum absentCalvadosia
  Claustrum present*Craterolophus*.

## Concluding Remarks

The traditional classification of Staurozoa was previously established based on subjective interpretations of anatomical similarities (Clark, 1863; Haeckel, 1879; Uchida, 1929; Uchida, 1973; Carlgren, 1935). We provide here the first classification based on a robust and comparatively complete phylogenetic analysis, including about half of the known species of Staurozoa (Figs. 3–5). Therefore, we propose a major taxonomic revision (Fig. 7; Table 7) at the suborder, family, and genus levels, in order to preserve the monophyly of taxa. Our phylogenetic analysis has also allowed for a reassessment of the evolution of the main characters used in traditional staurozoan classification. We were not able to present new data for the genera *Kyopoda*, *Stylocoronella*, *Depastrum*, and *Halimocyathus*, but provide hypotheses for their phylogenetic placements based on reported morphology (Fig. 7; Table 7). These hypotheses require new collection and detailed analysis of morphology and genetics in order to assess their validity.

Stalked jellyfishes are fascinating animals, with a peculiar anatomy related to their life cycle. Further evolutionary studies of their representatives are especially needed to gain a more complete understanding of potential homologies shared by this group and other cnidarians. In addition, such studies would support a broad spectrum of research endeavors not yet addressed for Staurozoa, such as conservation, macroecology, and biogeography.

## Supplemental Information

10.7717/peerj.1951/supp-1 Supplemental Information 1 Parsimony phylogenetic hypothesis based on the mitochondrial marker COI. Single most parsimonious tree, length: 163.59 steps. Bootstrap indices under parsimony at each node. ANT, Antarctica; AUS, Australia; EPR, East Pacific Rise; GER, Germany; JAP, Japan; NZ, New Zealand; SAF, South Africa; UK, the United Kingdom.

10.7717/peerj.1951/supp-2 Supplemental Information 2 Parsimony phylogenetic hypothesis based on the mitochondrial marker 16S. Single most parsimonious tree, length: 322.30 steps. Bootstrap indices under parsimony at each node. ANT, Antarctica; AUS, Australia; EPR, East Pacific Rise; GER, Germany; JAP, Japan; NZ, New Zealand; SAF, South Africa; UK, the United Kingdom; USA, the United States of America.

10.7717/peerj.1951/supp-3 Supplemental Information 3 Parsimony phylogenetic hypothesis based on the nuclear marker ITS. Consensus (50% majority-rule) of two equally most parsimonious trees, length: 122.18 steps. Bootstrap indices under parsimony at each node. ANT, Antarctica; AUS, Australia; EPR, East Pacific Rise; GER, Germany; JAP, Japan; NZ, New Zealand; SAF, South Africa; UK, the United Kingdom; USA, the United States of America.

10.7717/peerj.1951/supp-4 Supplemental Information 4 Parsimony phylogenetic hypothesis based on the nuclear marker 18S. Consensus (50% majority-rule) of five equally most parsimonious trees, length: 312.48 steps. Bootstrap indices under parsimony at each node. ANT, Antarctica; AUS, Australia; EPR, East Pacific Rise; GER, Germany; JAP, Japan; NZ, New Zealand; SAF, South Africa; USA, the United States of America.

10.7717/peerj.1951/supp-5 Supplemental Information 5 Parsimony phylogenetic hypothesis based on the nuclear marker 28S. Single most parsimonious tree, length: 783.04 steps. Bootstrap indices under parsimony at each node. ANT, Antarctica; AUS, Australia; EPR, East Pacific Rise; GER, Germany; JAP, Japan; NZ, New Zealand; SAF, South Africa; USA, the United States of America.

10.7717/peerj.1951/supp-6 Supplemental Information 6 Maximum likelihood phylogenetic hypothesis based on the mitochondrial marker COI. Bootstrap indices under maximum likelihood at each node. ANT, Antarctica; AUS, Australia; EPR, East Pacific Rise; GER, Germany; JAP, Japan; NZ, New Zealand; SAF, South Africa; UK, the United Kingdom.

10.7717/peerj.1951/supp-7 Supplemental Information 7 Maximum likelihood phylogenetic hypothesis based on the mitochondrial marker 16S. Bootstrap indices under maximum likelihood at each node. ANT, Antarctica; AUS, Australia; EPR, East Pacific Rise; GER, Germany; JAP, Japan; NZ, New Zealand; SAF, South Africa; UK, the United Kingdom; USA, the United States of America.

10.7717/peerj.1951/supp-8 Supplemental Information 8 Maximum likelihood phylogenetic hypothesis based on the nucler marker ITS. Bootstrap indices under maximum likelihood at each node. ANT, Antarctica; AUS, Australia; EPR, East Pacific Rise; GER, Germany; JAP, Japan; NZ, New Zealand; SAF, South Africa; UK, the United Kingdom; USA, the United States of America.

10.7717/peerj.1951/supp-9 Supplemental Information 9 Maximum likelihood phylogenetic hypothesis based on the nucler marker 18S. Bootstrap indices under maximum likelihood at each node. ANT, Antarctica; AUS, Australia; EPR, East Pacific Rise; GER, Germany; JAP, Japan; NZ, New Zealand; SAF, South Africa; USA, the United States of America.

10.7717/peerj.1951/supp-10 Supplemental Information 10 Maximum likelihood phylogenetic hypothesis based on the nucler marker 28S. Bootstrap indices under maximum likelihood at each node. ANT, Antarctica; AUS, Australia; EPR, East Pacific Rise; GER, Germany; JAP, Japan; NZ, New Zealand; SAF, South Africa; USA, the United States of America.

10.7717/peerj.1951/supp-11 Supplemental Information 11 Bayesian phylogenetic hypothesis based on the mitochondrial marker COI. Posterior probability at each node. ANT, Antarctica; AUS, Australia; EPR, East Pacific Rise; GER, Germany; JAP, Japan; NZ, New Zealand; SAF, South Africa; UK, the United Kingdom.

10.7717/peerj.1951/supp-12 Supplemental Information 12 Bayesian phylogenetic hypothesis based on the mitochondrial marker 16S. Posterior probability at each node. ANT, Antarctica; AUS, Australia; EPR, East Pacific Rise; GER, Germany; JAP, Japan; NZ, New Zealand; SAF, South Africa; UK, the United Kingdom; USA, the United States of America.

10.7717/peerj.1951/supp-13 Supplemental Information 13 Bayesian phylogenetic hypothesis based on the nuclear marker ITS. Posterior probability at each node. ANT, Antarctica; AUS, Australia; EPR, East Pacific Rise; GER, Germany; JAP, Japan; NZ, New Zealand; SAF, South Africa; UK, the United Kingdom; USA, the United States of America.

10.7717/peerj.1951/supp-14 Supplemental Information 14 Bayesian phylogenetic hypothesis based on the nuclear marker 18S. Posterior probability at each node. ANT, Antarctica; AUS, Australia; EPR, East Pacific Rise; GER, Germany; JAP, Japan; NZ, New Zealand; SAF, South Africa; USA, the United States of America.

10.7717/peerj.1951/supp-15 Supplemental Information 15 Bayesian phylogenetic hypothesis based on the nuclear marker 28S. Posterior probability at each node. ANT, Antarctica; AUS, Australia; EPR, East Pacific Rise; GER, Germany; JAP, Japan; NZ, New Zealand; SAF, South Africa; USA, the United States of America.

## Additional Information and Declarations

### DNA Deposition

The following information was supplied regarding the deposition of DNA sequences: GenBank Accession numbers are provided in Table 1.

### Data Deposition

The following information was supplied regarding data availability: TreeBase (TB2:S18971) http://purl.org/phylo/treebase/phylows/study/TB2:S18971.
