# Supplementary figures and images for: Systematics of stalked jellyfishes (Cnidaria: Staurozoa)
Source: PeerJ. 2016 May 5;4:e1951. doi: 10.7717/peerj.1951 (PMC4860332; doi:10.7717/peerj.1951)

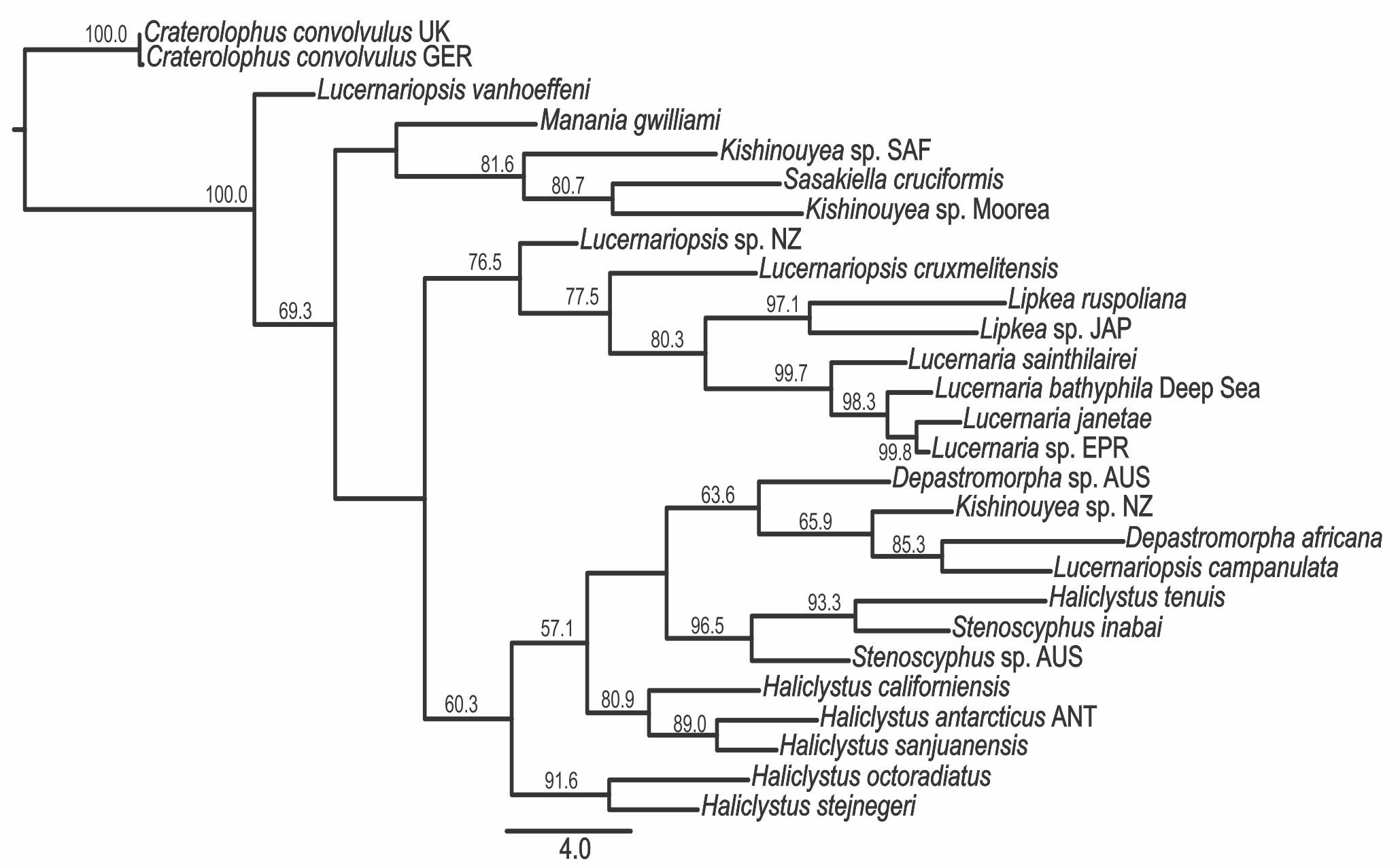

Supplement: Supplemental Information 1 — Single most parsimonious tree, length: 163.59 steps. Bootstrap indices under parsimony at each node. ANT, Antarctica; AUS, Australia; EPR, East Pacific Rise; GER, Germany; JAP, Japan; NZ, New Zealand; SAF, South Africa; UK, the United Kingdom. [file peerj-04-1951-s001.png]

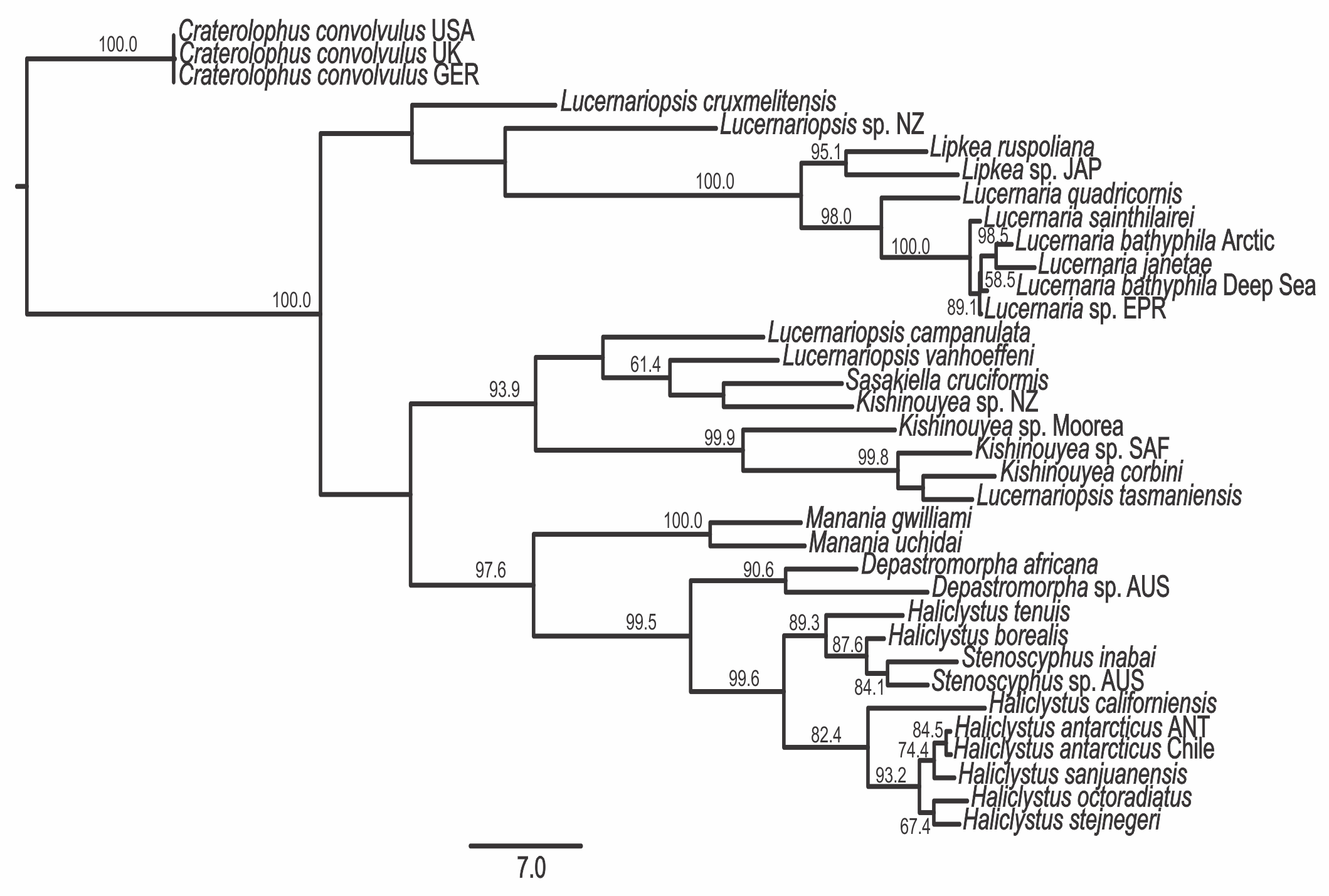

Supplement: Supplemental Information 2 — Single most parsimonious tree, length: 322.30 steps. Bootstrap indices under parsimony at each node. ANT, Antarctica; AUS, Australia; EPR, East Pacific Rise; GER, Germany; JAP, Japan; NZ, New Zealand; SAF, South Africa; UK, the United Kingdom; USA, the United States of America. [file peerj-04-1951-s002.png]

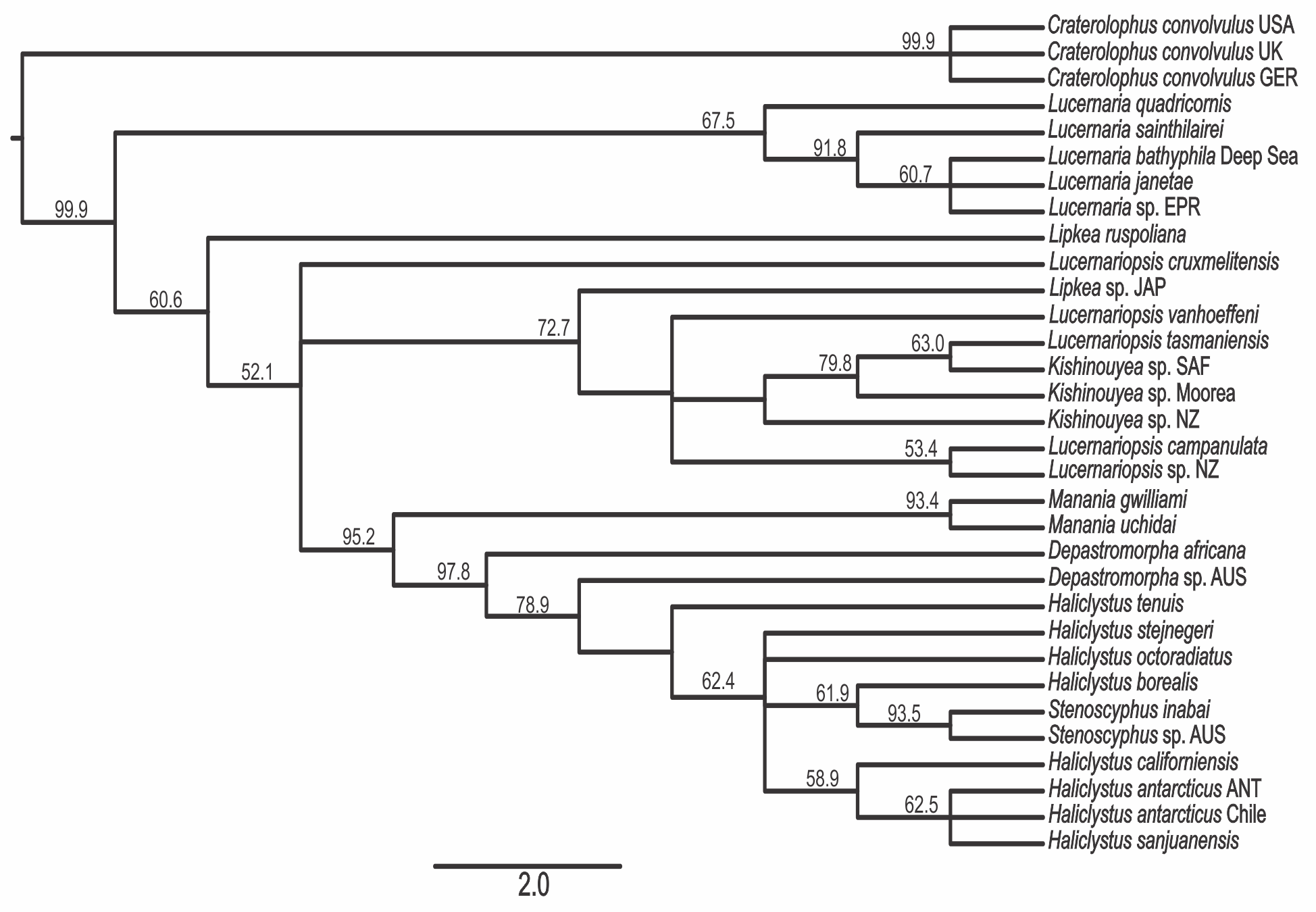

Supplement: Supplemental Information 3 — Consensus (50% majority-rule) of two equally most parsimonious trees, length: 122.18 steps. Bootstrap indices under parsimony at each node. ANT, Antarctica; AUS, Australia; EPR, East Pacific Rise; GER, Germany; JAP, Japan; NZ, New Zealand; SAF, South Africa; UK, the United Kingdom; USA, the United States of America. [file peerj-04-1951-s003.png]

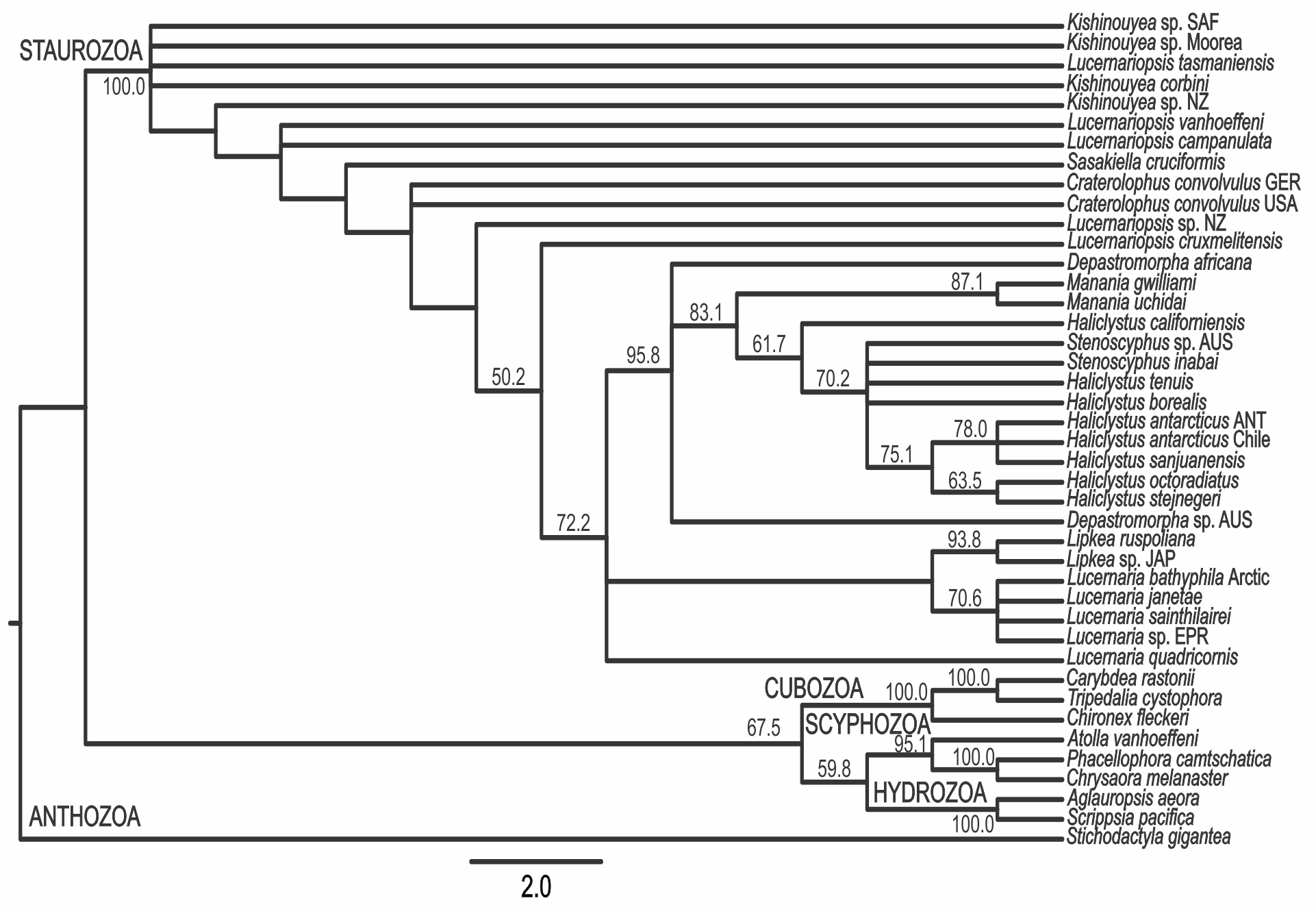

Supplement: Supplemental Information 4 — Consensus (50% majority-rule) of five equally most parsimonious trees, length: 312.48 steps. Bootstrap indices under parsimony at each node. ANT, Antarctica; AUS, Australia; EPR, East Pacific Rise; GER, Germany; JAP, Japan; NZ, New Zealand; SAF, South Africa; USA, the United States of America. [file peerj-04-1951-s004.png]

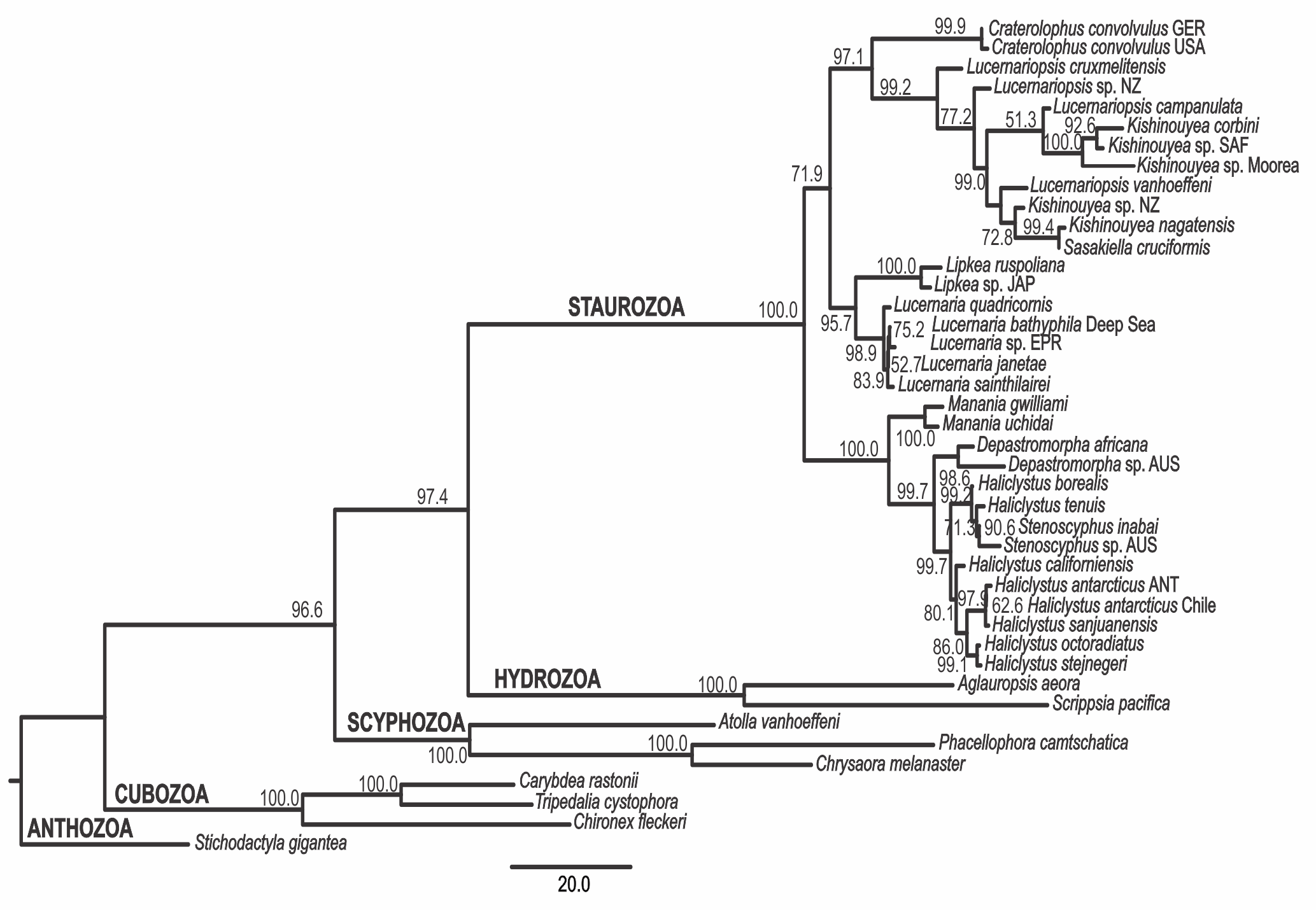

Supplement: Supplemental Information 5 — Single most parsimonious tree, length: 783.04 steps. Bootstrap indices under parsimony at each node. ANT, Antarctica; AUS, Australia; EPR, East Pacific Rise; GER, Germany; JAP, Japan; NZ, New Zealand; SAF, South Africa; USA, the United States of America. [file peerj-04-1951-s005.png]

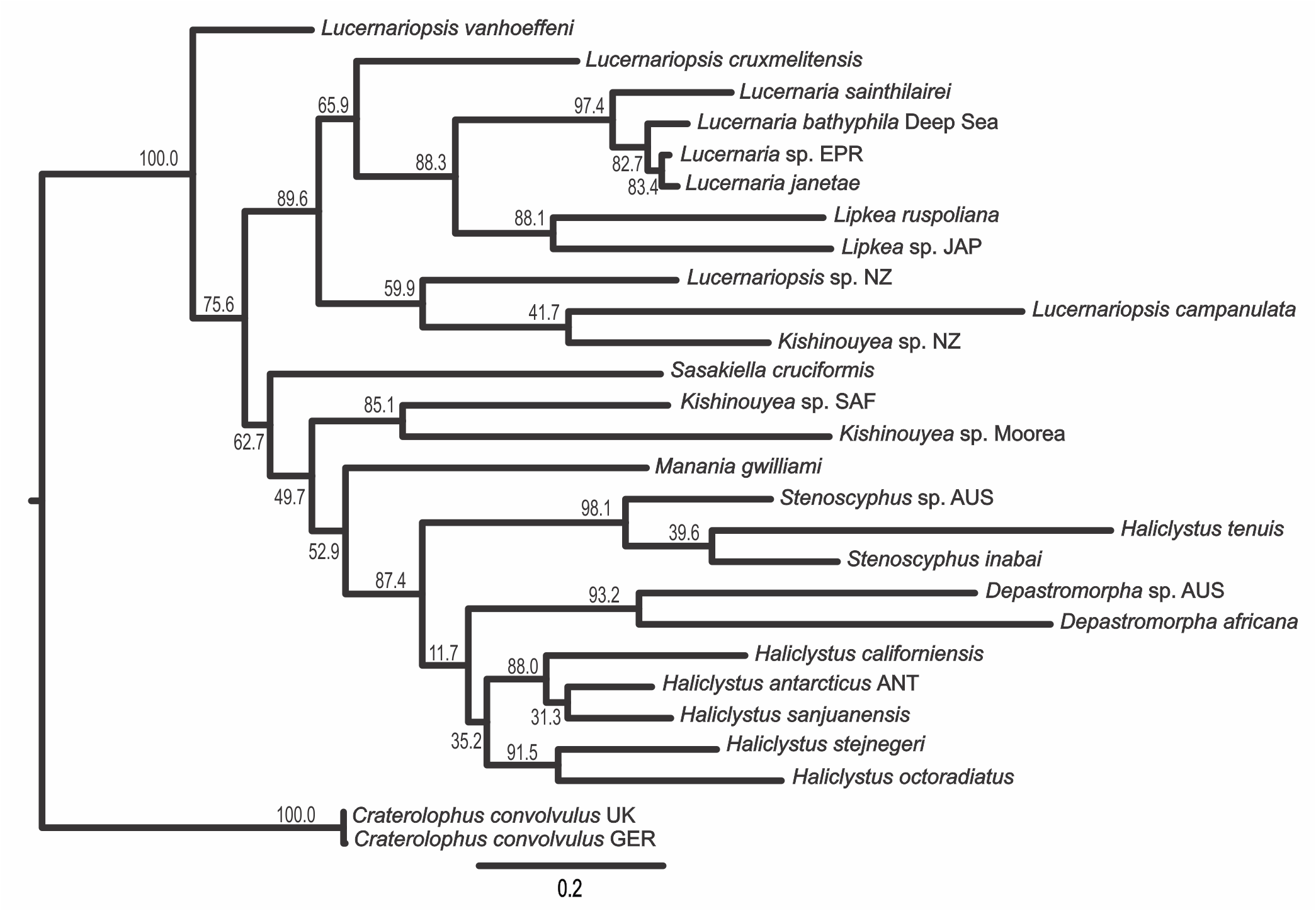

Supplement: Supplemental Information 6 — Bootstrap indices under maximum likelihood at each node. ANT, Antarctica; AUS, Australia; EPR, East Pacific Rise; GER, Germany; JAP, Japan; NZ, New Zealand; SAF, South Africa; UK, the United Kingdom. [file peerj-04-1951-s006.png]

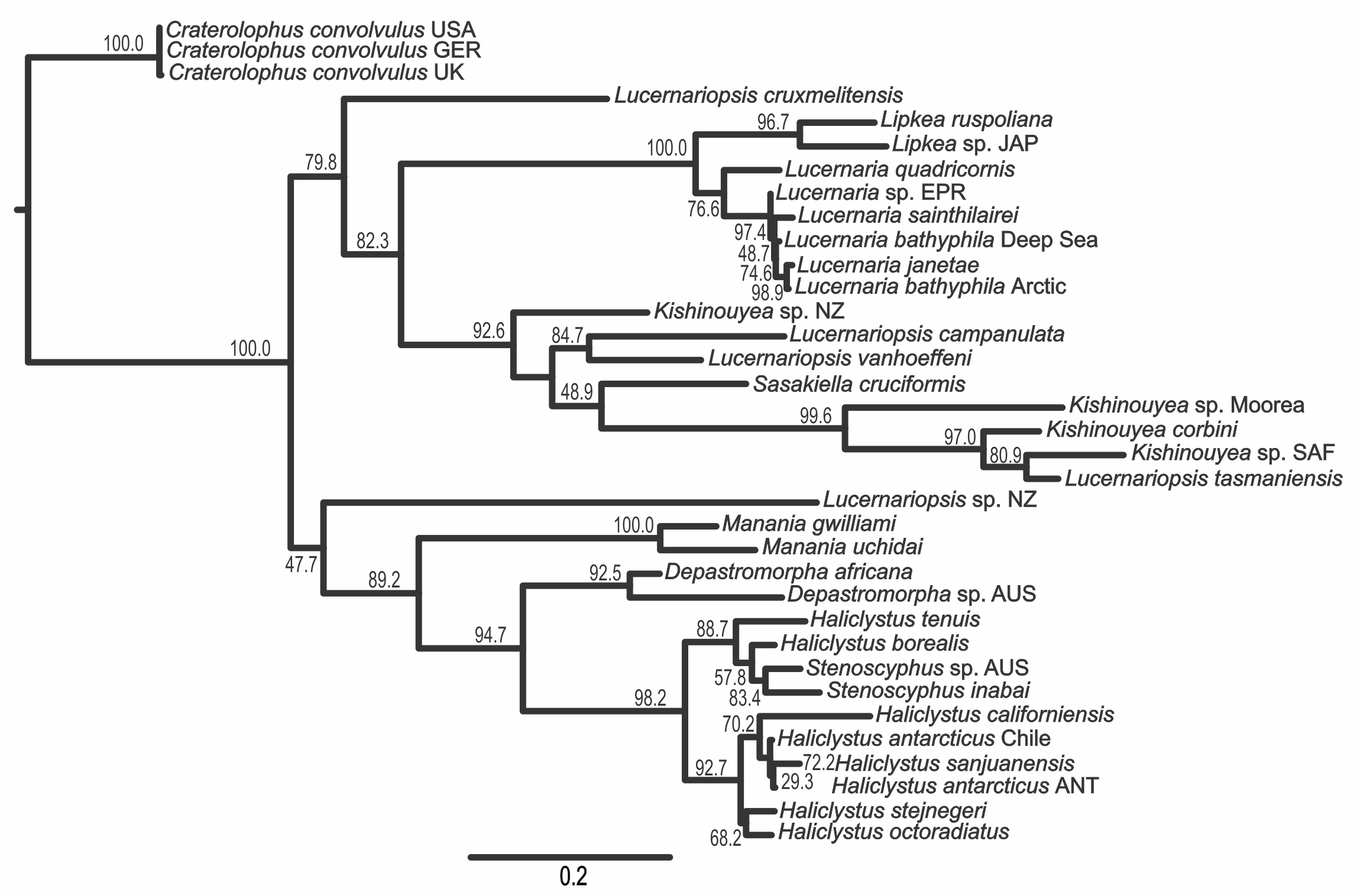

Supplement: Supplemental Information 7 — Bootstrap indices under maximum likelihood at each node. ANT, Antarctica; AUS, Australia; EPR, East Pacific Rise; GER, Germany; JAP, Japan; NZ, New Zealand; SAF, South Africa; UK, the United Kingdom; USA, the United States of America. [file peerj-04-1951-s007.png]

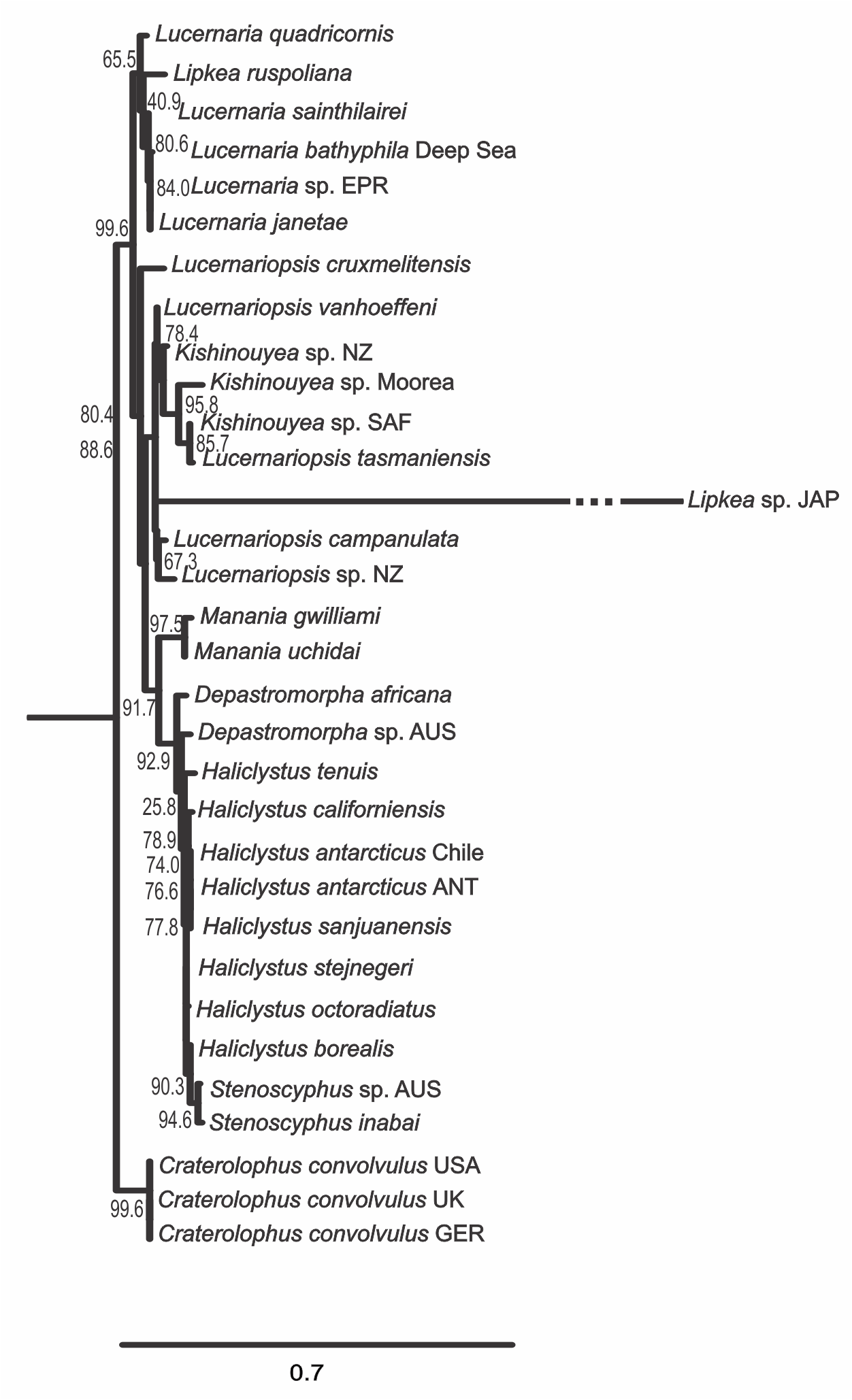

Supplement: Supplemental Information 8 — Bootstrap indices under maximum likelihood at each node. ANT, Antarctica; AUS, Australia; EPR, East Pacific Rise; GER, Germany; JAP, Japan; NZ, New Zealand; SAF, South Africa; UK, the United Kingdom; USA, the United States of America. [file peerj-04-1951-s008.png]

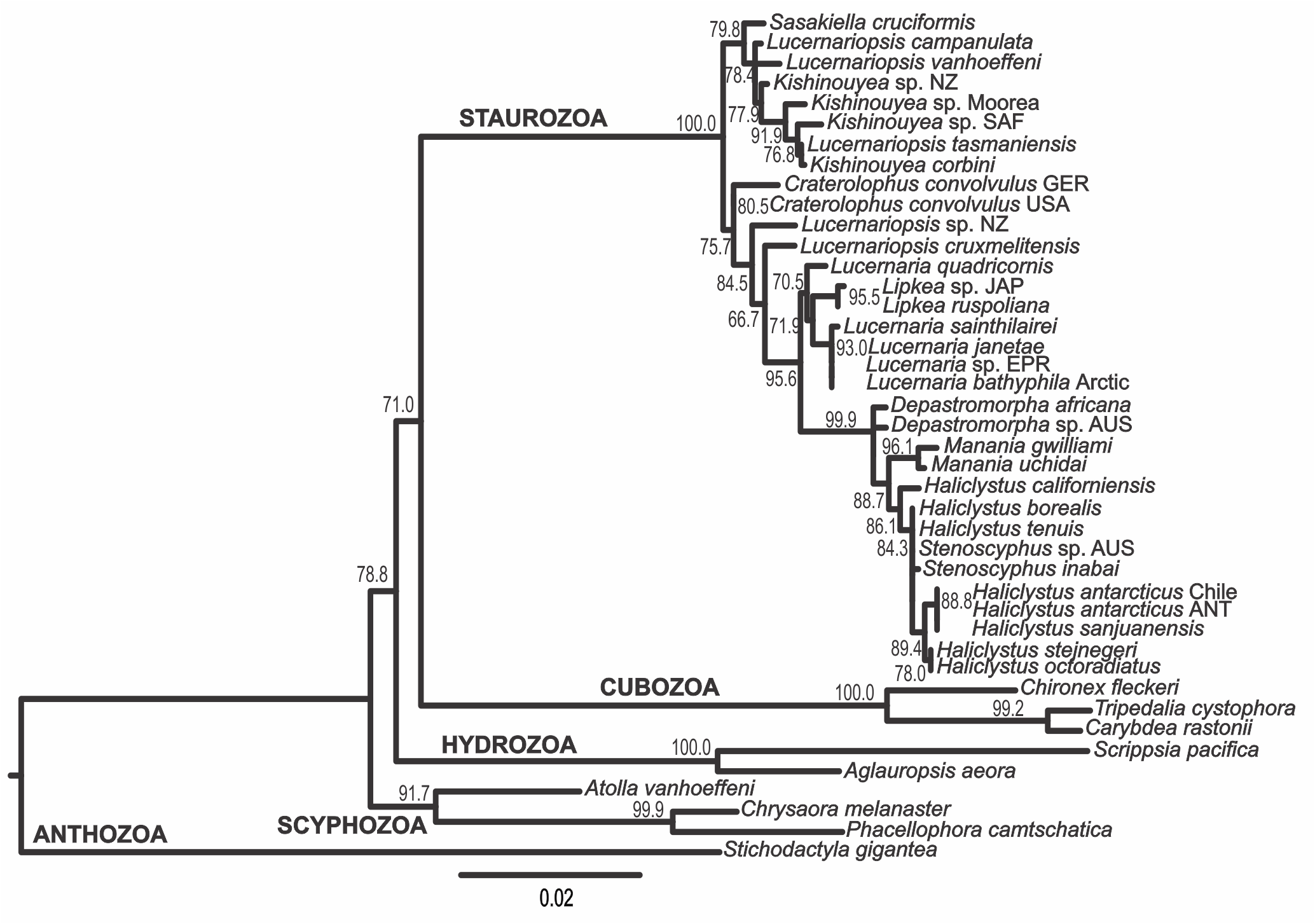

Supplement: Supplemental Information 9 — Bootstrap indices under maximum likelihood at each node. ANT, Antarctica; AUS, Australia; EPR, East Pacific Rise; GER, Germany; JAP, Japan; NZ, New Zealand; SAF, South Africa; USA, the United States of America. [file peerj-04-1951-s009.png]

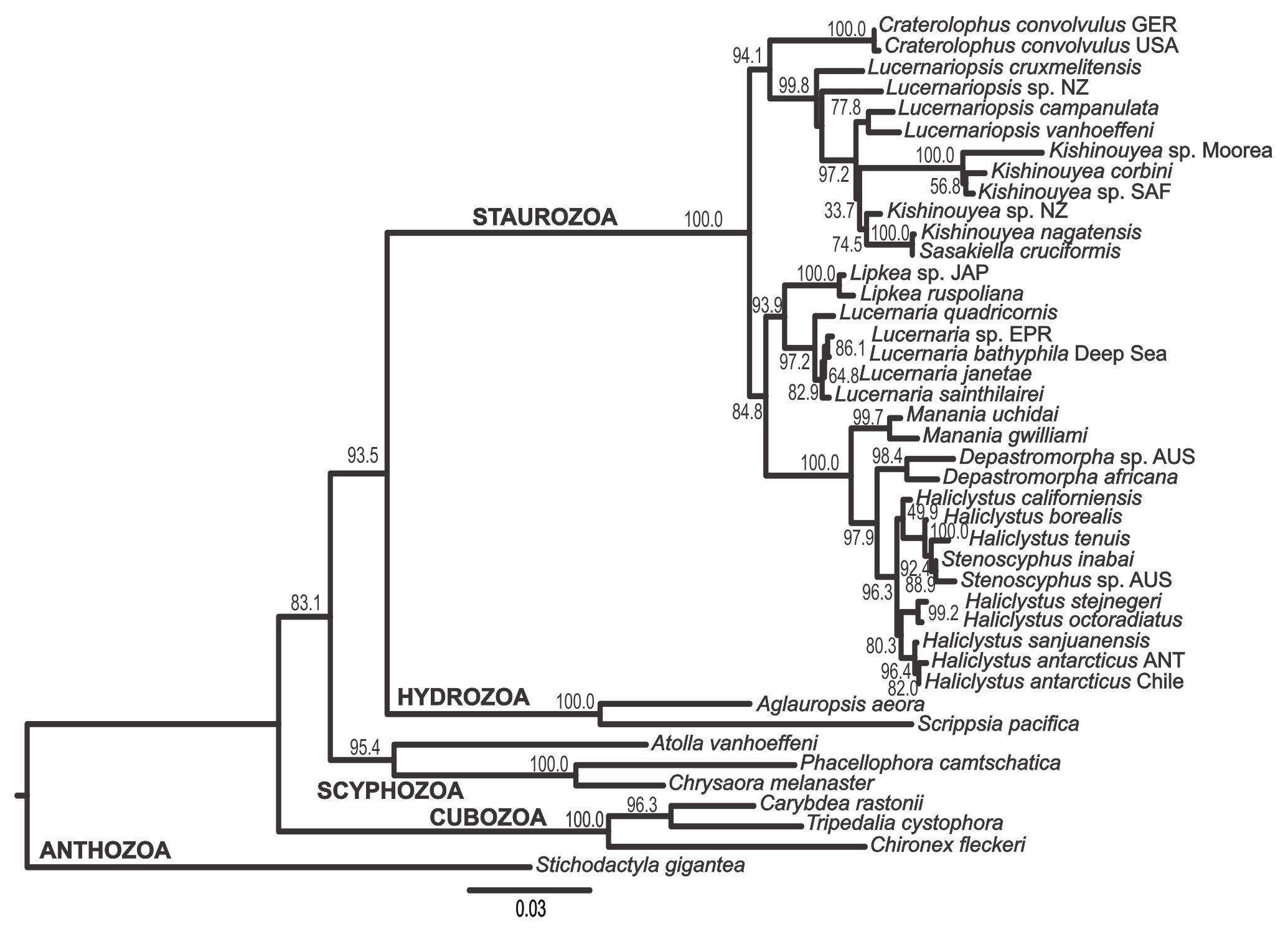

Supplement: Supplemental Information 10 — Bootstrap indices under maximum likelihood at each node. ANT, Antarctica; AUS, Australia; EPR, East Pacific Rise; GER, Germany; JAP, Japan; NZ, New Zealand; SAF, South Africa; USA, the United States of America. [file peerj-04-1951-s010.png]

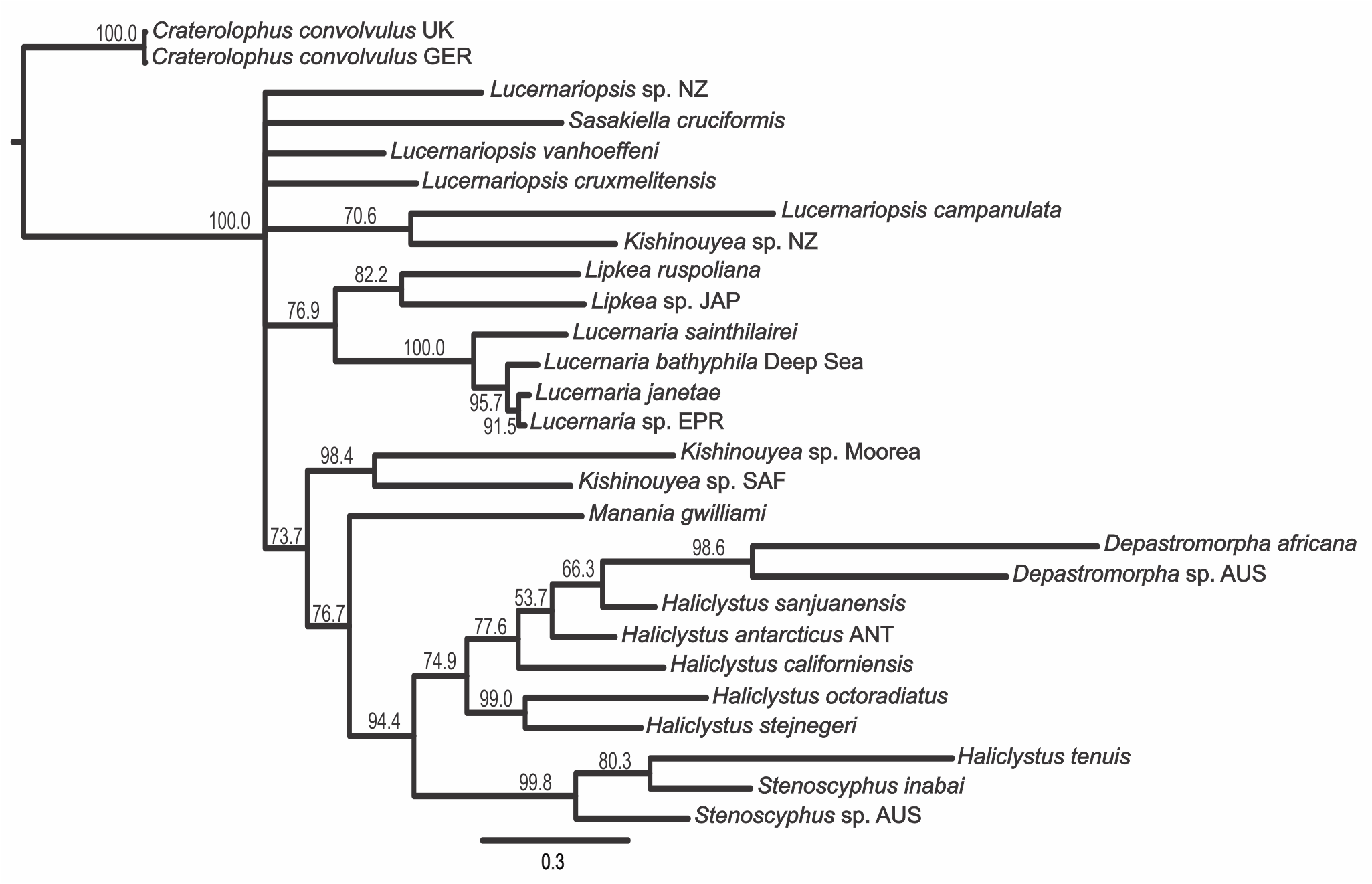

Supplement: Supplemental Information 11 — Posterior probability at each node. ANT, Antarctica; AUS, Australia; EPR, East Pacific Rise; GER, Germany; JAP, Japan; NZ, New Zealand; SAF, South Africa; UK, the United Kingdom. [file peerj-04-1951-s011.png]

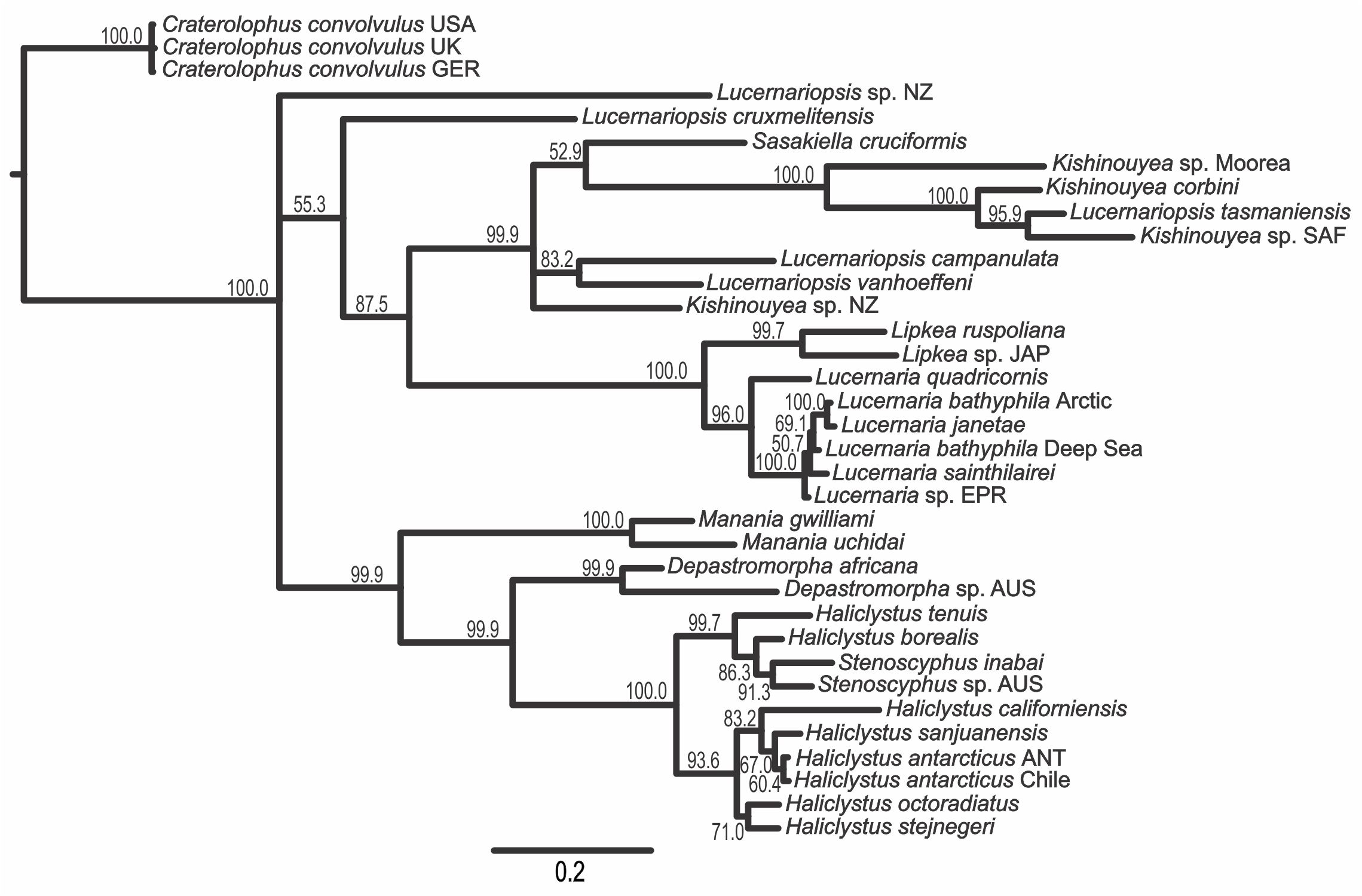

Supplement: Supplemental Information 12 — Posterior probability at each node. ANT, Antarctica; AUS, Australia; EPR, East Pacific Rise; GER, Germany; JAP, Japan; NZ, New Zealand; SAF, South Africa; UK, the United Kingdom; USA, the United States of America. [file peerj-04-1951-s012.png]

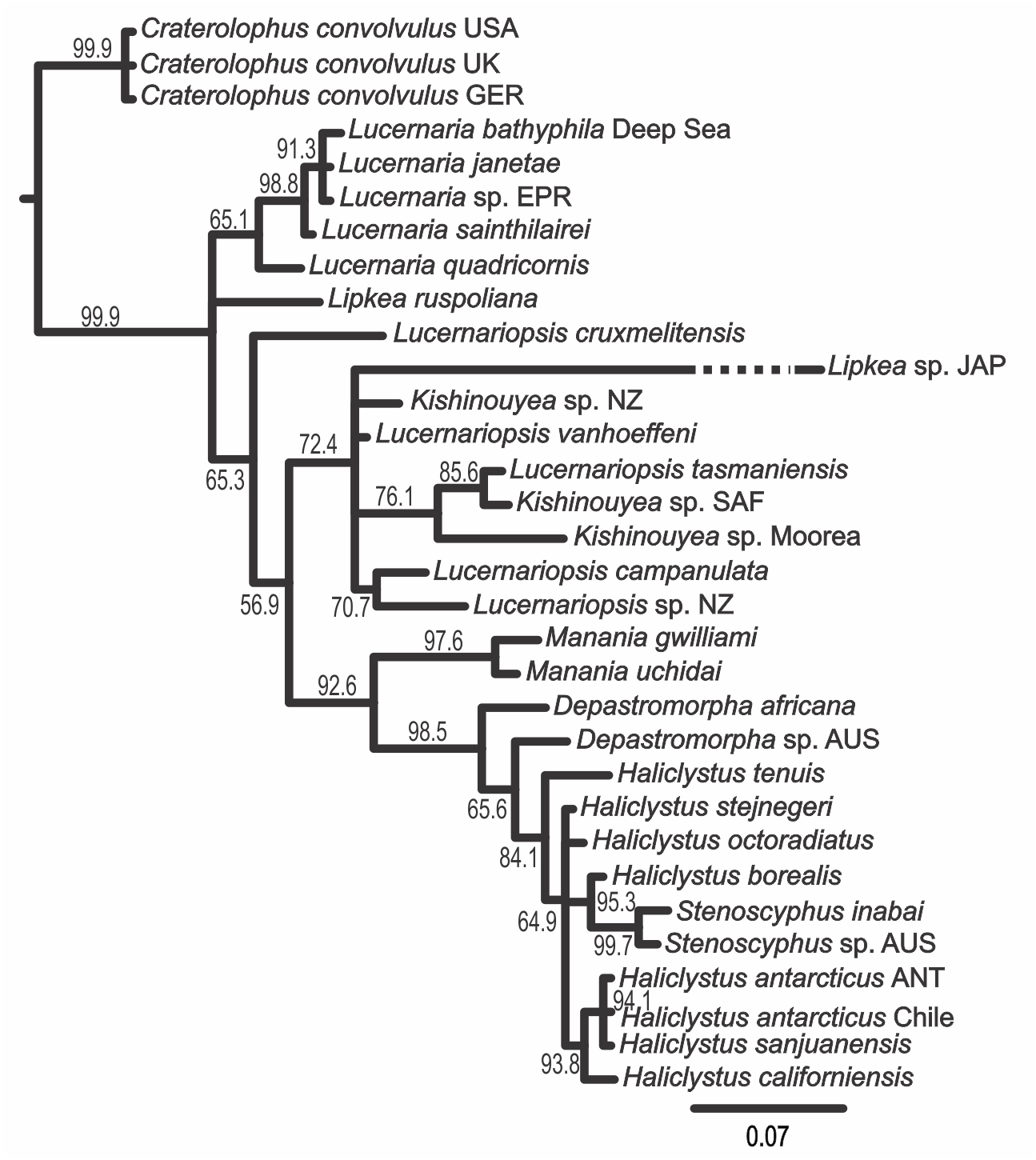

Supplement: Supplemental Information 13 — Posterior probability at each node. ANT, Antarctica; AUS, Australia; EPR, East Pacific Rise; GER, Germany; JAP, Japan; NZ, New Zealand; SAF, South Africa; UK, the United Kingdom; USA, the United States of America. [file peerj-04-1951-s013.png]

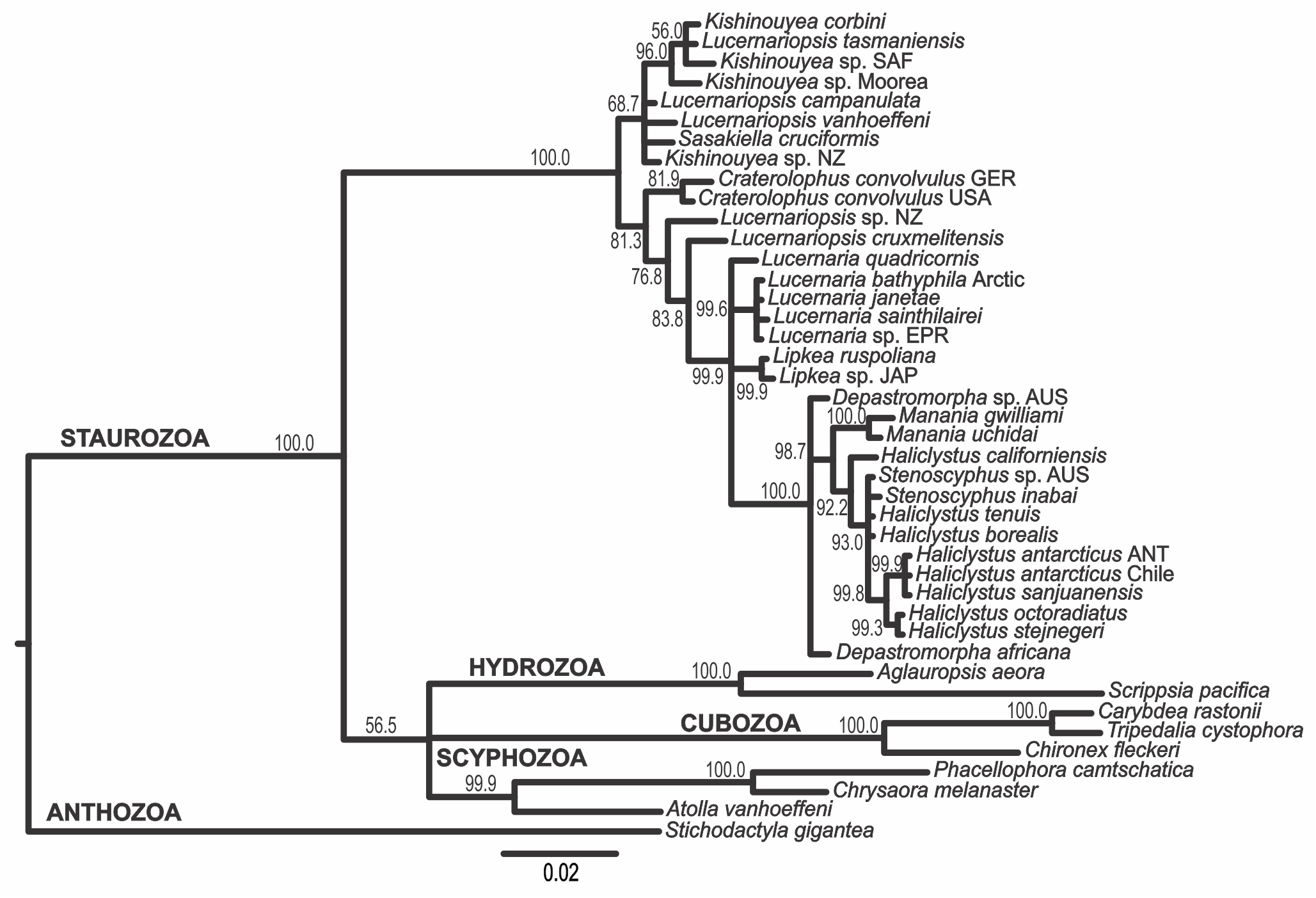

Supplement: Supplemental Information 14 — Posterior probability at each node. ANT, Antarctica; AUS, Australia; EPR, East Pacific Rise; GER, Germany; JAP, Japan; NZ, New Zealand; SAF, South Africa; USA, the United States of America. [file peerj-04-1951-s014.png]

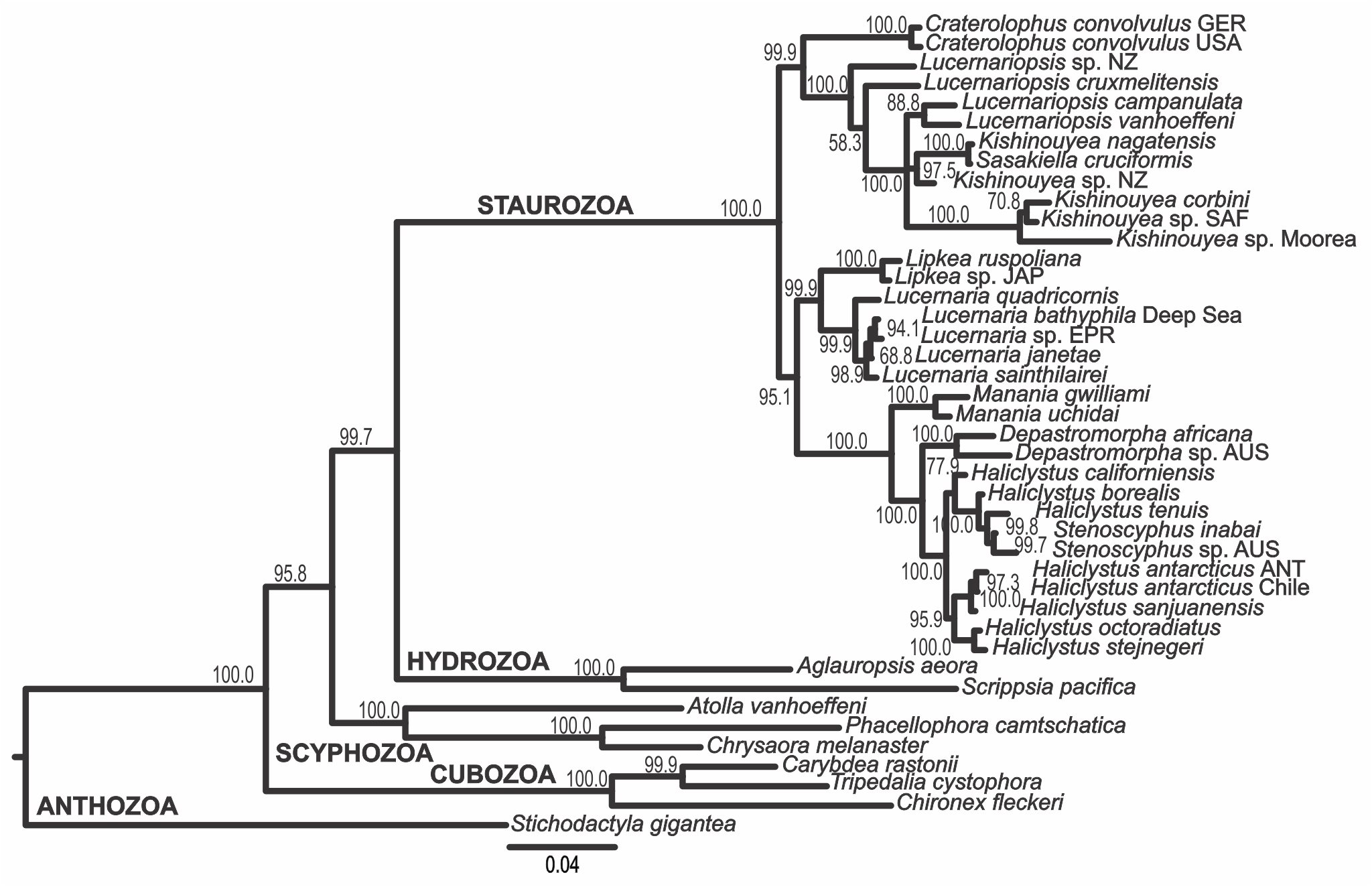

Supplement: Supplemental Information 15 — Posterior probability at each node. ANT, Antarctica; AUS, Australia; EPR, East Pacific Rise; GER, Germany; JAP, Japan; NZ, New Zealand; SAF, South Africa; USA, the United States of America. [file peerj-04-1951-s015.png]
